# *Cata*‐Annulated Azaacene Bisimides

**DOI:** 10.1002/chem.202101573

**Published:** 2021-07-29

**Authors:** Maximilian Elter, Lukas Ahrens, Stella M. Luo, Frank Rominger, Jan Freudenberg, Dennis D. Cao, Uwe H. F. Bunz

**Affiliations:** ^1^ Organisch-Chemisches Institut Ruprecht-Karls-Universität Heidelberg Im Neuenheimer Feld 270 69120 Heidelberg Germany; ^2^ Chemistry Department Macalester College Saint Paul MN 55105 USA

**Keywords:** azaacenes, butterfly dimer, bisimides, electron acceptors, heteroacenes

## Abstract

Ultra‐electron‐deficient azaacenes were synthesized via Buchwald‐Hartwig coupling of *ortho‐*diaminoarenes with chlorinated mellophanic diimide followed by oxidation of the intermediate *N*,*N’*‐dihydro compounds with MnO_2_ or PbO_2_. The resulting *cata*‐annulated bisimide azaacenes have ultrahigh electron affinities with first reduction potentials as low as −0.35 V recorded for a tetraazapentacene. Attempts to prepare a tetrakis(dicarboximide)tetraazaheptacene resulted in the formation of a symmetric butterfly dimer.

## Graphical abstract

**How high can you go?***Cata*‐annulation of dicarboximides onto azaacenes lowers the lowest unoccupied molecular orbitals and increases reduction potentials up to −0.35 V vs. Fc/Fc^+^, but does not stabilize a tetrakis(dicarboximide)tetraazaheptacene which dimerized regioselectively to its butterfly adduct.

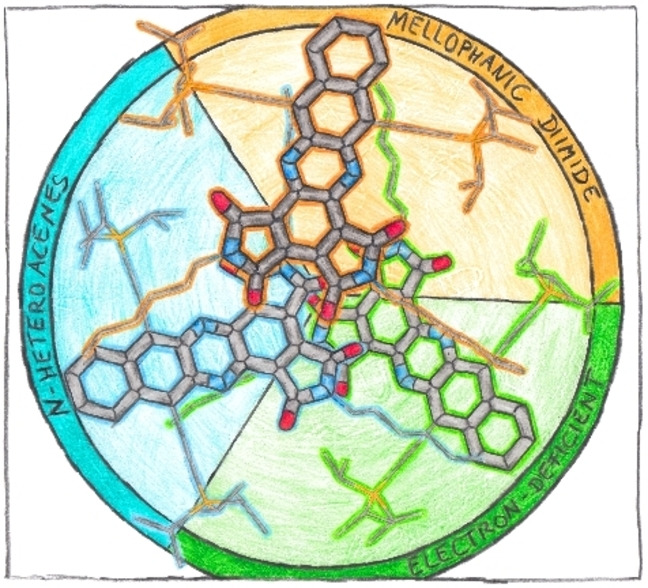

There is a constant push in materials science to prepare organic materials with extreme and attractive properties for uses that range from solar cells and n‐channel materials to spintronics. Attachment of bisimides to organic cores is known to a) increase their solubility,^[1]^ b) boost their electron affinity,^[2]^ c) stabilize radical anion states^[3]^ and d) push their *λ*_max_ into the red.^[4]^ While there have been reports of the juxtaposition of arenes with bisimides,^[5]^ the multiple *cata*‐annulation of azaacenes with imides has not been described despite the potential to furnish materials with unprecedented properties. Here we present the synthesis and characterization of novel azaacene derivatives with ultrahigh electron affinities (−3.88 eV to −4.35 eV) and near‐IR absorptions. These features are enabled by the presence of two or four bisimide rings at ends of the azaacenes.

Previously, Cao et al. prepared electron‐accepting and chromophoric heteroacenes by nucleophilic aromatic substitution of dichloro mellophanic diimide (**MDI**‐**Cl_2_**) with *ortho* dinucleophiles.^[6]^ Here, we couple **MDI**‐**Cl_2_** ^[7]^ with aromatic diamines^[8]^ to explore novel electron‐deficient azaacenes. Buchwald‐Hartwig coupling of diamines **6**–**9** with **MDI**‐**Cl_2_** (Scheme 1) using Pd‐RuPhos(G1)^[9]^ as catalyst and Cs_2_CO_3_ as base furnished **1**‐**H_2_**, **2**‐**H_2_**, **3**‐**H_2_** and **4**‐**H_2_** in 43 %–87 % yield, respectively. Although the diamine **10** did not couple under these conditions,^[10]^ switching to Pd_2_(dba)_3_ with RuPhos^[11]^ in Hünig's base as solvent gave **5**‐**H_2_** in 60 % yield (Scheme 1). The Pd‐catalyst is necessary for this coupling since its omission leads to decomposition of the starting diamines. Oxidation of the dihydro compounds with MnO_2_ or PbO_2_ furnishes the heteroacenes in 92 %‐97 %. Oxidation was more difficult for the larger and/or more electron‐poor starting *N,N’‐*dihydroazaacenes. Compounds **1**‐**H_2_** and **2**‐**H_2_** could be oxidized with MnO_2_ at room temperature (rt) while **3**‐**H_2_** needed to be heated to 50 °C to achieve oxidation. The even more electron‐poor **4**‐**H_2_** could only be oxidized by PbO_2_ at 70 °C in chloroform. For **5 a**, PbO_2_ at rt was sufficiently oxidizing, but the reaction had to be carried out under N_2_ because reduction back to **5**‐**H_2_** occurred spontaneously in air, similar to what has been previously observed for similarly electron‐poor dicyanodiazahexacene^[12]^ and hexaazahexacene.^[8a]^ This spontaneous reduction is a testament to the high electron affinities of **4 a** and **5 a**.

**Scheme 1:**
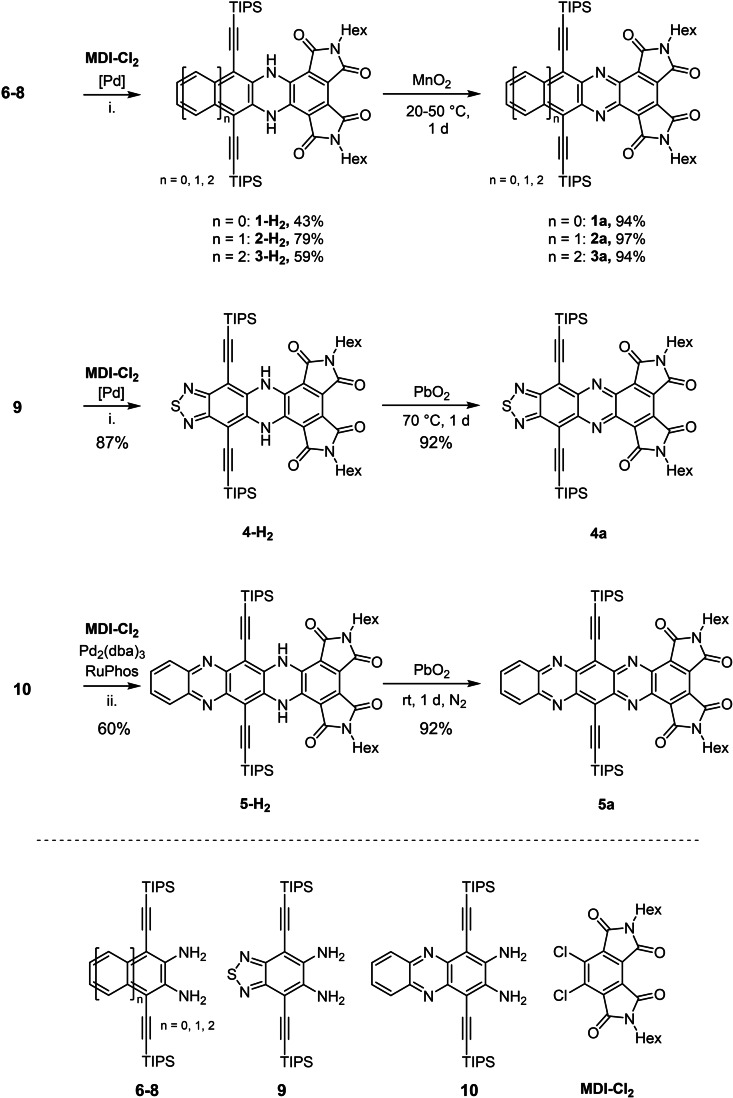
Synthesis of **1 a**–**5 a** via Buchwald‐Hartwig coupling of **6**–**10** with **MDI**‐**Cl_2_** and the subsequent oxidation of the dihydro species. [Pd]: Pd‐RuPhos(G1), i. Cs_2_CO_3_, toluene, 16 h 120 °C, ii. Hünig's base, 120 °C, 16 h.

Compounds **1 a**–**5 a** display red‐shifted absorption p‐bands compared to those of the unsubstituted azaacenes (Figure 1; 4545 cm^−1^ (**1 a**) to 2521 cm^−1^ (**3 a**)), with **3 a** and **5 a** being NIR absorbers. The absorption bands between 300 and 400 nm arise from the bisimide derivatization. Absorption maxima increase with azaacene size (series **1 a**–**3 a**) and **3 a** displays a *λ*_max_ of 908 nm.

**Figure 1:**
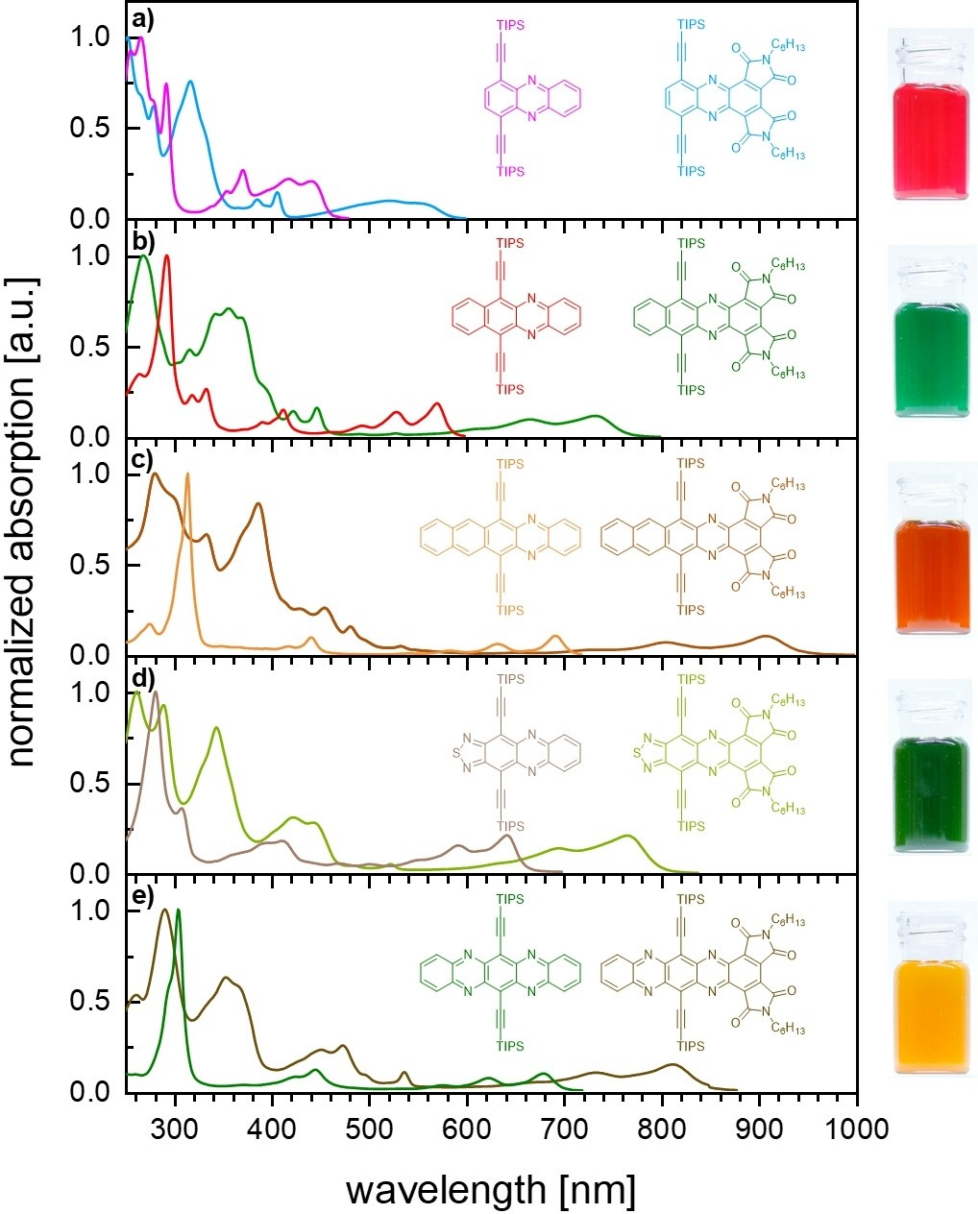
Left: Absorption spectra of azaacene bisimides and their parent compounds in *n*‐hexane. Right: Photographs of the azaacene bisimides in dichloromethane. From top to bottom: **1 a** to **5 a**.

First reduction potentials and electron affinities (cyclic voltammetry, CV, Figure S2, Supporting Information) also increase with azaacene size and number of nitrogen atoms. Interestingly, *cata*‐condensed bisimides are more powerful than CN‐substituents, as evidenced by a comparison of the first reduction potentials of **3 a** (−0.62 V) to that of a dicyanodiazaacene (−0.7 V).^[12]^ The even more electron accepting **4 a** and **5 a** are reduced at −0.36 V and −0.35 V (vs. Fc/Fc^+^), respectively. Together, the electrochemical data suggest that **2 a**, **3 a**, **4 a** and **5 a** should be isolable as air‐stable radical anions due to their high electron affinities.^[13]^ DFT‐calculated energy gaps are in agreement with optical gaps and the LUMO energies correlate with reduction potentials linearly (Table 1). Bisimide substitution lowers LUMO energies of azaacenes between 0.52 eV (**4 a**/**4 b**) and 0.87 eV (**5 a**/**5 b**) (Table 1), while the effect on the HOMO positions is less pronounced (0.26 eV for **4 a**/**4 b**; 0.51 eV for **5 a**/**5 b**), an observation that is consistent with the red‐shifted absorption bands. The FMOs (Figure 2) explain this qualitatively: Orbital coefficients of the LUMO extend to the bisimides, whereas those of the HOMO reside on the acene backbone and are thus less affected by annulation. In contrast to benzannulated acenes, NICS(1)_zz_ calculations show that the outer rings of the azaacene core are aromatic (*δ*=−17.6 to −21.5 ppm) and an integral part of the acene backbone, whereas the maleimides are antiaromatic (*δ*=8.70 to 9.39 ppm) (see Figure S7, Supporting Information).

**Table 1:** Optical, electrochemical and quantum‐chemical data of the azaacene bisimides and their parent compounds.

Compound	*E*^(0/−)^ [V]^[a]^	EA [eV]^[b]^	*E*_LUMO, DFT_ [eV]^[c]^	IP [eV]^[d]^	*E*_HOMO, DFT_ [eV]^[c]^	gap_DFT_ [eV]	*λ*_max, abs_ [nm]	opt. gap [eV]^[e]^
**1a**	−0.82	−3.88	−3.92	−5.93	−6.27	2.35	550	2.05
**1b** ^[f]^	−1.68	−3.02	−3.08	−5.72	−5.97	2.88	440	2.67
**2a**	−0.67	−4.03	−4.04	−5.59	−5.83	1.79	733	1.56
**2b** ^[f]^	−1.23	−3.47	−3.35	−5.59	−5.54	2.20	570	2.09
**3a**	−0.62	−4.08	−4.10	−5.47	−5.50	1.40	908	1.31
**3b** ^[f]^	−1.05	−3.65	−3.50	−5.40	−5.25	1.75	692	1.74
**4a**	−0.36	−4.34	−4.37	−5.89	−6.03	1.66	766	1.55
**4b** ^[g]^	−0.83	−3.87	−3.85	−5.73	−5.77	1.82	642	1.86
**5a**	−0.35	−4.35	−4.30	−5.79	−5.80	1.50	813	1.44
**5b** ^[h]^	−0.79	−3.67	−3.43	−5.49	−5.29	1.86	680	1.82

[a] First reduction potentials measured by cyclic voltammetry (CV) in dichloromethane with Bu_4_NPF_6_ as the electrolyte against Fc/Fc^+^ as an internal standard at 0.2 V s^−1^. The offset of the exact values is estimated by adjusting the ferrocene redox couple to −5.10 eV^[14]^ on the Fermi scale, for azaacenes EA=−(*E* ^0/−^_[vs. Fc+/Fc]_+4.70 eV) gives a much better empirical fit of almost all CV data to DFT calculations.[^12^, ^15^] A table referencing ferrocene as −5.10 eV can be found in the Supporting Information (Table S1). [b] Calculated from CV measurements EA=−(*E* ^0/−^_[vs. Fc+/Fc]_+4.7 eV).^[14]^ [c] Obtained from quantum‐chemical calculations with DFT/B3LYP/def2‐TZVP. [d] IP=*E*_A_ ‐ opt. gap. [e] Calculated from *λ*_onset, abs_. [f] CV data and quantum‐chemical calculations taken from Ref. [16] and adjusted for Fc/Fc^+^=−4.70 eV. [g] CV data and quantum‐chemical calculations taken from Ref. [17] and adjusted for Fc/Fc^+^=−4.70 eV. [h] CV data and quantum‐chemical calculations taken from Ref. [18] and adjusted for Fc/Fc^+^=−4.70 eV.

**Figure 2:**
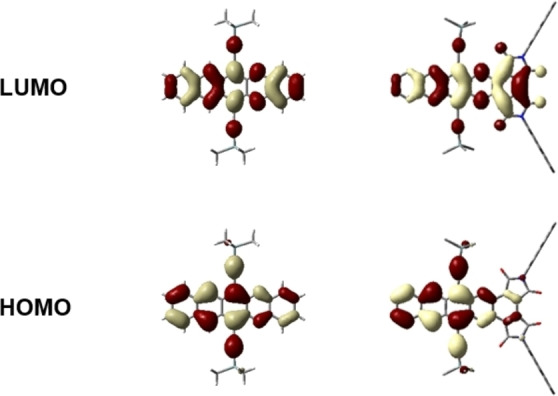
Comparison of the DFT‐calculated HOMOs and LUMOs [B3LYP, def2TZVP] of 3 a* to 3 b* (TIPS‐substituents were replaced by TMS‐substituents).

Single crystals X‐ray analysis of **2 a** reveals molecules packed into a chiral helix of trigonal symmetry (Figure 3). Intermolecular interactions are dominated by the imide groups; carbonyl oxygens are in close contact with neighbouring partially positively charged carbon atoms. The one‐helix turn distance between the azaacene planes is 7.46 Å. The non‐overlapping acene backbones exhibit end‐to‐end tilts of 15°. The absolute structure could not be determined as there are two possible enantiomers for the chiral space group (Table S7, Supporting Information).

**Figure 3:**
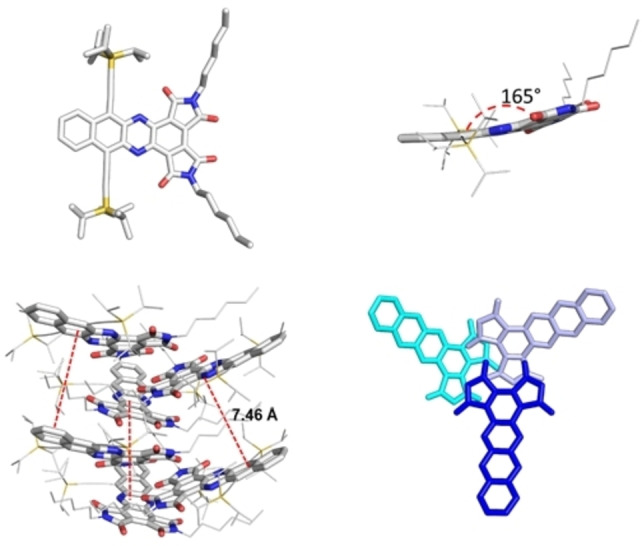
Crystal structure and packing of **2 a**. TIPS‐ethynyl and hexyl substituents were either reduced in size or omitted for clarity.

To evaluate whether azaheptaacenes would exhibit similarly improved properties, Müllen's tetraaminoanthracene **11** ^[8f]^ was coupled to **MDI**‐**Cl_2_** using a Buchwald‐Hartwig reaction to yield the tetrahydro compound **12**‐**H_4_**. PbO_2_ oxidation of **12**‐**H_4_** in dry DCM furnished **13**, a dimer of the intended product **12**, as a green solid. ^1^H NMR spectroscopy suggests a non‐centrosymmetric constitution of C_2h_ symmetry, represented either by the head‐to‐tail [4+4] dimer as shown in Scheme 2 or its head‐to‐head isomer (Figure S1, Supporting Information). This phenomenon is similar to the products of photochemical dimerization of TIPS‐pentacene.^[19]^ Attempts to isolate **12** were unsuccessful, indicating that the dimerization is fast on the experimental timescale.

**Scheme 2:**
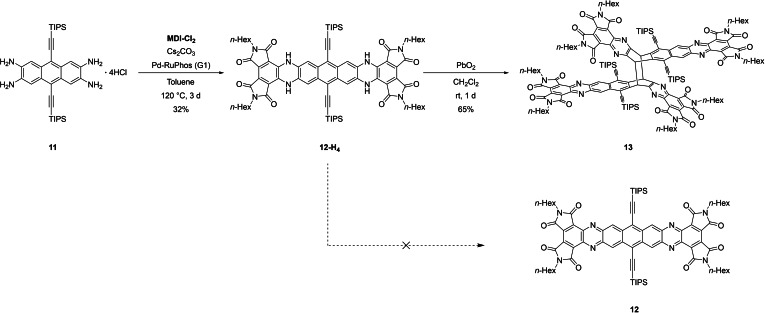
Attempted synthesis of a tetraazaheptacene **12** and isolation of its [4+4] degradation product **13** (one geometric isomer shown).

High‐resolution MALDI mass spectrometry confirms the dimeric constitution, in addition to providing evidence that retro cycloaddition to the heptacene **12** occurs under these conditions. The absorption spectrum of **13** is a close match to that of **2 a** (Figure S5, Supporting Information), which can be rationalized by noting that the largest conjugated chromophore in **13** is structurally analogous to **2 a**.

## Conclusion

The addition of bisimides to the terminal ring of azaacenes using a simple coupling‐oxidation sequence results in near‐IR absorbers with high electron affinities up to −4.35 eV. Doubly or quadruply bisimide‐substituted azapentacenes are stable, processable, yet amorphous materials that should be almost ideal materials for solar cells, and, upon shortening the hexyl chains, may also give attractive n‐channel semiconductors for thin film transistors, which we will report upon in the future.

## Experimental Section

### General Procedure (GP)

The corresponding *ortho*‐diamine (1.00 eq.), **MDI**‐**Cl_2_** (1.00 eq.), caesium carbonate (3.00 eq) and Pd‐RuPhos G1 (5 mol %) were placed in a dry Schlenk tube under Argon atmosphere. Then dry and degassed toluene (1 mL per 50 mg diamine) was added and the reaction was stirred at 120 °C for 16 h. The reaction mixture was diluted with DCM and water was added. The phases were separated and the aqueous layer was extracted with methylene chloride (3×10 mL). The combined organic layers were dried over MgSO_4_, filtered and the solvent was removed under reduced pressure.

### 2,5‐Dihexyl‐8,11‐bis((triisopropylsilyl)ethynyl)‐7,12‐dihydrodipyrrolo[3,4‐*a*:3′,4′‐*c*]phenazine‐1,3,4,6(2*H*,5*H*)‐tetraone (1‐H_2_)

The **GP** was applied to 3,6‐bis((triisopropylsilyl)ethynyl)benzene‐1,2‐diamine (51.7 mg, 110 μmol, 1.00 eq.) and MDI‐Cl_2_ (50.0 mg, 110 μmol, 1.00 eq.), using caesium carbonate (108 mg, 331 μmol, 3.00 eq.) and Pd‐RuPhos (G1) (4.50 mg, 5.51 μmol, 0.05 eq.). The resulting crude product was purified by column chromatography (SiO_2_ PE/DCM 1 : 1) yielding **1**‐**H_2_** as a red solid (40.2 mg, 47.3 μmol, 43 %). ^1^H NMR (400 MHz, CDCl_3_) δ [ppm]=8.49 (s, 2H), 6.69 (s, 2H), 3.59 (t, J=7.0 Hz, 4H), 1.62 (quin, J=6.6 Hz, 4H), 1.32–1.11 (m, 58H), 0.92–0.83 (m, 7H). ^13^C NMR (101 MHz, CDCl_3_) δ [ppm]=167.8, 163.9, 136.6, 131.0, 126.4, 120.8, 110.0, 109.0, 101.9, 99.4, 38.2, 31.5, 28.3, 26.5, 22.6, 18.8, 14.2, 11.4. HRMS (MALDI+, DCTB): m/z calcd. for C_50_H_72_N_4_O_4_Si_2_: [M]^+^ 848.5092, found: 848.5085, correct isotope distribution. IR (ATR) $\tilde{v}$ [cm^−1^]=3320, 2927, 2859, 2363, 1759, 1691, 1540, 1380, 802, 776, 675, 660, 619, 609, 595, 442, 410. m.p.=180 °C.

### 2,5‐Dihexyl‐8,11‐bis((triisopropylsilyl)ethynyl)dipyrrolo[3,4‐*a*:3′,4′‐*c*]phenazine‐1,3,4,6(2*H*,5*H*)‐tetraone (1 a)

**1**‐**H_2_** (30.0 mg, 35.3 μg, 1.00 eq.) was dissolved in methylene chloride and an excess of manganese dioxide was added. The reaction mixture was stirred at room temperature until TLC showed full consumption of the dihydro species. The mixture was filtered and the solvent was removed under reduced pressure to yield **1 a** as a red solid (28.1 mg, 33.2 μmol, 94 %).^1^H NMR (600 MHz, CDCl_3_) δ [ppm]=8.14 (s, 2H), 3.83 (t, J=7.2 Hz, 4H), 1.79–1.72 (m, 4H), 1.35–1.24 (m, 54H), 0.90–0.87 (m, 6H). ^13^C NMR (151 MHz, CDCl_3_) δ [ppm]=164.6, 164.5, 145.2, 139.7, 137.9, 134.4, 130.2, 125.6, 103.6, 102.0, 38.7, 31.3, 28.3, 26.4, 22.4, 18.8, 14.1, 11.5. HRMS (MALDI+, DCTB): m/z calcd. for C_50_H_71_N_4_O_4_Si_2_: [M+H]^+^ 847.5008, found: 847.5038, correct isotope distribution. IR (ATR) $\tilde{v}$ [cm^−1^]=2923, 2864, 1771, 1722, 1713, 1463, 1398, 1364, 1064, 996, 881, 791, 676, 659, 589, 462, 456, 418. m.p.=174 °C.

### 2,5,13,16‐Tetrahexyl‐9,20‐bis([tri(propan‐2‐yl)silyl]ethynyl)‐7,11,18,22‐tetrahydrotetrapyrrolo[3,4‐*h*:3,4‐*h*′:3,4‐*j*:3,4‐*j*′]benzo[1,2‐*b*:4,5‐*b*′]diphenazine‐1,3,4,6,12,14,15,17(2*H*,5*H*,13*H*,16*H*)‐octone (12‐H_4_)

The **GP** was applied to Müllen's 9,10‐bis((triisopropylsilyl)ethynyl)‐anthracene‐2,3,6,7‐tetraaminiumchloride (100 mg, 134 μmol, 1.00 eq.) and **MDI**‐**Cl_2_** (137 mg, 302 μmol, 2.25 eq), using caesium carbonate (875 mg, 2.69 mmol, 20.0 eq.) and PdRuPhos (G1) (11.0 mg, 13.4 μmol, 0.10 eq.). The resulting crude product was washed with methanol and PE and then purified via GPC yielding **12**‐**H_4_** as a dark purple solid (59.3 mg, 42.9 μmol, 32 %). ^1^H NMR (600 MHz, CD_2_Cl_2_) δ [ppm]=8.46 (s, 4H), 7.36–7.34 (m, 4H), 3.63 (br t, J=7.2 Hz, 8H), 1.68–1.63 (m, 8H), 1.34–1.29 (m, 66H), 0.90 (br t, J=6.9 Hz, 12H). ^13^C NMR (151 MHz, CD_2_Cl_2_) δ [ppm]=168.8, 164.0, 134.4, 131.3, 129.6, 120.00, 114.7, 111.4, 108.9, 102.9, 31.7, 28.7, 26.8, 22.8, 19.0, 14.1, 11.9. HRMS (MALDI+, DCTB): m/z calcd. for C_80_H_103_ N_8_O_8_Si_2_: [M+H]^+^ 1359.7432, found: 1359.7429, correct isotope distribution. IR (ATR) $\tilde{v}$ [cm^−1^]=3345, 2924, 2854, 2362, 2341, 2331, 1769, 1698, 1685, 1570, 1498, 1465, 1364, 1278, 778, 758, 733, 691, 678, 550. m.p.=>330 °C.

### Butterfly‐Dimer (13)

**12**‐**H_4_** (2.00 mg, 1.47 μmol, 1.00 eq) was dissolved in DCM (0.5 mL) in a nitrogen glove box. Then an excess of PbO_2_ was added. The reaction was stirred at rt for 1 d. The reaction mixture was filtered to yield **13** as a green solid (2.60 mg, 0.96 μmol, 65 %). ^1^H NMR (600 MHz, CD_2_Cl_2_) δ [ppm]=9.51 (s, 4H), 6.64–6.47 (m, 4H), 3.81 (br t, J=7.3 Hz, 8H), 3.70–3.61 (m, 8H), 1.80–1.73 (m, 8H), 1.70–1.46 (m, 126H), 1.45–1.39 (m, 14H), 0.91–0.87 (m, 24H). ^13^C NMR (151 MHz, CD_2_Cl_2_) δ [ppm]=165.7, 165.0, 164.3, 164.1, 159.6, 142.3, 141.3, 139.6, 139.0, 135.2, 135.1, 133.6, 131.1, 131.0, 129.8, 122.3, 110.5, 101.1, 57.8, 39.2, 39.0, 32.0, 31.8, 29.0, 28.8, 27.1, 27.0, 23.1, 23.0, 19.6, 19.5, 14.4, 12.3. HRMS (MALDI+, DCTB): m/z calcd. for C_160_H_196_N_16_O_16_Si_4_: [M/2]^⋅+^ 1354.7046, found: 1354.7027, correct isotope distribution. IR (ATR) $\tilde{v}$ [cm^−1^]=2955, 2922, 2853, 2359, .2341, 1773, 1725, 1458, 1391, 1376, 1365, 1259, 1083, 1014, 880, 702, 679, 670, 661, 403.

### Crystallographic data

Deposition Number(s) 2080832 (**2 a**) and 2080833 (**3**‐**H**_**2**_) contain(s) the supplementary crystallographic data for this paper. These data are provided free of charge by the joint Cambridge Crystallographic Data Centre and Fachinformationszentrum Karlsruhe Access Structures service www.ccdc.cam.ac.uk/structures.

## Conflict of interest

The authors declare no conflict of interest.

## Supporting information

As a service to our authors and readers, this journal provides supporting information supplied by the authors. Such materials are peer reviewed and may be re‐organized for online delivery, but are not copy‐edited or typeset. Technical support issues arising from supporting information (other than missing files) should be addressed to the authors.

Supporting Information
