# Supplementary material for: Cata‐Annulated Azaacene Bisimides
Source: Chemistry. 2021 Jul 29;27(48):12284–8. doi: 10.1002/chem.202101573 (PMC8457205; doi:10.1002/chem.202101573)
Supplement: Supplementary file 1 — Supporting Information [file CHEM-27-12284-s001.pdf]

Chemistry–A European Journal

Supporting Information

Cata-Annulated Azaacene Bisimides

Maximilian Elter, Lukas Ahrens, Stella M. Luo, Frank Rominger, Jan Freudenberg,*
Dennis D. Cao,* and Uwe H. F. Bunz*

Table of Contents

1) General Remarks S2
2) Synthesis S3
3) Cyclic Voltammetry S11
4) Optical Spectroscopy S12
5) Geometry and Energy computation S14
6) FMO Distribution S26
7) NICS(1)zz calculations S27
8) AICD Calculations S28
9) NMR Spectroscopy S31
10) Infrared Spectroscopy S55
11) HRMS Spectra S59
12) Crystallographic Data S65
13) References S67

WILEY-VCH

1) General Remarks

TLC was performed using polyester sheets coated with silica gel produced by Macherey-Nagel & Co. Polygram® SIL g/UV254). Column chromatography was performed using silica gel from Macherey, Nagel & Co. (particle size: 0.032–0.062 mm). Preparative GPC was performed on Bio-Beads® (S-X1 Beads, 200-400 Mesh, crosslinked polystyrene), purchased from Bio-Rad Laboratories, Inc.. CHROMAFIL® Xtra PTFE-45/25 syringefilters by Macherey-Nagel with a pore size of 0.45 µm and 25 mm diameter were used as syringefilters. IR spectra were recorded at room temperature on a Jasco FT/IR-4100 spectrometer. Melting points were determined in open glass capillaries on a Melting Point Apparatus *MEL-TEMP* (Electrothermal, Rochford, UK) and are uncorrected. NMR spectra were recorded on Bruker Avance spectrometers using the specified frequency at 295 K. Chemical shifts (δ) are given in parts per million (ppm) relative to internal solvent signals.[S1] The following abbreviations describe the signal multiplicities: s = singlet, d = doublet, t = triplet, quin = quintet, dd = doublet of doublets, m = multiplet, br = broad signal. High-resolution mass spectra (HRMS) were obtained by matrix-assisted laser desorption/ionization (MALDI) using *trans*-2-[3-(4-*tert*-butylphenyl)-2-methyl-2-propenylidene]-malononitrile (DCTB) as matrix, electrospray ionisation (ESI) or direct analysis in real time (DART) experiments. CV measurements were performed on a VersaSTAT 3 potentiostat by Princeton Applied Research. UV-vis spectra were recorded on a Jasco V670. Computational studies were carried out using DFT calculations on Gaussian16 and GaussView 6. Geometry optimizations were performed using the B3LYP functional and def2SVP basis set. At this geometry, the absolute energy and FMO energies were determined by a single-point approach at the B3LYP/def2TZVP level.[S2]

The diamine-building blocks used were synthesized according to literature procedures:

4,5-Dichloro-2,7-dihexylpyrrolo[3,4-*e*]isoindole-1,3,6,8(2*H*,7*H*)-tetraone[S3]
3,6-Bis((triisopropylsilyl)ethynyl)benzene-1,2-diamine,[S4]
1,4-bis((triisopropylsilyl)ethynyl)naphthalene-2,3-diamine,[S5]
1,4-bis((triisopropylsilyl)ethynyl)anthracene-2,3-diamine,[S6]
1,4-bis((triisopropylsilyl)ethynyl)phenazine-2,3-diamine,[S7]
4,7-bis((triisopropylsilyl)ethynyl)-benzo[*c*][1,2,5]thiadiazole-5,6-diamine,[S8]
9,10-bis((triisopropylsilyl)ethynyl)-anthracene-2,3,6,7-tetraaminiumchloride.[S9]

WILEY-VCH

2) Synthesis

General Procedure (GP)

The corresponding *ortho*-diamine (1.00 eq), **MDI-Cl$_2$** (1.00 eq), cesium carbonate (3.00 eq.) and Pd RuPhos G1 (5 mol%) were placed in a dry Schlenk tube under Argon atmosphere. Then dry and degassed toluene (1 mL per 50 mg diamine) was added and the reaction was stirred at 120 °C for 16 h. The reaction mixture was diluted with dichloromethane and water was added. The phases where separated and the aqueous layer was extracted with methylene chloride (3x10 mL). The combined organic layers were dried over $MgSO_4$, filtered through filter paper and the solvent was removed under reduced pressure.

2,5-Dihexyl-8,11-bis((triisopropylsilyl)ethynyl)-7,12-dihydrodipyrrolo[3,4-*a*:3',4'-*c*]phenazine-1,3,4,6(2*H*,5*H*)-tetraone (1-H$_2$)

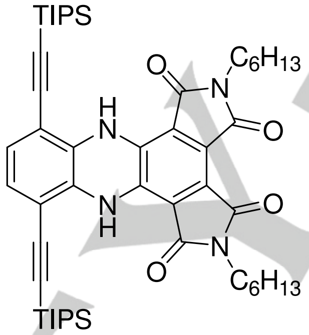

GP was applied to 3,6-bis((triisopropylsilyl)ethynyl)benzene-1,2-diamine **6** (51.7 mg, 110 µmol, 1.00 eq.) and **MDI-Cl$_2$** (50.0 mg, 110 µmol, 1.00 eq.), using cesium carbonate (108 mg, 331 µmol, 3.00 eq.) and Pd-RuPhos (G1) (4.50 mg, 5.51 µmol, 5 mol%.). The resulting crude product was purified by column chromatography (SiO_2, PE/DCM 1:1) yielding **1-H$_2$** as a red solid (40.2 mg, 47.3 µmol, 43%). R_f (DCM:PE) = 0.27, ^{1}H NMR (400 MHz, $CDCl_3$) δ = 8.49 (s, 2H), 6.69 (s, 2H), 3.59 (t, *J* = 7.0 Hz, 4H), 1.62 (quin, *J* = 6.6 Hz, 4H), 1.32 - 1.11 (m, 58H), 0.92 - 0.83 (m, 7H). ^{13}C NMR (101 MHz, $CDCl_3$) δ = 167.8, 163.9, 136.6, 131.0, 126.4, 120.8, 110.0, 109.0, 101.9, 99.4, 38.2, 31.5, 28.3, 26.5, 22.6, 18.8, 14.2, 11.4. HRMS (MALDI+, DCTB): m/z calcd. for $C_{50}H_{72}N_4O_4Si_2$: $[M]^{\cdot+}$ 848.5092, found: 848.5085, correct isotope distribution. IR (ATR) ṽ [cm^{-1}] = 3320, 2927, 2859, 2363, 1759, 1691, 1540, 1380, 802, 776, 675, 660, 619, 609, 595, 442, 410. M.p. = 180 °C.

2,5-Dihexyl-8,11-bis((triisopropylsilyl)ethynyl)dipyrrolo[3,4-*a*:3',4'-*c*]phenazine-1,3,4,6(2*H*,5*H*)-tetraone (1a)

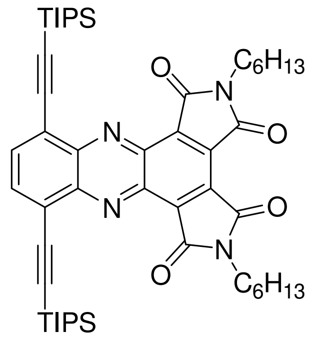

1-H₂ (30.0 mg, 35.3 µg, 1.00 eq.) was dissolved in methylene chloride and an excess of manganese dioxide (>100 eq.) was added. The reaction mixture was stirred at room temperature until TLC showed full consumption of the dihydro species. The mixture was filtered through a filter paper and the solvent was removed under reduced pressure to yield **1a** as a red solid (28.1 mg, 33.2 µmol, 94%). ^{1}H NMR (600 MHz, $CDCl_3$) δ = 8.14 (s, 2H), 3.83 (t, *J* = 7.2 Hz, 4H), 1.79 - 1.72 (m, 4H), 1.35 - 1.24 (m, 54H), 0.90 - 0.87 (m, 6H). ^{13}C NMR (151 MHz, $CDCl_3$) δ = 164.6, 164.5, 145.2, 139.7, 137.9, 134.4, 130.2, 125.6, 103.6, 102.0, 38.7, 31.3, 28.3, 26.4, 22.4, 18.8, 14.1, 11.5. HRMS (MALDI+, DCTB): m/z calcd. for $C_{50}H_{71}N_4O_4Si_2$: $[M+H]^+$ 847.5008, found: 847.5038, correct isotope distribution. IR (ATR) ṽ [cm^{-1}] = 2923, 2864, 1771, 1722, 1713, 1463, 1398, 1364, 1064, 996, 881, 791, 676, 659, 589, 462, 456, 418. M.p. = 174 °C.

2,5-Dihexyl-8,13-bis((triisopropylsilyl)ethynyl)-7,14-dihydrobenzo[*i*]dipyrrolo[3,4-*a*:3',4'-*c*]phenazine-1,3,4,6(2*H*,5*H*)-tetraone (2-H₂)

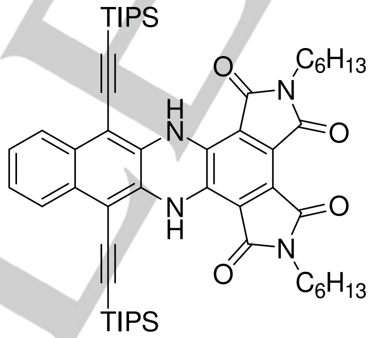

GP was applied to 1,4-bis((triisopropylsilyl)ethynyl)naphthalene-2,3-diamine **7** (57.2 mg, 110 µmol, 1.00 eq.) and **MDI-Cl₂** (50.0 mg, 110 µmol, 1.00 eq.), using cesium carbonate (108 mg, 331 µmol, 3.00 eq.) and PdRuPhos (G1) (4.50 mg, 5.51 µmol, 5 mol%). The resulting crude product was purified by column chromatography (SiO_2, PE/DCM 1:1) yielding **2-H₂** as a red solid (78.0 mg, 86.7 µmol, 79%). R_f (DCM:PE) = 0.33, ^{1}H NMR (600 MHz, $CDCl_3$) δ = 9.02 (s, 2H), 7.96 - 7.93 (m, 2H), 7.38 - 7.33 (m, 2H), 3.63 (t, *J* = 7.0 Hz, 4H), 1.65 (br t, *J* = 6.9 Hz, 4H), 1.38 - 1.19 (m, 54H), 0.93 - 0.82 (m, 6H). ^{13}C NMR (151 MHz, $CDCl_3$) δ = 167.9, 164.1, 134.6, 131.1, 126.8, 125.6, 120.4, 111.3, 107.1, 103.8, 98.0, 83.4, 38.2, 31.5, 28.3, 26.6, 22.6, 19.0, 14.2, 11.4. HRMS (MALDI+, DCTB): m/z calcd. for $C_{54}H_{74}N_4O_4Si_2$: $[M]^{\cdot+}$ 898.5249, found: 898.5254, correct isotope distribution. IR (ATR) ṽ [cm^{-1}] = 3310, 2921, 2861, 2359, 2359, 1698, 1540, 1508, 1456, 1380, 1352, 756, 670, 508, 410. Melting point: M.p. = 238 °C.

2,5-Dihexyl-8,13-bis((triisopropylsilyl)ethynyl)benzo[*i*]dipyrrolo[3,4-*a*:3',4'-*c*]phenazine-1,3,4,6(2*H*,5*H*)-tetraone (2a)

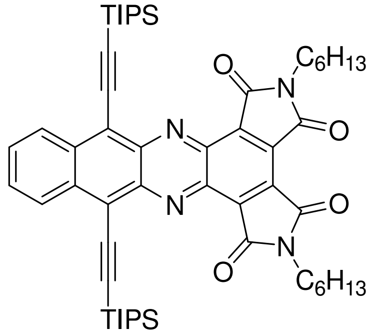

2-H₂ (40.0 mg, 44.5 µmol, 1.00 eq) was dissolved in methylene chloride and an excess of manganese dioxide (>100 eq.) was added. The reaction mixture was stirred at room temperature until TLC showed full consumption of the dihydro species. The mixture was filtered through a paper filter and the solvent was removed under reduced pressure to yield **2a** as a green solid (38.8 mg, 43.2 µmol, 97%). ^{1}H NMR (600 MHz, $CDCl_3$) δ = 8.86 - 8.81 (m, 2H), 7.78 - 7.70 (m, 2H), 3.85 (t, *J* = 7.2 Hz, 4H), 1.78 (quin, *J* = 7.3 Hz, 4H), 1.33 (d, *J* = 7.2 Hz, 54H), 0.89 (t, *J* = 7.0 Hz, 6H). ^{13}C NMR (151 MHz, $CDCl_3$) δ = 164.9, 164.8, 142.1, 140.0, 138.0, 134.8, 130.4, 129.9, 128.3, 122.8, 111.9, 101.8, 38.8, 31.5, 28.4, 26.6, 22.6, 19.1, 14.2, 11.8. HRMS (MALDI+, DCTB): m/z calcd. for $C_{54}H_{73}N_4O_4Si_2$: $[M+H]^+$ 897.5179, found: 897.5165, correct isotope distribution. IR (ATR) ṽ [cm^{-1}] = 2940, 2923, 2863, 1771, 1721, 1713, 1435, 1394, 1101, 995, 880, 761, 735, 673, 659, 576, 444, 414, 402. Melting point: M.p. = 209 °C.

2,5-Dihexyl-8,15-bis((triisopropylsilyl)ethynyl)-7,16-dihydronaphtho[2,3-*i*]dipyrrolo[3,4-*a*:3',4'-*c*]phenazine-1,3,4,6(2*H*,5*H*)-tetraone (3-H₂)

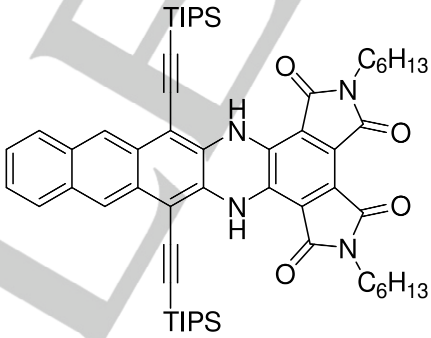

GP was applied to 1,4-bis((triisopropylsilyl)ethynyl)anthracene-2,3-diamine **8** (62.8 mg, 110 µmol, 1.00 eq.) and **MDI-Cl₂** (50.0 mg, 110 µmol, 1.00 eq), using cesium carbonate (108 mg, 331 µmol, 3.00 eq.) and PdRuPhos (G1) (4.50 mg, 5.51 µmol, 5 mol%). The resulting crude product was purified by column chromatography (SiO_2, PE/DCM 1:1) yielding **3-H₂** as a dark-red solid (61.4 mg, 64.7 µmol, 59%). R_f (DCM:PE) = 0.33, ^{1}H NMR (600 MHz, $CDCl_3$) δ = 9.29 - 9.24 (m, 2H), 8.52 - 8.48 (m, 2H), 7.86 - 7.82 (m, 2H), 7.45 - 7.42 (m, 2H), 3.68 - 3.65 (m, 4H), 1.70 - 1.65 (m, 4H), 1.43 - 1.36 (m, 6H), 1.33 - 1.27 (m, 48H), 0.91 - 0.88 (m, 6H). ^{13}C NMR (151 MHz, $CDCl_3$) δ = 167.9, 164.0, 133.3, 132.2, 131.0, 128.4, 128.0, 126.0, 124.2, 120.1, 111.9, 107.4, 102.3, 98.2, 38.1, 31.3, 28.1, 26.4, 22.4, 18.9, 14.0, 11.3. HRMS (MALDI+, DCTB): m/z calcd. for $C_{58}H_{76}N_4O_4Si_2$: $[M]^{\cdot+}$ 948.5405, found: 948.5400, correct isotope distribution. IR

WILEY-VCH

(ATR) ṽ [cm^{-1}] = 3314, 2938, 2890, 2861, 2359, 2341, 2138, 1766, 1697, 1577, 1525, 1489, 1455, 1400, 1375, 1361, 1346, 1112, 994, 879, 742, 673, 638, 620, 576, 468. M.p. = 269 °C.

2,5-Dihexyl-8,15-bis((triisopropylsilyl)ethynyl)naphtho[2,3-*i*]dipyrrolo[3,4-*a*:3',4'-*c*]phenazine-1,3,4,6(2*H*,5*H*)-tetraone (3a)

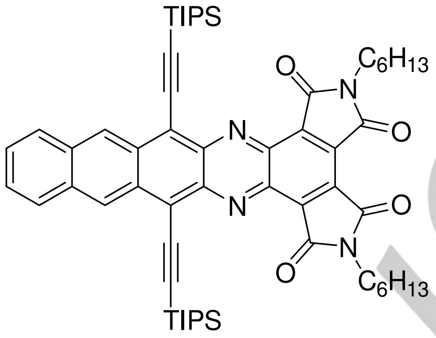

3-H$_2$ (45.0 mg, 47.4 µmol, 1.00 eq) was dissolved in methylene chloride and an excess of manganese dioxide (>100 eq.) was added. The reaction mixture was stirred at 50 °C for 1 d until TLC showed full consumption of the dihydro species. The mixture was filtered through a PTFE filter and the solvent was removed under reduced pressure to yield **3a** as a brown solid (42.3 mg, 44.7 µmol, 94%). ^{1}H NMR (600 MHz, $CDCl_3$) δ = 9.56 - 9.52 (m, 2H), 8.03 - 8.00 (m, 2H), 7.57 - 7.53 (m, 2H), 3.91 - 3.82 (m, 4H), 1.83 - 1.76 (m, 4H), 1.53 - 1.46 (m, 6H), 1.43 - 1.32 (m, 48H), 0.92 - 0.89 (m, 6H). ^{13}C NMR (151 MHz, $CDCl_3$) δ = 164.8, 164.8, 141.2, 139.9, 134.7, 134.6, 134.2, 130.3, 128.8, 128.0, 127.7, 123.0, 113.4, 102.9, 38.7, 31.4, 28.3, 26.5, 22.4, 19.0, 14.1, 11. HRMS (MALDI-, DCTB): m/z calcd. for $C_{58}H_{74}N_4O_4Si_2$: $[M]^{\cdot+}$ 946.5249, found: 946.5251, correct isotope distribution. IR (ATR) ṽ [cm^{-1}] = 2925, 2861, 1779, 1720, 1456, 1436, 1396, 1366, 1011, 694, 673, 576, 458. M.p. = 150 °C dec.

2,5-Dihexyl-8,12-bis((triisopropylsilyl)ethynyl)-7,13-dihydrodipyrrolo[3,4-*a*:3',4'-*c*][1,2,5]thiadiazolo[3,4-*i*]phenazine-1,3,4,6(2*H*,5*H*)-tetraone (4-H$_2$)

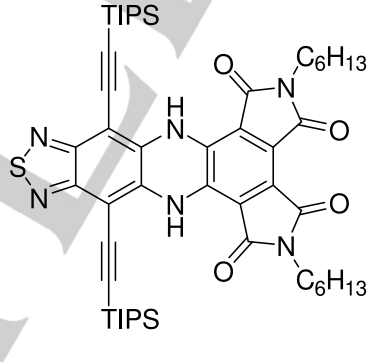

GP was applied to 4,7-bis((triisopropylsilyl)ethynyl)benzo[*c*][1,2,5]-thiadiazole-5,6-diamine **9** (58.1 mg, 110 µmol, 1.00 eq.) and **MDI-Cl$_2$** (50.0 mg, 110 µmol, 1.00 eq), using cesium carbonate (108 mg, 331 µmol, 3.00 eq.) and PdRuPhos (G1) (4.50 mg, 5.51 µmol, 5 mol%). The resulting crude product was purified by column chromatography (SiO_2, PE/DCM 1:1) yielding **4-H$_2$** as an orange-red solid (87.0 mg, 95.9 µmol, 87%). R_f (DCM:PE) = 0.29, ^{1}H NMR (600 MHz, $CDCl_3$) δ = 9.30 - 9.26 (m, 2H), 3.66 (t, J = 7.2 Hz, 4H), 1.70 - 1.62 (m, 4H), 1.36 - 1.28 (m, 18H), 1.25 - 1.19 (m, 36H), 0.90 - 0.86 (m, 6H). ^{13}C NMR (151 MHz, $CDCl_3$) δ = 167.6, 163.7, 152.0, 134.9, 132.4, 120.8, 113.1, 108.2, 97.7, 95.9, 38.7, 31.6, 28.1, 26.4, 22.4, 18.7, 14.0, 11.2. HRMS (MALDI+, DCTB): m/z calcd. for $C_{50}H_{70}N_6O_4SSi_2$: $[M+H]^+$ 907.4791, found:

907.4771, correct isotope distribution. IR (ATR) ṽ [cm^{-1}] = 3306, 2941, 2863, 2359, 2342, 1765, 1698, 1567, 1513, 1489, 1455, 1431, 1398, 1352, 1310, 1275, 1260, 1111, 1017, 880, 818, 741, 669, 653, 630, 574, 500, 465. M.p. = 151 °C.

2,5-Dihexyl-8,12-bis((triisopropylsilyl)ethynyl)dipyrrolo[3,4-*a*:3',4'-*c*][1,2,5]thiadiazolo[3,4-*i*]phenazine-1,3,4,6(2*H*,5*H*)-tetraone (4a)

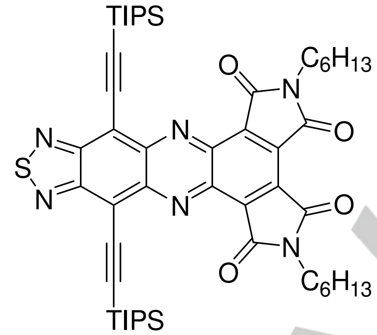

4-H₂ (45.0 mg, 49.6 µmol, 1.00 eq) was dissolved in chloroform (10 mL) and an excess of PbO_2 (>100 eq.) was added. The reaction was then stirred at 65 °C for 2 d until NMR spectroscopy showed full consumption of the dihydro species. The reaction mixture was filtered through a PTFE filter to yield **4a** as a dark-green solid (41.3 mg, 45.6 µmol, 92%). ^{1}H NMR (600 MHz, $CDCl_3$) δ = 3.85 (t, *J* = 7.2 Hz, 4H), 1.79 (quin, *J* = 7.3 Hz, 4H), 1.46 - 1.30 (m, 54H), 0.90 (br t, *J* = 7.2 Hz, 6H). ^{13}C NMR (151 MHz, $CDCl_3$) δ = 164.8, 164.7, 157.1, 143.0, 140.7, 135.1, 131.6, 116.8, 101.8, 39.1, 31.6, 28.6, 26.7, 22.7, 19.2, 14.4, 11.9. HRMS (MALDI-, DCTB): m/z calcd. for $C_{50}H_{68}N_6O_4SSi_2$: $[M]^{\cdot+}$ 904.4561, found: 904.4560, correct isotope distribution. IR (ATR) ṽ [cm^{-1}] = 2922, 2863, 1781, 1773, 1721, 1714, 1462, 1444, 1398, 1364, 1194, 1062, 1014, 996, 921, 880, 801, 734, 670, 656, 579, 503, 445, 406. M.p. = 206 °C.

2,5-Dihexyl-8,15-bis((triisopropylsilyl)ethynyl)-7,16-dihydrodipyrrolo[3,4-*a*:3',4'-*c*]quinoxalino[2,3-*i*]phenazine-1,3,4,6(2*H*,5*H*)-tetraone (5-H₂)

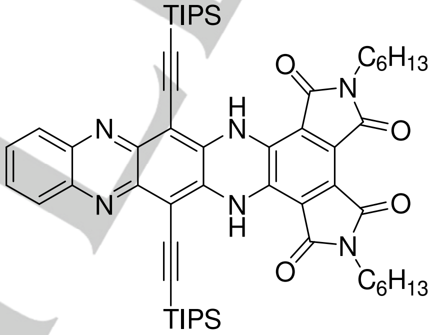

In a dry Schlenk tube bis((triisopropylsilyl)ethynyl)phenazine-2,3-diamine **10** (75.6 mg, 132 µmol, 1.20 eq.), **MDI-Cl₂** (50.0 mg, 110 µmol, 1.00 eq), $Pd_2(dba)_3$ (5.05 mg, 5.51 µmol, 5 mol%) and RuPhos (5.15 mg, 11.0 µmol, 10 mol%) were placed under argon atmosphere. Then degassed Hünig's base (2 mL) was added and the reaction was stirred at 120 °C for 16 h. The reaction mixture was diluted with methylene chloride and water was added. The phases where separated and the aqueous layer was extracted with methylene chloride (3x10 mL). The combined organic layers were dried over $MgSO_4$, filtered through a paper filter and the solvent was removed under reduced pressure. The crude product was purified by column chromatography (SiO_2, PE/DCM 1:1) to yield **5-H₂** as a pink-red solid (63.3 mg, 66.5 µmol,

60%). R_f (DCM:PE) = 0.31, ^{1}H NMR (301 MHz, $CDCl_3$) δ = 9.48 (s, 2H), 8.05 - 7.95 (m, 2H), 7.72 - 7.63 (m, 2H), 3.67 (t, J = 7.0 Hz, 4H), 1.76 - 1.61 (m, 4H), 1.44 - 1.17 (m, 54H), 0.95 - 0.82 (m, 6H). ^{13}C NMR (151 MHz, $CDCl_3$) δ = 167.8, 164.0, 143.0, 135.7, 132.4, 130.0, 129.6, 128.1, 121.0, 113.4, 108.8, 103.4, 97.3, 38.4, 31.4, 29.9, 26.5, 22.6, 19.0, 14.2, 11.5. HRMS (MALDI+, DCTB): m/z calcd. for $C_{56}H_{75}N_6O_4Si_2$: $[M+H]^+$ 951.5383, found: 951.5391, correct isotope distribution. IR (ATR) ṽ [cm^{-1}] = 3303, 2940, 2924, 2861, 2359, 2341, 1760, 1698, 1577, 1518, 1350, 1254, 1215, 1095, 1019, 881, 749, 722, 697, 660, 628, 590, 470. M.p. = 261 °C.

2,5-Dihexyl-8,15-bis((triisopropylsilyl)ethynyl)dipyrrolo[3,4-*a*:3',4'-*c*]quinoxalino[2,3-*i*]phenazine-1,3,4,6(2*H*,5*H*)-tetraone (5a)

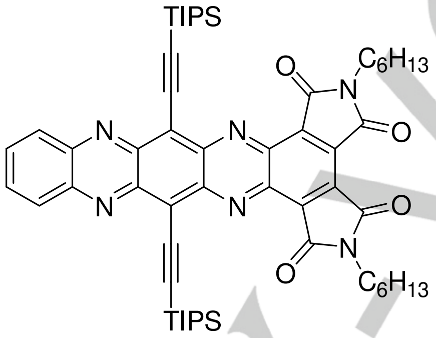

5-H₂ (22.0 mg, 23.1 µmol, 1.00 eq.) was dissolved in methylene chloride (1 mL) in a glove box. Then an excess of PbO_2 (>100 eq.) was added. The reaction was stirred at rt for 3 d. The reaction mixture was filtered through a PTFE filter to yield **5a** as a brown solid (20.2 mg, 21.3 µmol, 92%) ^{1}H NMR (600 MHz, $CDCl_3$) δ = 8.25 - 8.16 (m, 2H), 7.97 - 7.89 (m, 2H), 3.85 (t, J = 7.2 Hz, 4H), 1.80 (quin, J = 7.2 Hz, 4H), 1.48 - 1.33 (m, 55H), 0.94 - 0.90 (m, 6H). ^{13}C NMR (151 MHz, $CDCl_3$) δ = 165.0, 164.8, 146.8, 145.1, 143.2, 140.8, 135.2, 133.9, 131.7, 130.7, 125.1, 116.7, 103.1, 38.9, 31.6, 28.6, 26.7, 22.8, 19.0, 14.1, 12.0. HRMS (MALDI-, DCTB): m/z calcd. for $C_{54}H_{74}N_6O_4Si_2$: $[M]^{\cdot-}$ 948.5159, found: 948.5152, correct isotope distribution. IR (ATR) ṽ [cm^{-1}] = 2926, 2861, 1778, 1722, 1523, 1442, 1388, 1361, 1127, 1062, 1016, 880, 765, 745, 736, 673, 594, 447. M.p. = 185 °C dec.

WILEY-VCH

2,5,13,16-Tetrahexyl-9,20-bis([tri(propan-2-yl)silyl]ethynyl)-7,11,18,22-tetrahydrotetrapyrrolo[3,4-*h*:3,4-*h'*:3,4-*j*:3,4-*j'*]benzo[1,2-*b*:4,5-*b'*]diphenazine-1,3,4,6,12,14,15,17(2*H*,5*H*,13*H*,16*H*)-octone (12-H4)

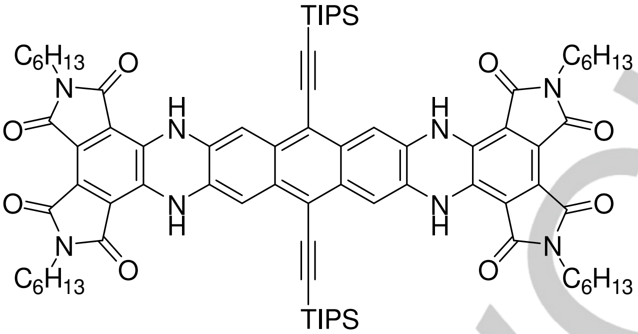

GP was applied to 9,10-bis((triisopropylsilyl)ethynyl)anthracene-2,3,6,7-tetraaminiumchloride **11** (100 mg, 134 µmol, 1.00 eq.) and **MDI-Cl2** (137 mg, 302 µmol, 2.25 eq), using cesium carbonate (875 mg, 2.69 mmol, 20.0 eq.) and PdRuPhos (G1) (11.0 mg, 13.4 µmol, 10 mol%). The resulting crude product was purified via column chromatography (SiO_2, PE/EE 5:1) then washed with methanol and PE and subsequently purified via preparative GPC ($CHCl_3$) yielding **12-H4** as a dark purple solid (59.3 mg, 42.9 µmol, 32%). R_f (DCM:PE) = 0. ^1H NMR (600 MHz, CD_2Cl_2) δ = 8.46 (s, 4H), 7.36 - 7.34 (m, 4H), 3.63 (br t, *J* = 7.2 Hz, 8H), 1.68 - 1.63 (m, 8H), 1.34 - 1.29 (m, 66H), 0.90 (br t, *J* = 6.9 Hz, 12H). ^{13}C NMR (151 MHz, CD_2Cl_2) δ = 168.8, 164.0, 134.4, 131.3, 129.6, 120.00, 114.7, 111.4, 108.9, 102.9, 31.7, 28.7, 26.8, 22.8, 19.0, 14.1, 11.9. HRMS (MALDI+, DCTB): m/z calcd. for $C_{80}H_{103}N_8O_8Si_2$: $[M+H]^+$ 1359.7432, found: 1359.7429, correct isotope distribution. IR (ATR) $\tilde{\nu}$ [cm^{-1}] = 3345, 2924, 2854, 2362, 2341, 2331, 1769, 1698, 1685, 1570, 1498, 1465, 1364, 1278, 778, 758, 733, 691, 678, 550. M.p. = >330 °C.

Butterfly-Dimer (13)

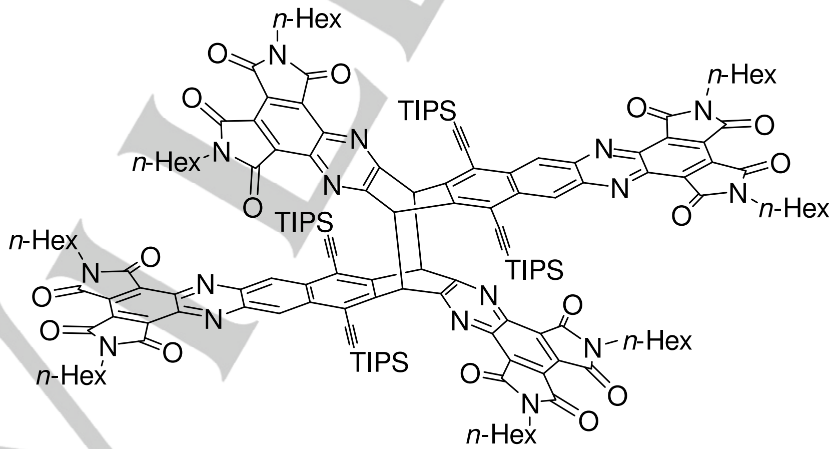

12-H4 (2.00 mg, 1.47 µmol, 1.00 eq) was dissolved in methylene chloride (500 µL) in a glove box. Then an excess of PbO_2 (>100 eq.) was added. The reaction was stirred at rt for 1 d. The reaction mixture was filtered through a PTFE filter to yield **13** as a green solid (2.60 mg, 960 nmol, 65%). ^1H NMR (600 MHz, CD_2Cl_2) δ = 9.51 (s, 4H), 6.64 - 6.47 (m, 4H), 3.81 (br t, J = 7.3 Hz, 8H), 3.70 - 3.61 (m, 8H), 1.80 - 1.73 (m, 8H), 1.70 - 1.46 (m, 126H), 1.45 - 1.39 (m, 14H), 0.91 - 0.87 (m, 24H). ^{13}C NMR (151 MHz, CD_2Cl_2) δ = 165.7, 165.0, 164.3, 164.1, 159.6,

142.3, 141.3, 139.6, 139.0, 135.2, 135.1, 133.6, 131.1, 131.0, 129.8, 122.3, 110.5, 101.1, 57.8, 39.2, 39.0, 32.0, 31.8, 29.0, 28.8, 27.1, 27.0, 23.1, 23.0, 19.6, 19.5, 14.4, 12.3. HRMS (MALDI+, DCTB): m/z calcd. for $C_{160}H_{196}N_{16}O_{16}Si_4$: $[M/2]^+$ 1354.7046, found: 1354.7027, correct isotope distribution. IR (ATR) ṽ [cm^{-1}] = 2955, 2922, 2853, 2359, .2341, 1773, 1725, 1458, 1391, 1376, 1365, 1259, 1083, 1014, 880, 702, 679, 670, 661, 403. One constitutional isomer selectively formed, but as we did not obtain a single crystal suitable for crystal structure analysis, we cannot distinguish between the head-to-tail and the head-to-head dimer (see Figure S1).

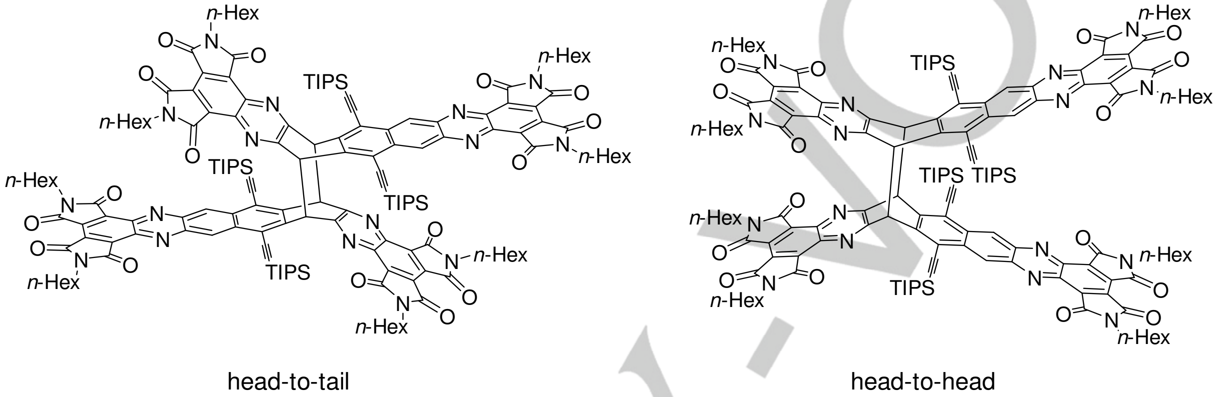

Figure S1. Two possible constitutional isomers of the butterfly dimer **13**.

WILEY-VCH

3) Cyclic Voltammetry

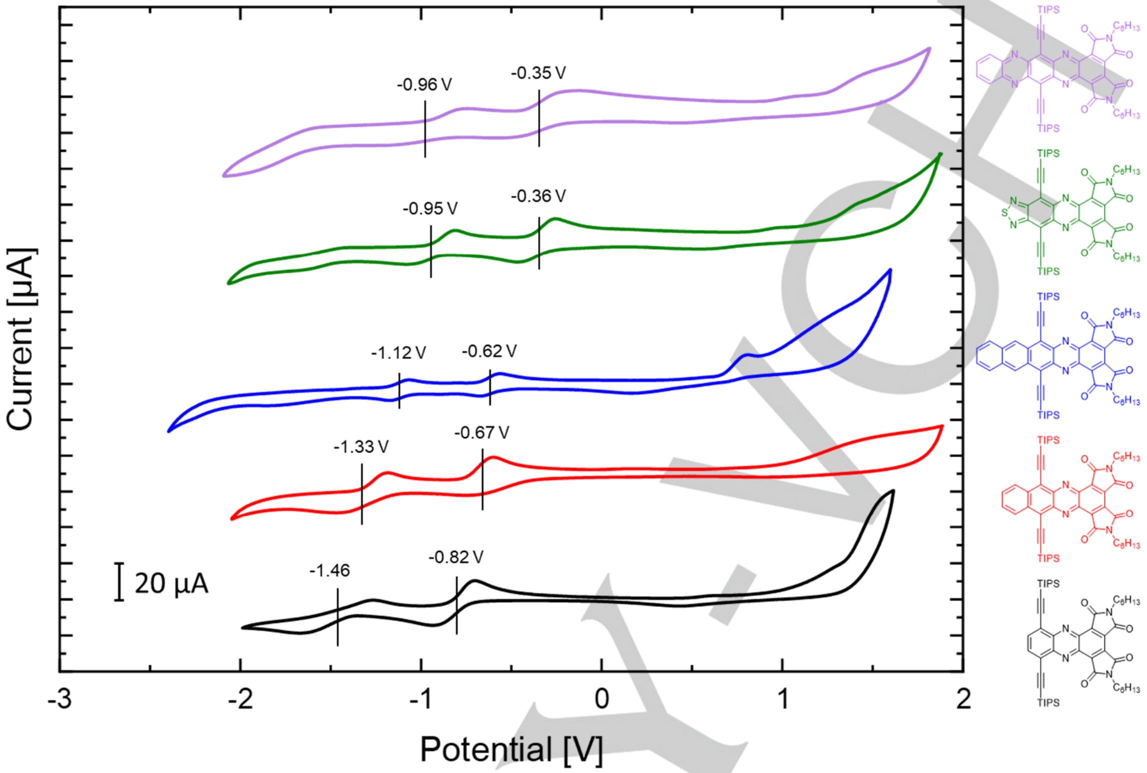

Figure S2. Cyclic voltammograms of **1a** (black), **2a** (red), **3a** (blue), **4a** (green), **5a** (purple) in CH_2Cl_2 using Bu_4NPF_6 as electrolyte and Fc/Fc$^+$ as internal standard at 0.2 Vs^{-1}.

Table S1. Optical, electrochemical and quantum-chemical data of the azaacenebisimides and their parent compounds.

Compound	$E_{(0/-)}$ [V][a]	E_A [eV][b]	$E_{LUMO, DFT}$ [eV][c]	IP [eV][d]	$E_{HOMO, DFT}$ [eV][c]	gap_{DFT} [eV]	$\lambda_{max, abs}$ [nm]	opt. gap [eV][e]
**1a**	-0.82	-4.28	-3.92	-6.32	-6.27	2.35	550	2.05
**1b**[f]	-1.68	-3.42	-3.08	-6.12	-5.97	2.88	440	2.67
**2a**	-0.67	-4.43	-4.04	-5.99	-5.83	1.79	733	1.56
**2b**[f]	-1.23	-3.87	-3.35	-5.99	-5.54	2.20	570	2.09
**3a**	-0.62	-4.48	-4.10	-5.87	-5.50	1.40	908	1.31
**3b**[f]	-1.05	-4.15	-3.50	-5.80	-5.25	1.75	692	1.74
**4a**	-0.36	-4.74	-4.37	-6.29	-6.03	1.66	766	1.55
**4b**[g]	-0.83	-4.27	-3.85	-6.13	-5.77	1.82	642	1.86
**5a**	-0.35	-4.75	-4.30	-6.19	-5.80	1.50	813	1.44
**5b**[h]	-0.79	-4.07	-3.43	-5.89	-5.29	1.86	680	1.82

[a]First reduction potentials measured by cyclic voltammetry (CV) in dichloromethane with Bu_4NPF_6 as the electrolyte against Fc/Fc$^+$ as an internal standard (–5.10 eV) at 0.2 V s^{-1}. [b]Calculated from CV measurements (EA = -5.10 eV - $E^{(0/-)}$)[S10] [c]Obtained from quantum-chemical calculations with DFT/B3LYP/def2-TZVP [d]IP = E_A – Opt.Gap [e]Calculated from $\lambda_{onset, abs}$. [f]CV data and quantum-chemical calculations taken from reference [S11] and adjusted for Fc/Fc$^+$ = -5.10 eV [g] CV data and quantum-chemical calculations taken from reference [S12] [h] CV data and quantum-chemical calculations taken from reference [S13] and adjusted for Fc/Fc$^+$ = -5.10 eV.

4) Optical Spectroscopy

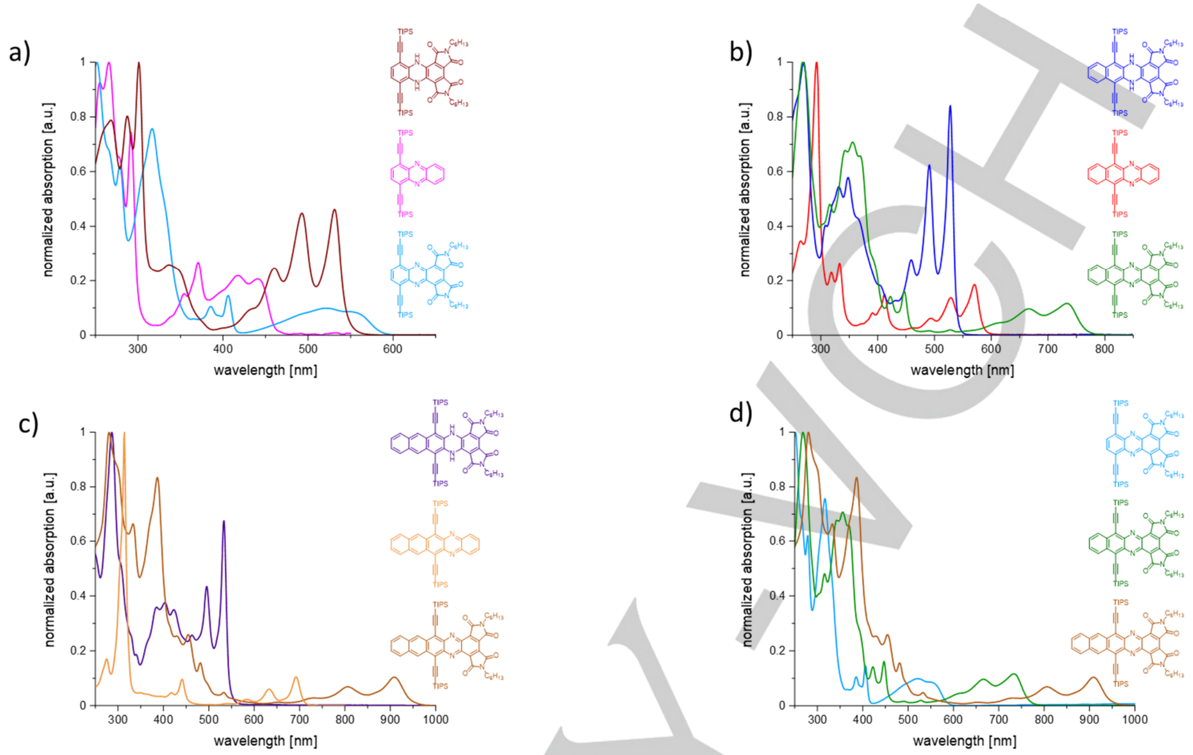

Figure S3. Absorption spectra of the a) diazaanthracenes, b) diazatetracenes, c) diazapentacenes and their respective dihydro-species. The absorption spectra of the diazaacenebisimides in relation to each other are shown in d).

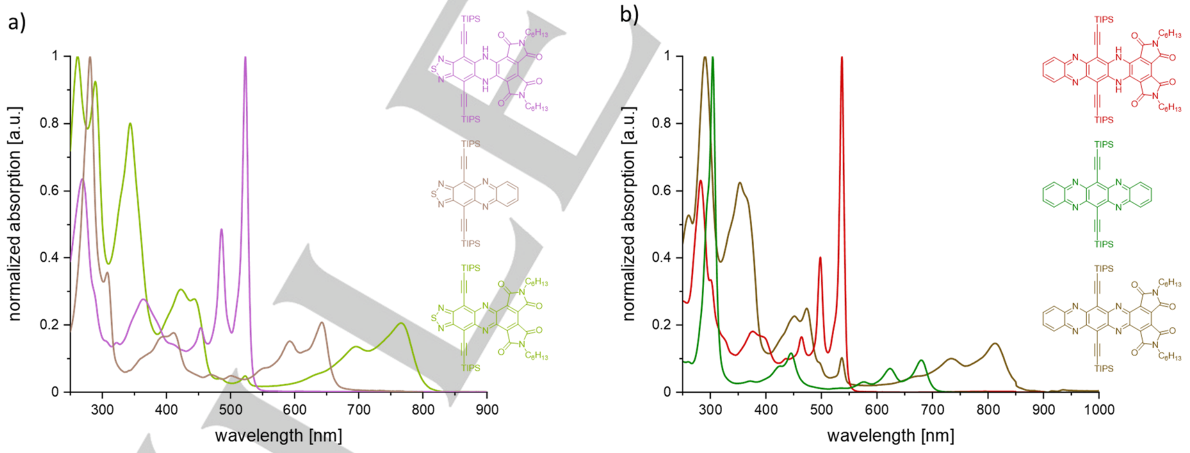

Figure S4. Absorption spectra of the a) thiadiazolacenes and b) tetraazapentacenes and their respective dihydro species.

WILEY-VCH

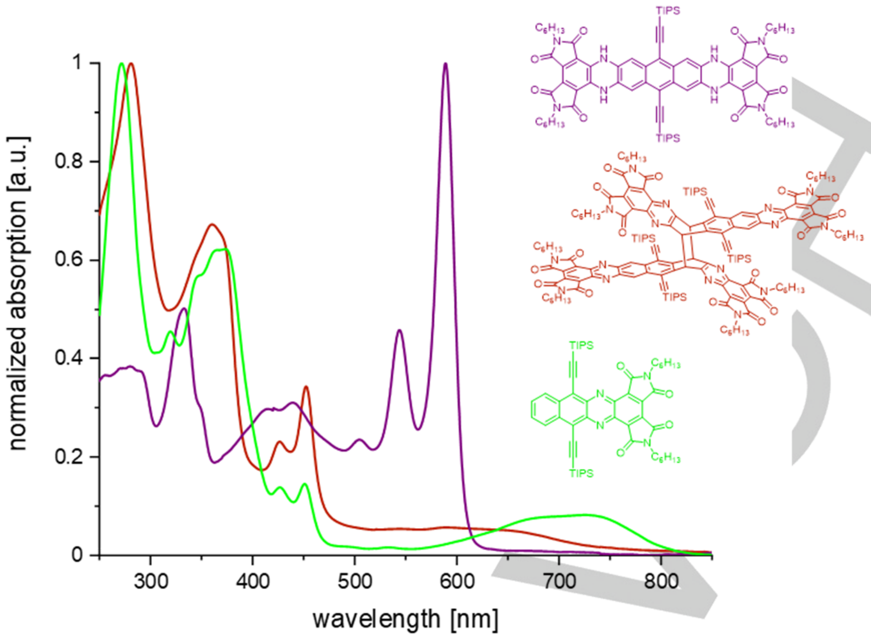

Figure S5. Absorption spectra of **2a**, **12-H$_4$** and **13**.

WILEY-VCH

5) Geometry and Energy computation

Geometry optimizations were performed using the B3LYP functional and def2SVP basis set. At this geometry, the absolute energy and FMO energies were determined by a single-point approach at the B3LYP/def2TZVP level.

Energy and cartesian coordinates for **1a**:

Total Energy = -2575.53647331 Hartree

No imaginary frequencies.

Table S2: Cartesian coordinates 1a.

Tag	Symbol	X	Y	Z
1	C	0.6910653	-5.2798054	0.1287146
2	C	-0.6931087	-5.2791317	-0.1377764
3	C	-1.4146702	-4.1128656	-0.2743909
4	C	-0.7109664	-2.8598083	-0.1361797
5	C	0.7093228	-2.8605142	0.1355012
6	C	1.412858	-4.1141696	0.2691364
7	N	-1.3824399	-1.7216624	-0.2616131
8	C	-0.7106923	-0.5770617	-0.1319007
9	C	0.7103041	-0.5778145	0.1370352
10	N	1.3812957	-1.7230869	0.2643715
11	C	-1.3662919	0.6791263	-0.2543101
12	C	-0.6909994	1.8698034	-0.1275511
13	C	0.6935143	1.869033	0.1329435
14	C	1.3672217	0.6775588	0.260407
15	C	1.6794044	2.9914907	0.3218598
16	N	2.9068864	2.3702997	0.5512149
17	C	2.8154535	0.9778197	0.5340157
18	C	-2.8142222	0.9812382	-0.5274903
19	N	-2.9034421	2.3738156	-0.5473509
20	C	-1.6751581	2.9934679	-0.3181023
21	O	-3.7387018	0.2298584	-0.7037104
22	O	-1.493649	4.1822031	-0.2948866
23	O	1.4996739	4.1804697	0.2973429
24	O	3.7384644	0.2252671	0.7129991
25	C	2.8030407	-4.1214821	0.536656
26	C	-2.8045941	-4.1196381	-0.5433733
27	C	-3.9949944	-4.1130358	-0.7751292
28	C	3.9938364	-4.1138265	0.7663205
29	C	-9.0593673	5.5855131	2.5332758
30	C	-8.283559	5.265147	1.2571004
31	C	-6.9846818	4.5012573	1.5158737
32	C	-6.2019569	4.1769353	0.2432096
33	C	-4.9056857	3.4125363	0.5101348

34	C	-4.1433692	3.1030985	-0.7785944
35	C	9.0590262	5.5745204	-2.5439965
36	C	8.2862929	5.2549129	-1.2657629
37	C	6.9853491	4.4934424	-1.5212516
38	C	6.2056964	4.1696536	-0.2465636
39	C	4.9074688	3.4075346	-0.5104746
40	C	4.1481843	3.0980254	0.7800416
41	Si	5.7965477	-3.9527731	1.1190762
42	C	6.6354223	-5.5828521	0.7068987
43	C	5.9945583	-3.5522638	2.9433046
44	C	6.4634606	-2.5604767	0.0545651
45	Si	-5.7990497	-3.9571675	-1.1234386
46	C	-6.4053575	-5.5708666	-1.8713302
47	C	-6.0285382	-2.5299628	-2.3176548
48	C	-6.6741589	-3.6156133	0.5030824
49	H	1.2051071	-6.2265085	0.2261983
50	H	-1.2073561	-6.2253509	-0.2388068
51	H	-9.9798846	6.1300556	2.3140766
52	H	-9.3333397	4.672932	3.068649
53	H	-8.4643695	6.2002602	3.2133198
54	H	-8.0543721	6.1953557	0.7264799
55	H	-8.9172019	4.6797266	0.5823481
56	H	-7.213577	3.5700994	2.046766
57	H	-6.3501738	5.0868891	2.1908059
58	H	-5.9713422	5.1084455	-0.2859476
59	H	-6.8365241	3.5912062	-0.4316034
60	H	-5.1286004	2.473516	1.0249179
61	H	-4.2605363	3.997497	1.1718181
62	H	-3.8784995	4.0261972	-1.2951042
63	H	-4.7527003	2.4918707	-1.4449724
64	H	9.9811529	6.1172958	-2.3271791
65	H	9.3297973	4.6616971	-3.0805782
66	H	8.4631887	6.1906671	-3.2220383
67	H	8.0602954	6.1852693	-0.7340241
68	H	8.9208186	4.6679986	-0.5931416
69	H	7.2110903	3.5621991	-2.0533058
70	H	6.3499188	5.0805972	-2.193993
71	H	5.9781225	5.1011983	0.2838605
72	H	6.8412062	3.5823677	0.4260037
73	H	5.1275259	2.4686924	-1.0267789
74	H	4.2613736	3.9941273	-1.1697782
75	H	3.8858919	4.0210067	1.298091
76	H	4.7583922	2.4852674	1.4442164
77	H	6.4991778	-5.8422758	-0.3450696
78	H	7.7096294	-5.5244484	0.900847

WILEY

79	H	6.2308936	-6.3997791	1.3082465
80	H	5.4862553	-2.6187869	3.1921877
81	H	7.0511471	-3.441152	3.2010774
82	H	5.5764525	-4.341589	3.5713214
83	H	6.3642927	-2.7947263	-1.0074493
84	H	5.9154504	-1.6359809	0.2469715
85	H	7.5221615	-2.3848811	0.2634583
86	H	-6.2508013	-6.4077858	-1.1870045
87	H	-7.473643	-5.515112	-2.0963049
88	H	-5.8792763	-5.7971624	-2.8011276
89	H	-5.5987497	-1.6139889	-1.9074799
90	H	-7.0896369	-2.356096	-2.515573
91	H	-5.5372272	-2.732562	-3.2716751
92	H	-6.5159036	-4.4261531	1.2173914
93	H	-6.306655	-2.6921447	0.9545981
94	H	-7.7509941	-3.5102886	0.3465776

Energy and cartesian coordinates for **2a**:

Total Energy = -2729.22455736 Hartree

No imaginary frequencies.

Table S3: Cartesian coordinates 2a.

Tag	Symbol	X	Y	Z
1	C	0.722562	-4.8785541	0.4042501
2	C	-0.7217658	-4.8786708	0.4042337
3	C	-1.4398909	-3.6900192	0.171278
4	C	-0.7233643	-2.480537	-0.0640191
5	C	0.7237921	-2.4804201	-0.0640196
6	C	1.4405061	-3.6897888	0.171304
7	C	-2.8530206	-3.6627077	0.1637051
8	C	2.8536342	-3.6622765	0.1637779
9	C	1.4008074	-6.1104163	0.6455988
10	C	0.7112402	-7.2622702	0.8712648
11	C	-0.7100756	-7.262386	0.8712403
12	C	-1.3998212	-6.1106432	0.6455542
13	C	4.0666757	-3.6189471	0.152335
14	C	-4.0660634	-3.6194518	0.1521663
15	N	-1.4073295	-1.3528443	-0.2832274
16	N	1.4075696	-1.3526194	-0.2832777
17	C	0.7266841	-0.2366105	-0.4996456
18	C	-0.7266323	-0.2367242	-0.499599
19	C	-1.3930177	1.0013808	-0.7386378
20	C	-0.7066349	2.16788	-0.9646453
21	C	0.7062615	2.1679866	-0.9647261

22	C	1.3928518	1.0015963	-0.7387778
23	C	1.707049	3.2720922	-1.1789542
24	N	2.9576159	2.6658316	-1.0599921
25	C	2.8662481	1.2987543	-0.7961191
26	C	-2.8664679	1.2983092	-0.7957874
27	N	-2.9580796	2.6653531	-1.0597468
28	C	-1.7076202	3.2718311	-1.1787473
29	O	-1.5242185	4.4378545	-1.4103753
30	O	-3.8080667	0.5598129	-0.6577914
31	O	1.5234367	4.4381031	-1.4104797
32	O	3.8079792	0.5604277	-0.6581219
33	Si	5.8952976	-3.3904124	0.0976353
34	C	6.7115282	-4.9025702	0.8594506
35	C	6.3072762	-1.8398757	1.0681156
36	C	6.4002809	-3.2053533	-1.7016297
37	Si	-5.8946762	-3.3908972	0.0972912
38	C	-6.3069008	-1.8414166	1.0693661
39	C	-6.7110601	-4.9038965	0.8572643
40	C	-6.3992297	-3.2038083	-1.7018784
41	C	8.4265097	6.623893	2.4231791
42	C	7.9020607	6.0418191	1.1119881
43	C	6.5790395	5.2920947	1.2705552
44	C	6.0475684	4.7063144	-0.0378095
45	C	4.7252996	3.9581202	0.1285933
46	C	4.2179267	3.3838673	-1.1944755
47	C	-8.4276696	6.6224649	2.4234079
48	C	-7.9031235	6.0404682	1.1122214
49	C	-6.5799588	5.2909971	1.2707881
50	C	-6.0483915	4.7052928	-0.0375717
51	C	-4.7259811	3.9573489	0.1288306
52	C	-4.2185195	3.3831618	-1.1942326
53	H	2.4818503	-6.1041923	0.6446891
54	H	1.2452217	-8.1863501	1.0525005
55	H	-1.2439128	-8.1865531	1.0524559
56	H	-2.480865	-6.104593	0.6446088
57	H	6.4572828	-5.8108936	0.3088792
58	H	7.7997713	-4.7998342	0.8492683
59	H	6.3989691	-5.0403448	1.8967266
60	H	5.7754818	-0.9808567	0.6544426
61	H	7.3802711	-1.6331437	1.0313728
62	H	6.0197163	-1.9422868	2.1166049
63	H	6.1328761	-4.0898885	-2.2832758
64	H	5.9071785	-2.3425483	-2.1532501
65	H	7.4806586	-3.0606792	-1.7858361
66	H	-5.7750304	-0.9819573	0.6567035

67	H	-7.3798868	-1.6346486	1.0325791
68	H	-6.0195938	-1.9449276	2.1178157
69	H	-6.4566944	-5.8116089	0.3057421
70	H	-7.7993017	-4.8011577	0.8469722
71	H	-6.3987147	-5.0428145	1.8944525
72	H	-5.9060384	-2.3404575	-2.1523577
73	H	-6.1316423	-4.0876564	-2.2844831
74	H	-7.4795918	-3.0590773	-1.7861877
75	H	9.3705951	7.1519676	2.2755571
76	H	7.7141791	7.3321771	2.8536733
77	H	8.5980909	5.8383688	3.1634535
78	H	8.6511454	5.364436	0.6882455
79	H	7.7739473	6.8471586	0.3809447
80	H	5.8289471	5.96973	1.6939834
81	H	6.7066692	4.4859507	2.0020745
82	H	6.7976728	4.0283484	-0.4607539
83	H	5.9184225	5.5129799	-0.768258
84	H	3.9669761	4.6326894	0.5361555
85	H	4.84926	3.1416881	0.8459047
86	H	4.9420149	2.6811458	-1.6078669
87	H	4.0541272	4.1831352	-1.9179886
88	H	-9.3718564	7.1503583	2.2757864
89	H	-8.5990947	5.8369208	3.1636972
90	H	-7.715469	7.3308912	2.853883
91	H	-7.7751702	6.8458195	0.381163
92	H	-8.6520841	5.3629359	0.6884981
93	H	-6.7074281	4.4848425	2.0023237
94	H	-5.82999	5.968782	1.6941956
95	H	-5.919405	5.5119689	-0.7680365
96	H	-6.7983734	4.0271783	-0.4604957
97	H	-4.8497805	3.1409095	0.8461612
98	H	-3.967778	4.6320681	0.5363686
99	H	-4.0548688	4.182445	-1.9177622
100	H	-4.942489	2.6803056	-1.6076032

Energy and cartesian coordinates for **3a**:

Total Energy = -2882.91176840 Hartree

No imaginary frequencies.

Table S4: Cartesian coordinates 3a.

Tag	Symbol	X	Y	Z
1	C	-0.7048999	9.1602884	0.1322162
2	C	0.6985044	9.1607783	-0.132186
3	C	1.3797354	7.9910001	-0.2605806

4	C	0.7080929	6.73462	-0.1340459
5	C	-0.7127956	6.7341239	0.1340733
6	C	-1.3853145	7.9900348	0.2606091
7	C	1.3729525	5.5268745	-0.2597616
8	C	0.7115604	4.2918717	-0.134953
9	C	-0.7145584	4.2913725	0.1349735
10	C	-1.3768122	5.5259138	0.2597858
11	C	1.4161938	3.0681753	-0.2684171
12	C	0.7119694	1.8443828	-0.1345562
13	C	-0.7132603	1.843882	0.1345682
14	C	-1.41834	3.0671834	0.2684284
15	N	1.3840554	0.6905807	-0.2601754
16	C	0.7162982	-0.4427055	-0.1339302
17	C	-0.7159748	-0.4432107	0.1339524
18	N	-1.3845324	0.6896057	0.2601933
19	C	1.3711246	-1.7059619	-0.2550272
20	C	0.6966311	-2.8929087	-0.1294617
21	C	-0.6945678	-2.8934018	0.1294975
22	C	-1.369904	-1.7069323	0.2550584
23	C	-1.6763072	-4.0190331	0.3169453
24	N	-2.9070869	-3.4027387	0.5431501
25	C	-2.8182837	-2.0104576	0.5252109
26	C	2.8197205	-2.0084579	-0.525177
27	N	2.9095121	-3.4006759	-0.5431117
28	C	1.6791706	-4.0178429	-0.3169021
29	O	1.496286	-5.2066017	-0.2941492
30	O	-1.4925793	-5.2076616	0.2941921
31	O	-3.7438708	-1.2598668	0.7013835
32	O	3.7447756	-1.2572089	-0.7013388
33	C	-2.8049976	3.0380801	0.5323411
34	C	2.8028705	3.0400313	-0.5323297
35	C	3.9949733	3.0006708	-0.7602841
36	C	-3.9970775	2.9979486	0.7602836
37	C	-9.0392647	-6.6416604	-2.5564548
38	C	-8.2706744	-6.3147196	-1.277561
39	C	-6.9722598	-5.5487786	-1.5325722
40	C	-6.1967743	-5.2176498	-0.2572282
41	C	-4.9009918	-4.4511724	-0.5204584
42	C	-4.1459252	-4.13435	0.7707641
43	C	9.04393	-6.6353468	2.5565012
44	C	8.2751304	-6.3089111	1.2776042
45	C	6.9761644	-5.5439063	1.5326172
46	C	6.2004669	-5.2132872	0.2572699
47	C	4.9041358	-4.4477385	0.5205011
48	C	4.1488692	-4.1314085	-0.7707251

WILEY-VCH

49	Si	-5.7919163	2.7632956	1.1080513
50	C	-6.677328	4.3963068	0.8192599
51	C	-5.9683547	2.2268198	2.8991142
52	C	-6.4308234	1.434945	-0.0508313
53	Si	5.789903	2.7668881	-1.1081619
54	C	6.6744374	4.4005019	-0.8200967
55	C	5.966431	2.2297904	-2.8990266
56	C	6.4296792	1.4393452	0.0511704
57	H	-1.2243641	10.105077	0.2299211
58	H	1.2173092	10.1059294	-0.2298898
59	H	2.4441957	7.9898306	-0.4610328
60	H	-2.4497741	7.9881222	0.4610602
61	H	2.4361564	5.5219843	-0.4604396
62	H	-2.4400126	5.5202801	0.4604616
63	H	-9.9596698	-7.1875098	-2.3399515
64	H	-9.3125271	-5.7316961	-3.0966319
65	H	-8.4393739	-7.2575482	-3.2311534
66	H	-8.0420887	-7.2424166	-0.7422875
67	H	-8.9091159	-5.7282362	-0.6082602
68	H	-7.2005745	-4.6202112	-2.0682057
69	H	-6.3329224	-6.1355153	-2.201965
70	H	-5.9666959	-6.1465566	0.2767538
71	H	-6.8362523	-4.6308248	0.4120131
72	H	-5.1234931	-3.5149457	-1.0404855
73	H	-4.2509313	-5.037267	-1.1762871
74	H	-3.881831	-5.0547937	1.2925045
75	H	-4.7603534	-3.5220144	1.431491
76	H	9.9647288	-7.1805311	2.3399966
77	H	9.3165321	-5.725206	3.0967146
78	H	8.4444664	-7.251686	3.2311675
79	H	8.0472176	-7.2367528	0.7422945
80	H	8.9131655	-5.7219489	0.6083356
81	H	7.2038061	-4.6151944	2.0682869
82	H	6.3372333	-6.131122	2.2019777
83	H	5.9710583	-6.14234	-0.2767461
84	H	6.8395404	-4.6259868	-0.4119411
85	H	5.1259631	-3.511371	1.0405631
86	H	4.254479	-5.0343153	1.1762984
87	H	3.8854353	-5.0520217	-1.2925003
88	H	4.7628768	-3.5186175	-1.4314207
89	H	-6.5519923	4.737348	-0.2106083
90	H	-7.7489331	4.293244	1.0089051
91	H	-6.2955181	5.1771765	1.4805817
92	H	-5.4341264	1.2913166	3.0751374
93	H	-7.0201648	2.0693168	3.1523968

WILEY

94	H	-5.5667114	2.9785875	3.5815729
95	H	-7.4858491	1.2246682	0.1443847
96	H	-6.3359844	1.7421581	-1.09444
97	H	-5.8640787	0.5113696	0.0821243
98	H	6.5490809	4.7418311	0.2096742
99	H	7.7460694	4.2979802	-1.0098831
100	H	6.2920745	5.1809238	-1.4816266
101	H	5.4326533	1.293946	-3.0746032
102	H	7.0182926	2.0727133	-3.1523598
103	H	5.5643333	2.9810759	-3.5817483
104	H	7.4848627	1.2297444	-0.1439161
105	H	6.3345601	1.7468111	1.0946793
106	H	5.86359	0.5153316	-0.0815364

Energy and cartesian coordinates for **4a**:

Total Energy = -3082.06806549 Hartree

No imaginary frequencies.

Table S5: Cartesian coordinates 4a.

Tag	Symbol	X	Y	Z
1	C	-0.7110375	4.782355	0.1328502
2	C	0.7157244	4.7813478	-0.1404463
3	C	1.4489797	3.5871631	-0.2794503
4	C	0.7178336	2.3736084	-0.1369755
5	C	-0.715047	2.3746522	0.1357439
6	C	-1.4453895	3.5891728	0.274172
7	N	-1.219322	6.0305927	0.2290769
8	S	0.0034279	7.0347704	-0.0062103
9	N	1.2251925	6.0288945	-0.239355
10	N	1.3830149	1.2165666	-0.2605938
11	C	0.7154577	0.0836412	-0.1325702
12	C	-0.715142	0.0847338	0.13643
13	N	-1.3813695	1.2186409	0.2623605
14	C	1.3698742	-1.180048	-0.2540272
15	C	0.6936456	-2.3653778	-0.1284056
16	C	-0.6975649	-2.3642709	0.1316163
17	C	-1.371533	-1.1778247	0.2586024
18	C	-1.6815104	-3.4895307	0.3189986
19	N	-2.9105043	-2.8716476	0.5471368
20	C	-2.8202241	-1.4794038	0.530543
21	C	2.8182062	-1.4841417	-0.5250974
22	N	2.9054674	-2.8765446	-0.545145
23	C	1.6752832	-3.4923017	-0.3177787
24	C	-2.8265477	3.5881631	0.5381108

25	C	-4.018671	3.5710913	0.7666097
26	C	2.8297213	3.5849789	-0.5456449
27	C	4.0213453	3.5685962	-0.7767911
28	O	-1.4977564	-4.6778175	0.2942143
29	O	-3.7430544	-0.7266466	0.7087333
30	O	1.4890838	-4.680251	-0.294914
31	O	3.743051	-0.7329918	-0.6994286
32	Si	-5.8226081	3.4200327	1.1207122
33	C	-6.0150271	3.0173713	2.9452064
34	C	-6.4966378	2.0309181	0.0561966
35	C	-6.6499309	5.0553555	0.711244
36	Si	5.8277049	3.4230909	-1.1208522
37	C	6.4244678	5.0411634	-1.8641164
38	C	6.0671605	1.996947	-2.3146539
39	C	6.6948309	3.0833033	0.5104371
40	C	-9.0546821	-6.0834444	-2.5563216
41	C	-8.2837649	-5.7634757	-1.2770889
42	C	-6.9836933	-4.9999203	-1.5308079
43	C	-6.2058483	-4.675761	-0.2551056
44	C	-4.9085126	-3.9114902	-0.5172654
45	C	-4.1510078	-3.6016515	0.7741511
46	C	9.0482752	-6.1035866	2.5458823
47	C	8.2755975	-5.7810979	1.2683486
48	C	6.9780514	-5.0142162	1.5249492
49	C	6.1984532	-4.6877382	0.2509107
50	C	4.9034741	-3.9203864	0.5157122
51	C	4.1442794	-3.6087458	-0.7742627
52	H	-5.5904791	3.8039294	3.5722157
53	H	-7.0715639	2.9121771	3.2057447
54	H	-5.5113805	2.0806455	3.1911258
55	H	-6.3989248	2.2656316	-1.0058149
56	H	-7.5557044	1.8601932	0.2672812
57	H	-5.9524976	1.1038704	0.2470665
58	H	-6.2336036	5.8689799	1.3086905
59	H	-6.5165204	5.312273	-0.3415997
60	H	-7.7234736	5.0059565	0.911353
61	H	6.254808	5.8759906	-1.1810939
62	H	7.4950032	4.9953152	-2.0805383
63	H	5.9029061	5.2622655	-2.797589
64	H	5.641109	1.0785874	-1.9060189
65	H	7.1298201	1.8289733	-2.5092628
66	H	5.5780146	2.1974184	-3.2701966
67	H	6.5288774	3.8927358	1.2241128
68	H	6.3298315	2.1574994	0.9590823
69	H	7.7728797	2.9837064	0.3584759

WILEY-VCH

70	H	-9.9762162	-6.6276999	-2.3407556
71	H	-9.3262662	-5.1707071	-3.0926269
72	H	-8.4572519	-6.6983207	-3.2341101
73	H	-8.0569296	-6.6938114	-0.7456763
74	H	-8.9198468	-5.1778959	-0.6047839
75	H	-7.2102791	-4.0686901	-2.062516
76	H	-6.3467041	-5.5857288	-2.2032466
77	H	-5.9773897	-5.607279	0.2749735
78	H	-6.8429212	-4.089854	0.417173
79	H	-5.1295219	-2.9726902	-1.0332527
80	H	-4.2609085	-4.4966842	-1.1763484
81	H	-3.8877257	-4.5244531	1.2920091
82	H	-4.7627575	-2.990419	1.4383125
83	H	9.9678958	-6.6502325	2.3282172
84	H	9.3234295	-5.1917906	3.0819751
85	H	8.4505959	-6.7171123	3.2246717
86	H	8.045193	-6.7106311	0.7370786
87	H	8.9118531	-5.1969976	0.5949212
88	H	7.2081651	-4.0837338	2.0564899
89	H	6.3409242	-5.5985289	2.1985538
90	H	5.9666303	-5.6185533	-0.2789293
91	H	6.8356154	-4.1033032	-0.4225646
92	H	5.1276599	-2.982086	1.0312721
93	H	4.2557869	-4.5040645	1.1760623
94	H	3.8782499	-4.5309371	-1.2917868
95	H	4.7561378	-2.9987573	-1.4394562

Energy and cartesian coordinates for **5a**:

Total Energy = -2914.99176313 Hartree

No imaginary frequencies.

Table S6: Cartesian coordinates 5a.

Tag	Symbol	X	Y	Z
1	C	0.7275597	4.3171816	0.1835591
2	C	-0.7225276	4.318064	0.1835311
3	C	-1.4502291	3.1173057	-0.0101388
4	C	-0.7248449	1.9110942	-0.2031517
5	C	0.7269864	1.9102133	-0.2031172
6	C	1.4538183	3.115543	-0.0100671
7	C	-2.8584622	3.1050555	-0.0141162
8	C	2.8620356	3.1016224	-0.0139867
9	N	1.4127314	5.4609681	0.3679759
10	C	0.7297863	6.5773279	0.5482906
11	C	-0.7220139	6.5782132	0.5482611

12	N	-1.4063142	5.4626845	0.3679243
13	C	4.074521	3.0459115	-0.0255923
14	C	-4.0710114	3.0507371	-0.0257734
15	N	-1.4073071	0.7712863	-0.385497
16	N	1.4080811	0.7695871	-0.3854484
17	C	0.7279345	-0.3489693	-0.5639115
18	C	-0.728506	-0.348091	-0.5639278
19	C	-1.3954928	-1.5954771	-0.7619292
20	C	-0.7093528	-2.7672303	-0.9482079
21	C	0.7058674	-2.7680845	-0.9481932
22	C	1.3934148	-1.5971544	-0.7619099
23	C	1.7054496	-3.880798	-1.1254713
24	N	2.9564317	-3.272832	-1.0276531
25	C	2.8668634	-1.8974468	-0.8098648
26	C	-2.8692949	-1.894001	-0.8098499
27	N	-2.9605165	-3.2692828	-1.0276448
28	C	-1.7102647	-3.8787373	-1.1255139
29	O	-1.5249637	-5.0515542	-1.3174886
30	O	-3.8100836	-1.1508132	-0.6974153
31	O	1.5187503	-5.0533961	-1.3174216
32	O	3.8085447	-1.155392	-0.6974128
33	C	1.4222491	7.8124177	0.7479703
34	C	0.7213013	8.9615589	0.9335795
35	C	-0.7106066	8.9624341	0.9335526
36	C	-1.4129617	7.8141541	0.7479206
37	Si	5.9047681	2.8442405	-0.0704578
38	C	6.6971316	4.3861897	0.6541734
39	C	6.4149759	2.6210327	-1.8646186
40	C	6.3408445	1.3235056	0.9375142
41	Si	-5.9015054	2.8512281	-0.0707553
42	C	-6.4120296	2.6302149	-1.865096
43	C	-6.692041	4.3934669	0.6552654
44	C	-6.3393008	1.3301761	0.9359901
45	C	8.4295344	-7.1042885	2.5879482
46	C	7.9026046	-6.570989	1.2571398
47	C	6.5804455	-5.8148604	1.3906705
48	C	6.0467309	-5.2773959	0.0626114
49	C	4.7251759	-4.5227671	0.2040168
50	C	4.2162158	-3.9963504	-1.1381677
51	C	-8.4368692	-7.0954426	2.5885787
52	C	-7.9097677	-6.5623967	1.2577368
53	C	-6.5868127	-5.8076257	1.3910667
54	C	-6.05287	-5.2704827	0.06297
55	C	-4.7305011	-4.5172468	0.2042003
56	C	-4.2211432	-3.9913495	-1.1380382

WILEY-VCH

57	H	2.5037792	7.7886023	0.7445174
58	H	1.2445764	9.8971508	1.0849438
59	H	-1.2327432	9.8986648	1.0849003
60	H	-2.4945198	7.7916696	0.7444344
61	H	6.3817373	4.5425556	1.6878406
62	H	7.7870783	4.3027491	0.6444102
63	H	6.4228527	5.2755699	0.0830519
64	H	5.9368147	1.7392451	-2.2951846
65	H	7.4977585	2.4923409	-1.9440321
66	H	6.1334389	3.4864395	-2.4678999
67	H	6.0510858	1.4471003	1.9831387
68	H	5.8229606	0.4460072	0.5455014
69	H	7.4169892	1.1328193	0.9063405
70	H	-5.9349835	1.7482253	-2.2964816
71	H	-7.4949738	2.5029473	-1.944585
72	H	-6.1294357	3.4958174	-2.4676037
73	H	-6.376324	4.5485753	1.689023
74	H	-7.7820842	4.3113017	0.645575
75	H	-6.4168091	5.2830229	0.0848741
76	H	-5.8220244	0.4524715	0.5436393
77	H	-6.0498748	1.4528802	1.9818134
78	H	-7.4156003	1.140449	0.9042564
79	H	9.3729626	-7.6381367	2.4579952
80	H	8.6032279	-6.2918937	3.2981011
81	H	7.7177104	-7.7955863	3.0460103
82	H	7.7723261	-7.4028608	0.5568423
83	H	8.6511837	-5.9103674	0.806908
84	H	6.710242	-4.9823123	2.0915596
85	H	5.8307662	-6.4757961	1.8404095
86	H	5.915708	-6.1103206	-0.6373836
87	H	6.7963071	-4.6158471	-0.3863986
88	H	4.8508783	-3.6810112	0.8910851
89	H	3.9670985	-5.1816667	0.6369153
90	H	4.0505593	-4.8208682	-1.8322867
91	H	4.940052	-3.3094516	-1.5777394
92	H	-9.3808651	-7.6283216	2.4587728
93	H	-7.7256104	-7.7875461	3.0463029
94	H	-8.6095555	-6.2830212	3.2989466
95	H	-8.6578052	-5.9009313	0.8078443
96	H	-7.7805117	-7.3942538	0.5572322
97	H	-5.8376925	-6.4693996	1.8405054
98	H	-6.715589	-4.9750685	2.0921329
99	H	-6.8018686	-4.6080868	-0.3857537
100	H	-5.9228662	-6.1034262	-0.6371934
101	H	-3.9730638	-5.1769714	0.6369656

WILEY

102	H	-4.8552053	-3.6753871	0.8913217
103	H	-4.9442544	-3.3036291	-1.577517
104	H	-4.0564956	-4.816048	-1.8321826

6) FMO Distribution

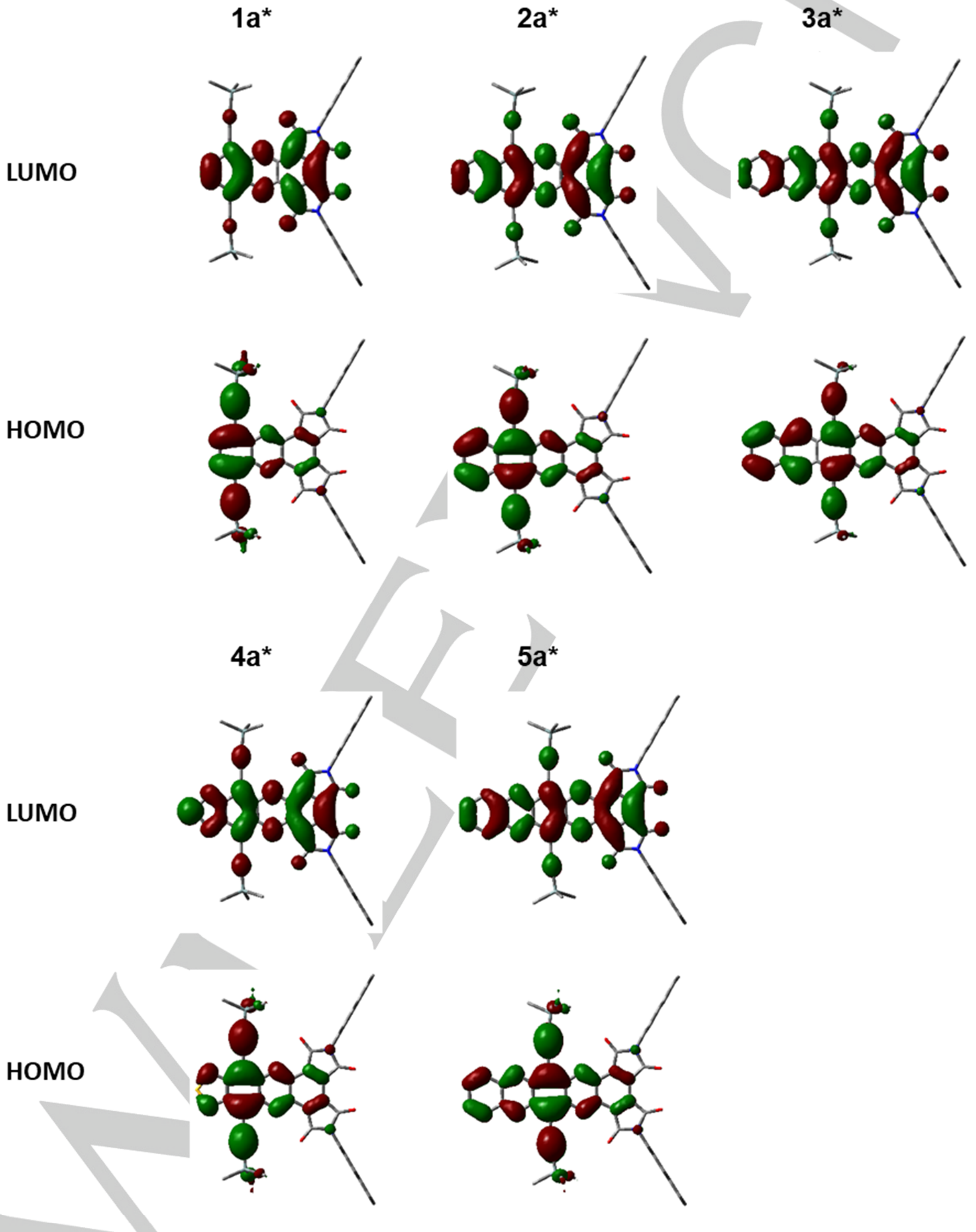

Figure S6. Distribution of FMOs of TMS-substituted model compounds calculated using Gaussian16 B3LYP/def2SVP//Gaussian16 B3LYP/def2TZVP, TIPS-substituents were replaced with TMS groups.

7) NICS(1)zz calculations

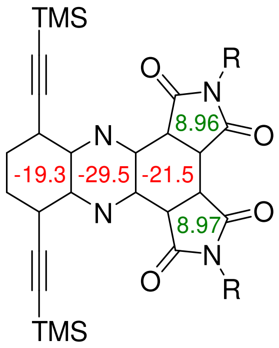

1a*

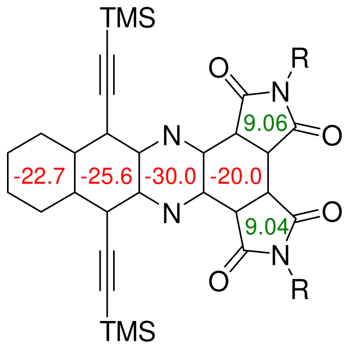

2a*

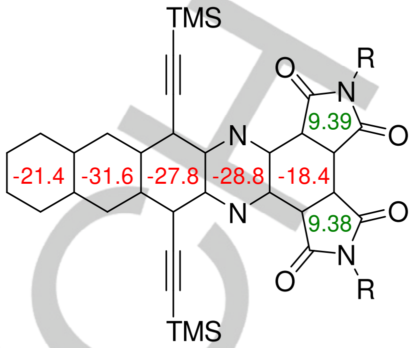

3a*

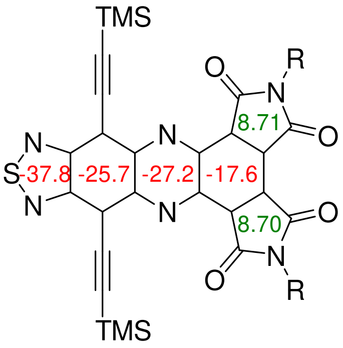

4a*

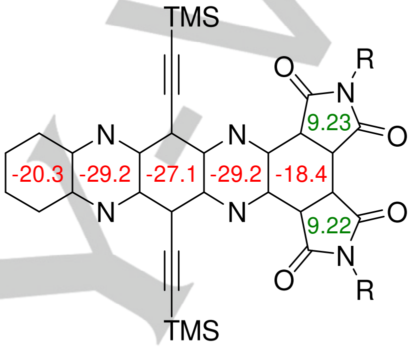

5a*

Figure S7. NICS(1)zz values calculated using Gaussian16 B3LYP/def2SVP//Gaussian16 B3LYP/def2TZVP, TIPS-substituents were replaced with TMS groups. Cartesian axes were adjusted from the geometry optimization reported in chapter 5) with Avogadro 1.2.0 to orient the Z-axis perpendicular to the acene XY-plane.

WILEY-VCH

8) AICD Calculations

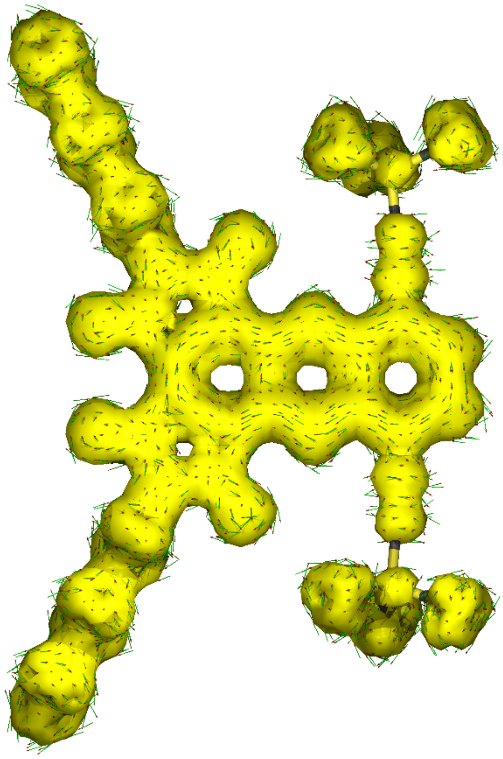

Figure S8: AICD plot for **1a**.

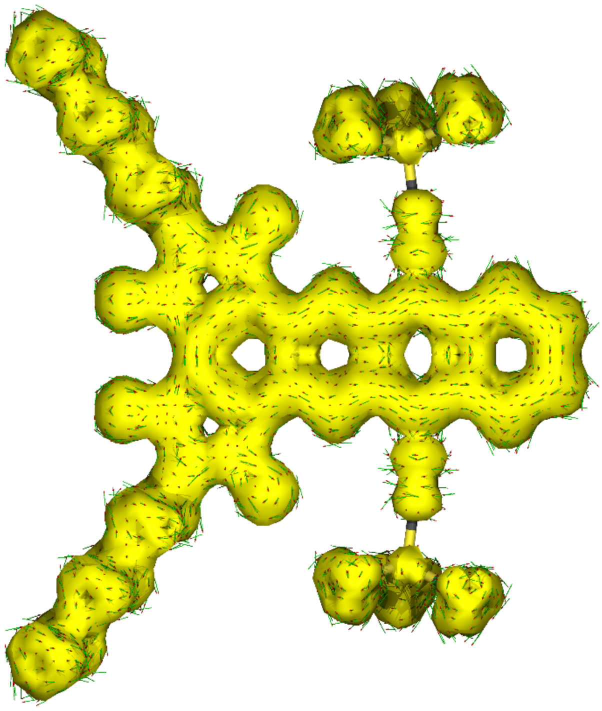

Figure S9: AICD plot for **2a**.

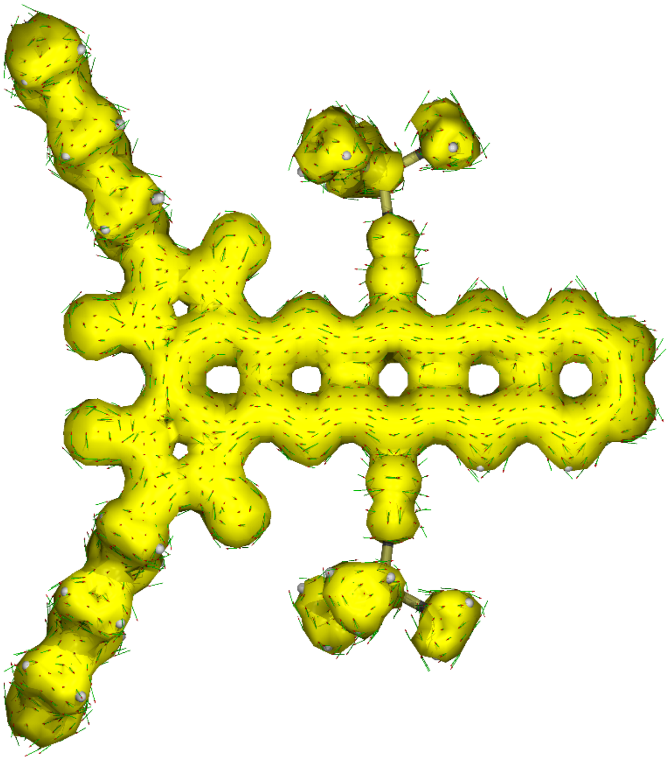

Figure S10: AICD plot for **3a**.

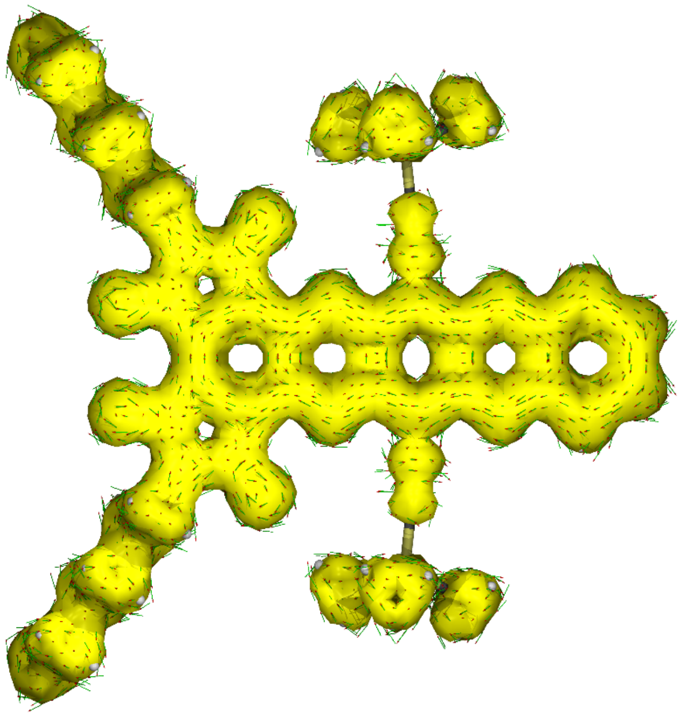

Figure S11: AICD plot for **4a**.

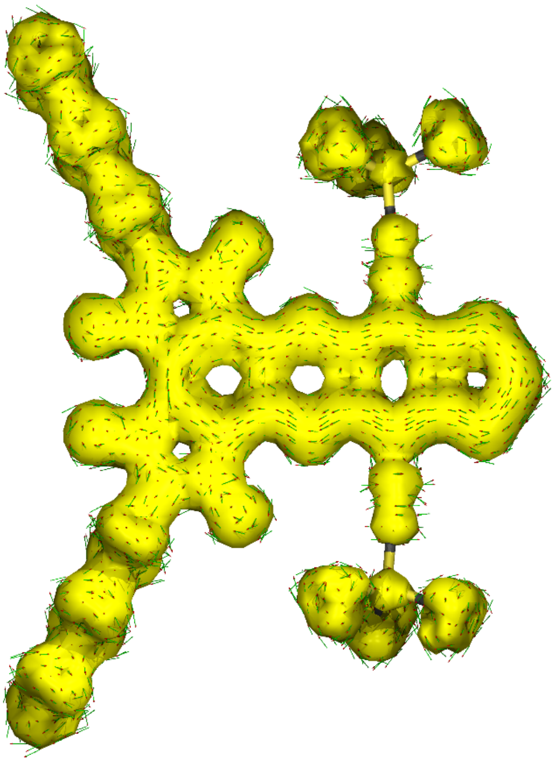

Figure S12: AICD plot for **5a**.

WILEY

9) NMR Spectroscopy

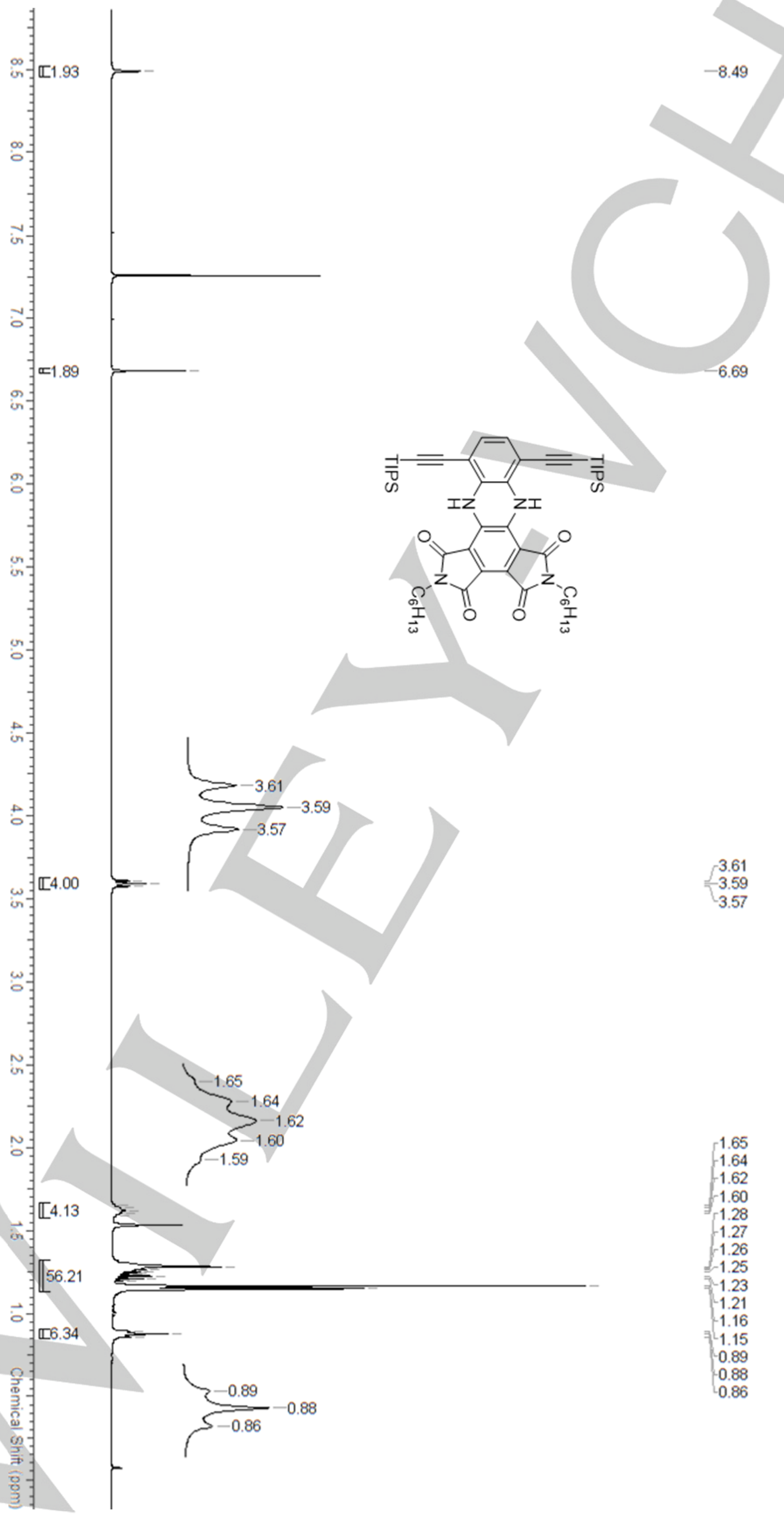

Figure S13. ^{1}H NMR spectrum (400.3 MHz, $CDCl_3$, 295 K) of **1-H$_2$**.

WILEY-VCH

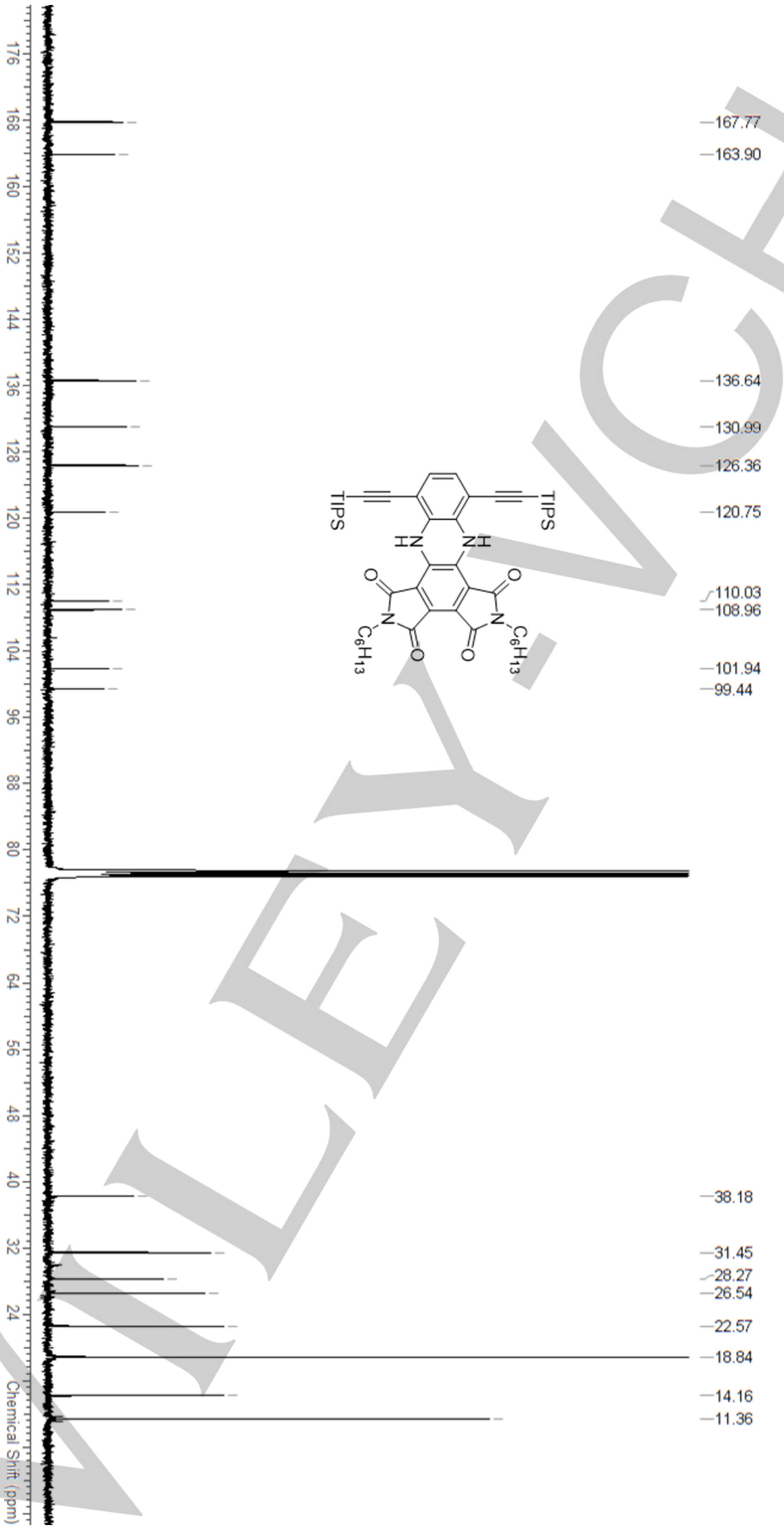

Figure S14. ^{13}C NMR spectrum (100.7 MHz, $CDCl_3$, 295 K) of **1-H$_2$**.

WILEY-VCH

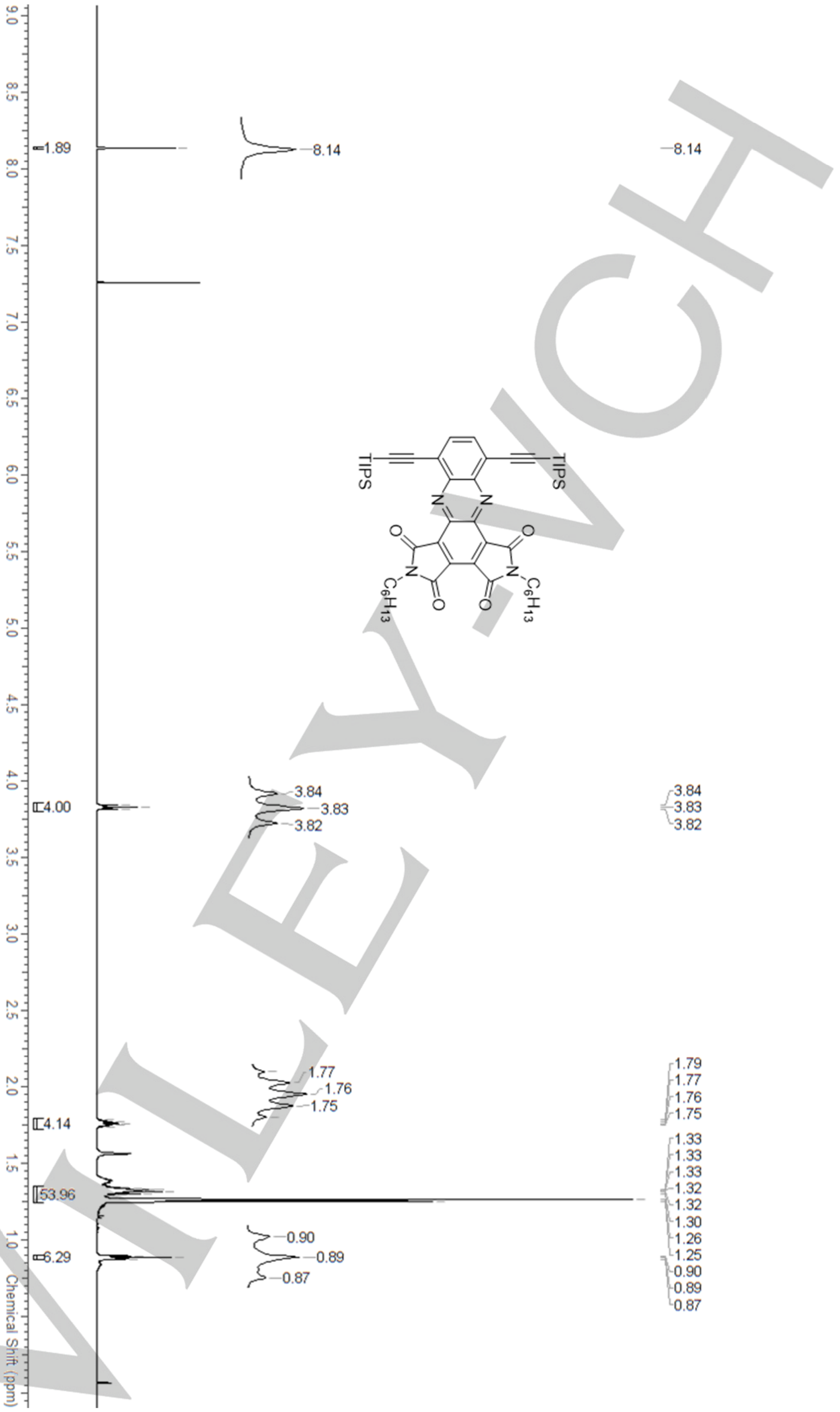

Figure S15. ^{1}H NMR spectrum (600.2 MHz, $CDCl_3$, 295 K) of **1a**.

WILEY-VCH

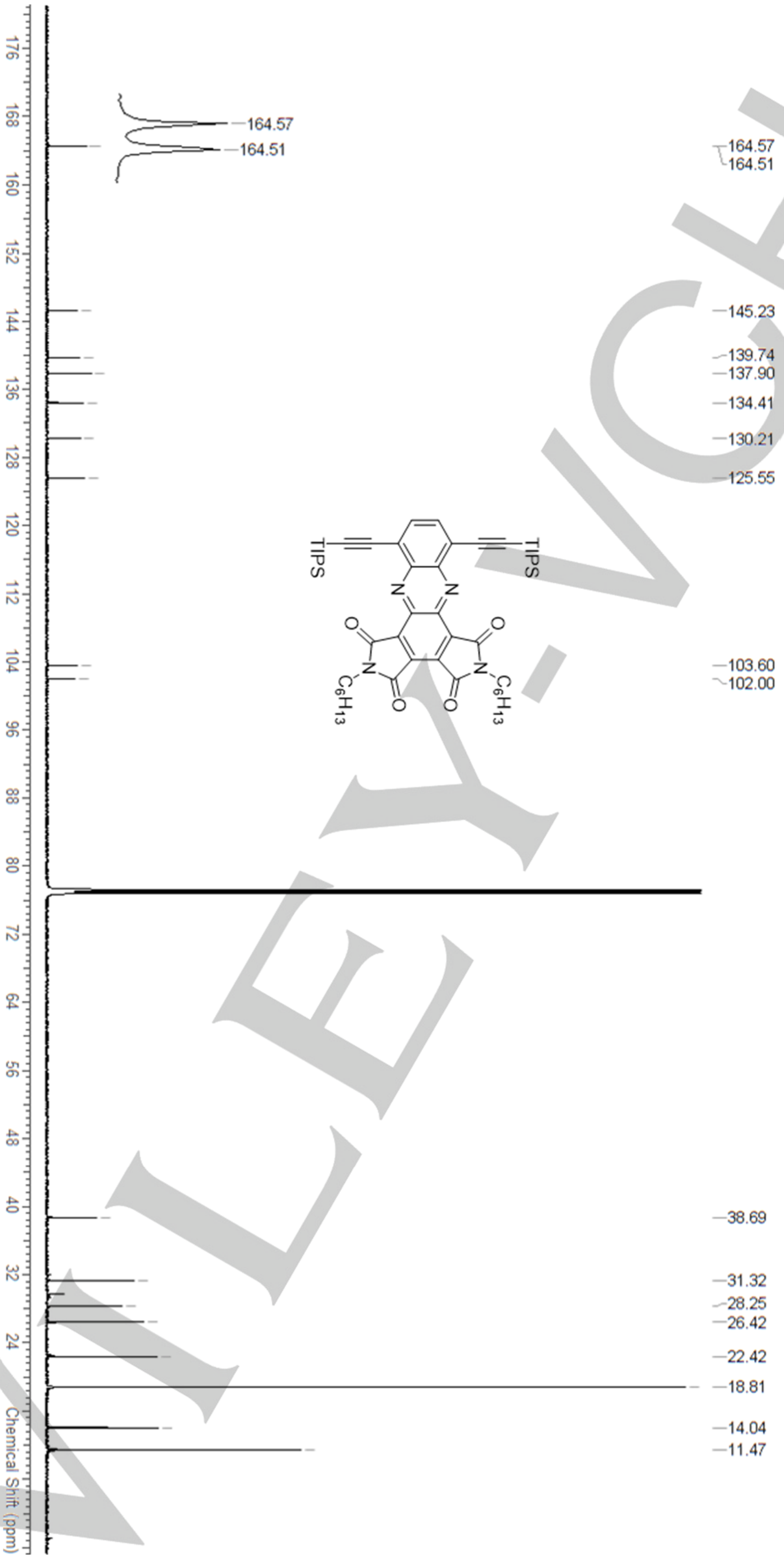

Figure S16. ^{13}C NMR spectrum (150.9 MHz, $CDCl_3$, 295 K) of **1a**.

WILEY-VCH

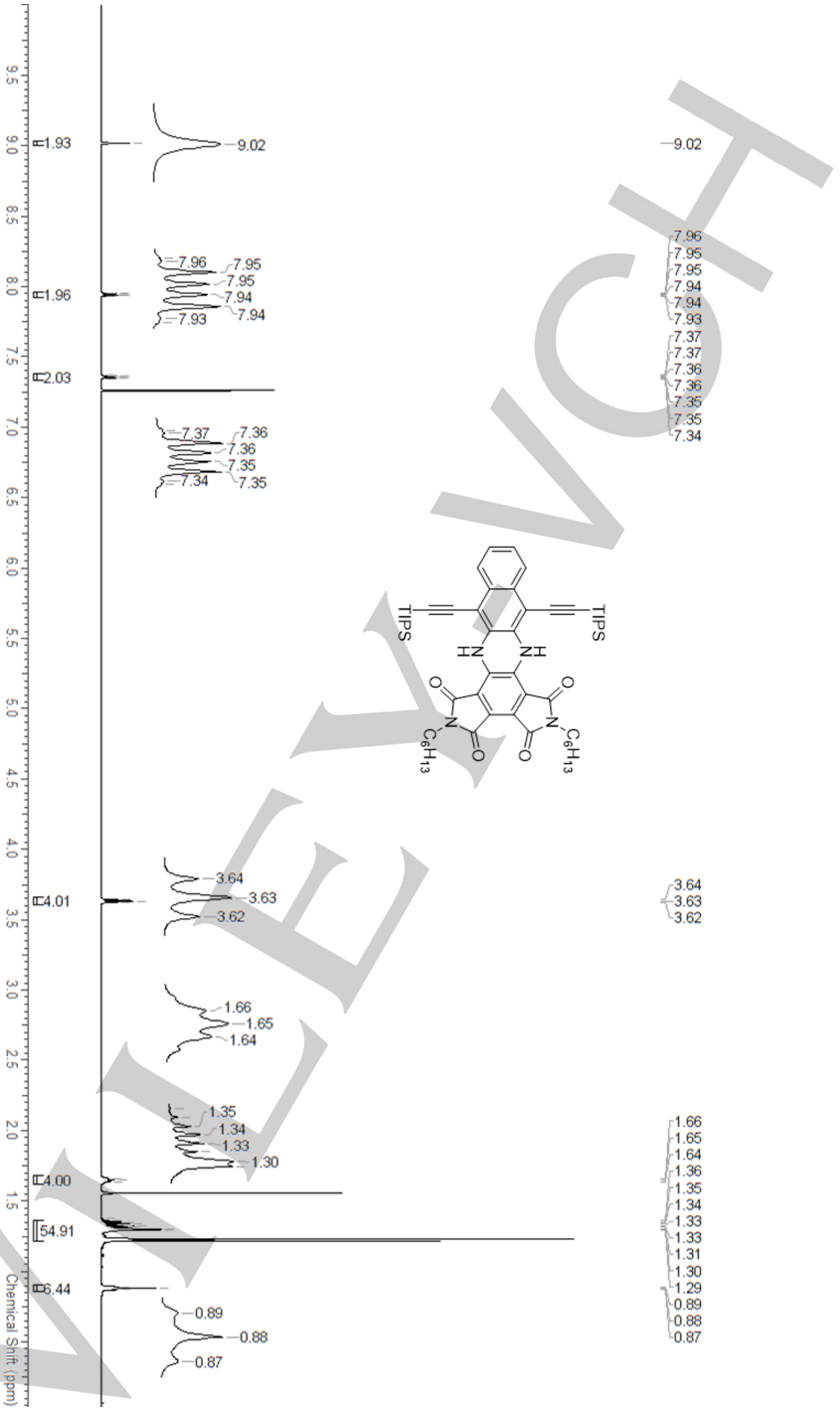

Figure S17 ^{1}H NMR spectrum (600.2 MHz, $CDCl_3$, 295 K) of **2-H$_2$**.

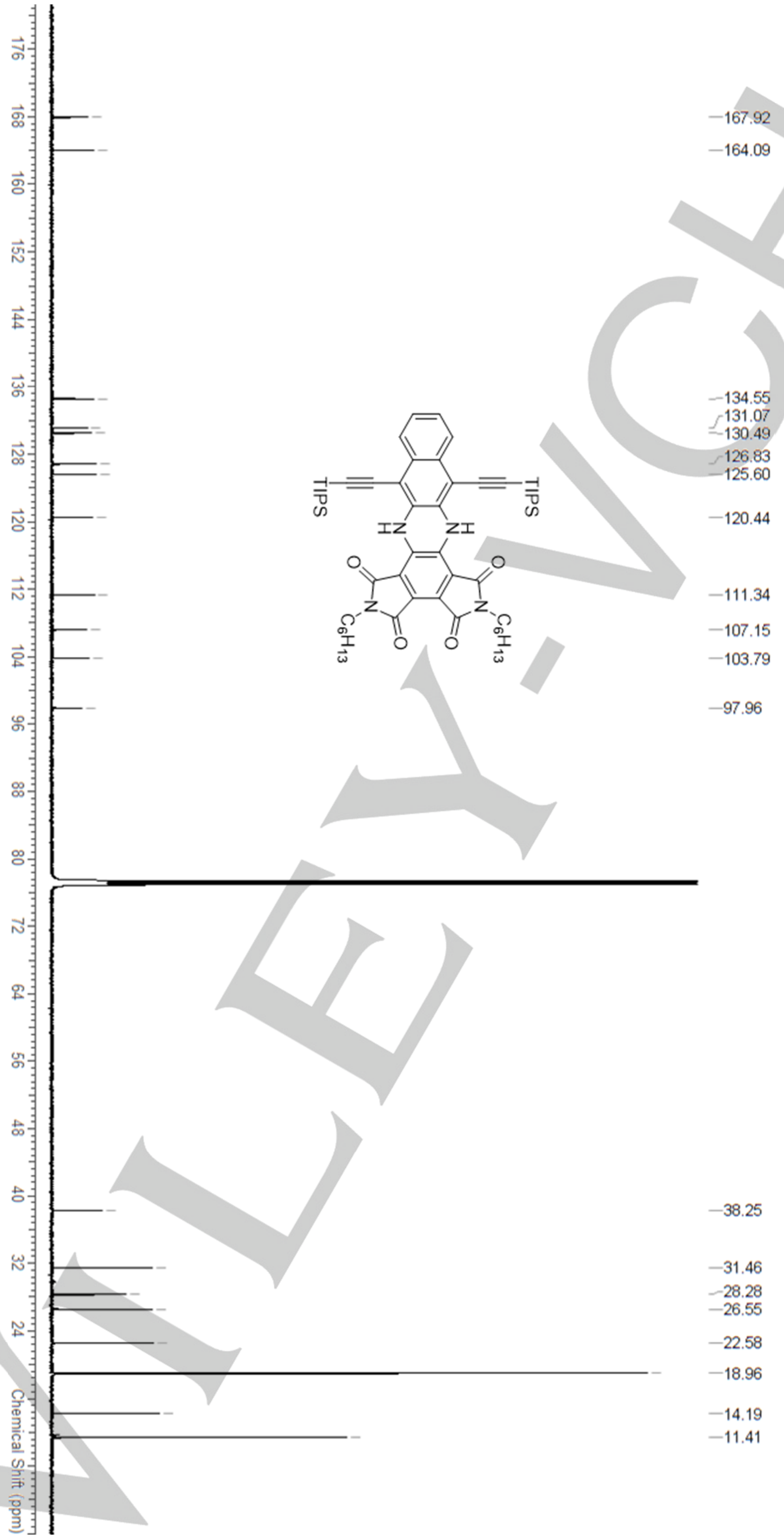

Figure S18. ^{13}C NMR spectrum (150.9 MHz, $CDCl_3$, 295 K) of **2-H_2**.

WILEY-VCH

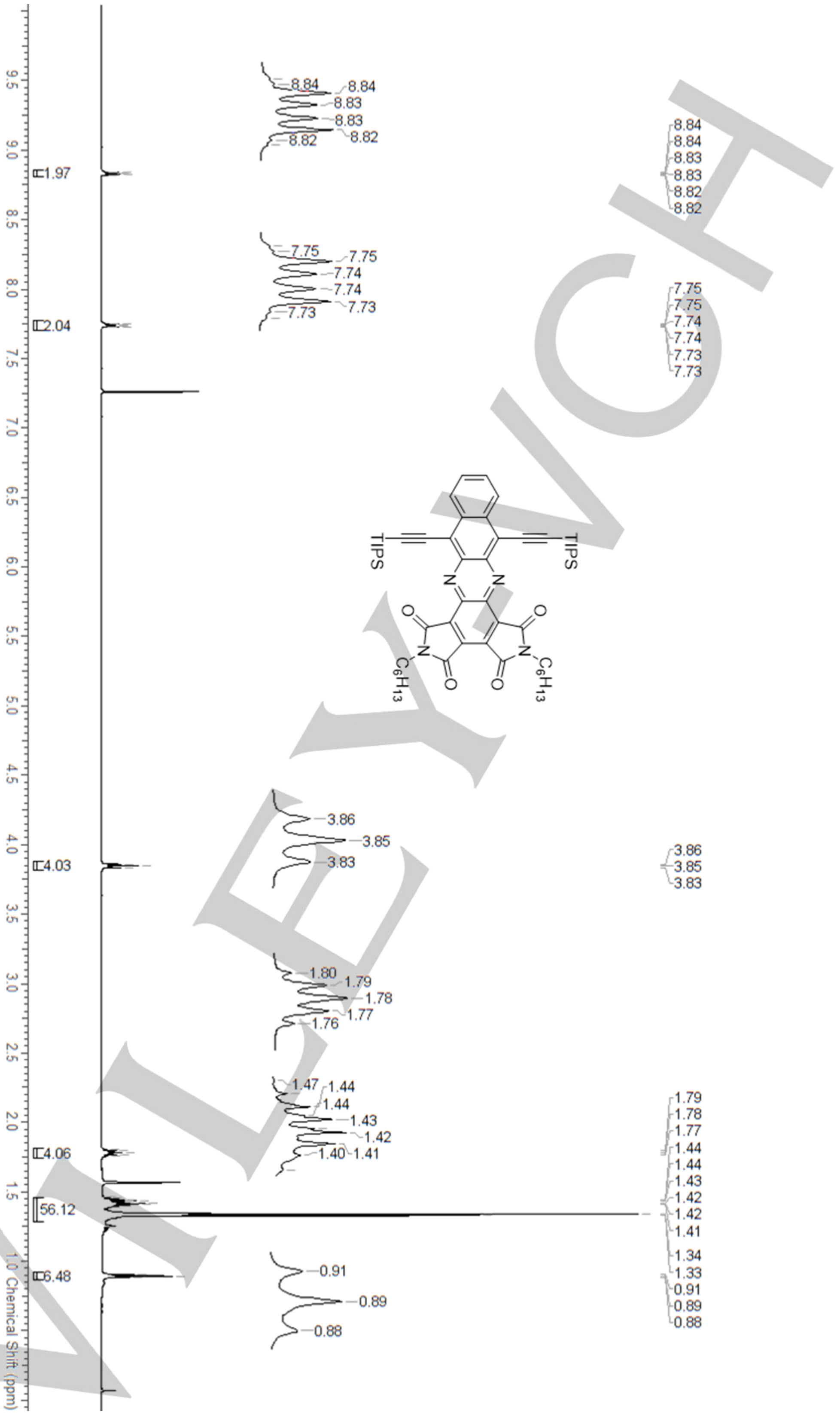

Figure S19. ^{1}H NMR spectrum (600.2 MHz, $CDCl_3$, 295 K) of **2a**.

WILEY-VCH

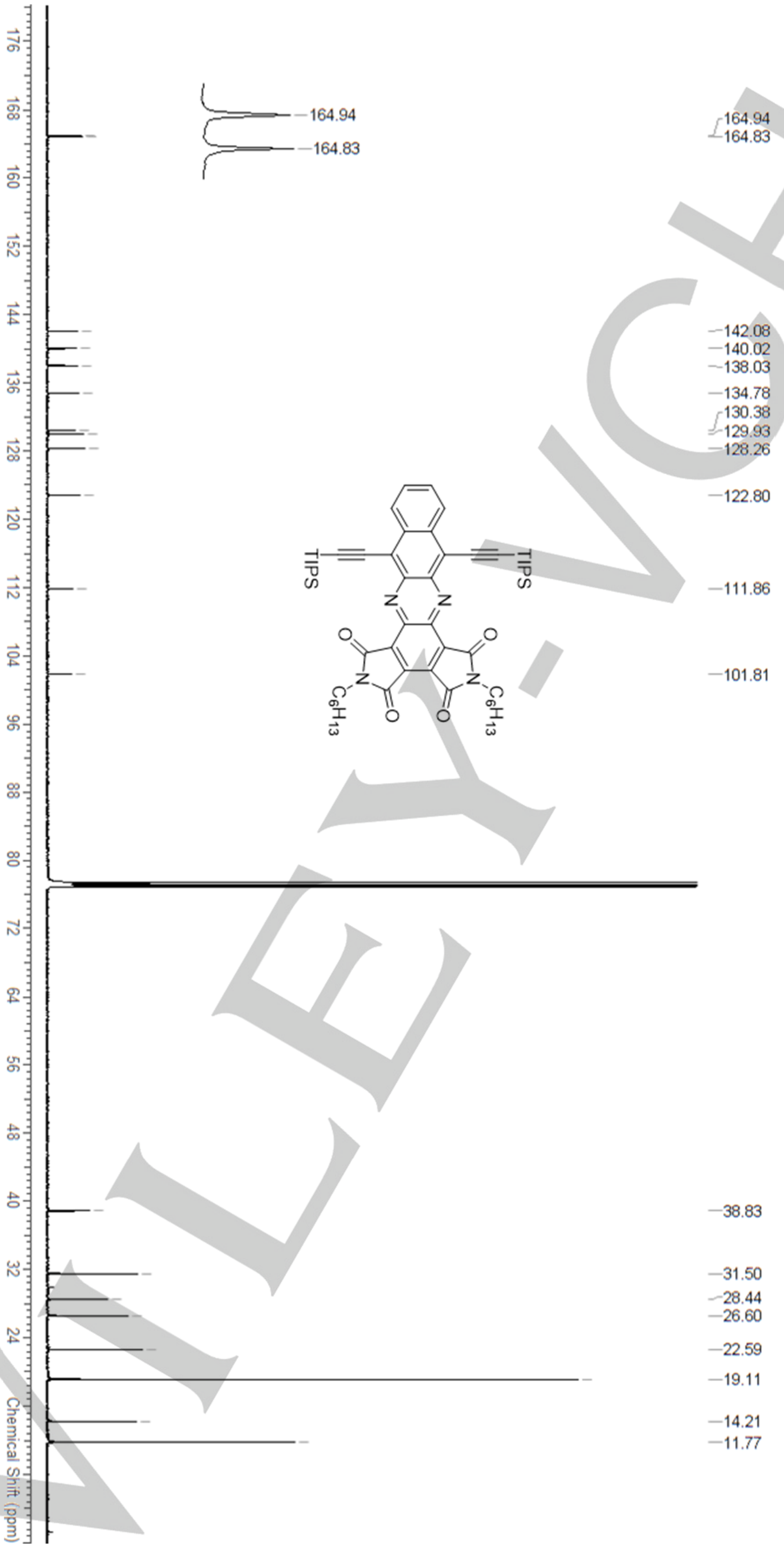

Figure S3. ^{13}C NMR spectrum (150.9 MHz, $CDCl_3$, 295 K) of **2a**.

WILEY-VCH

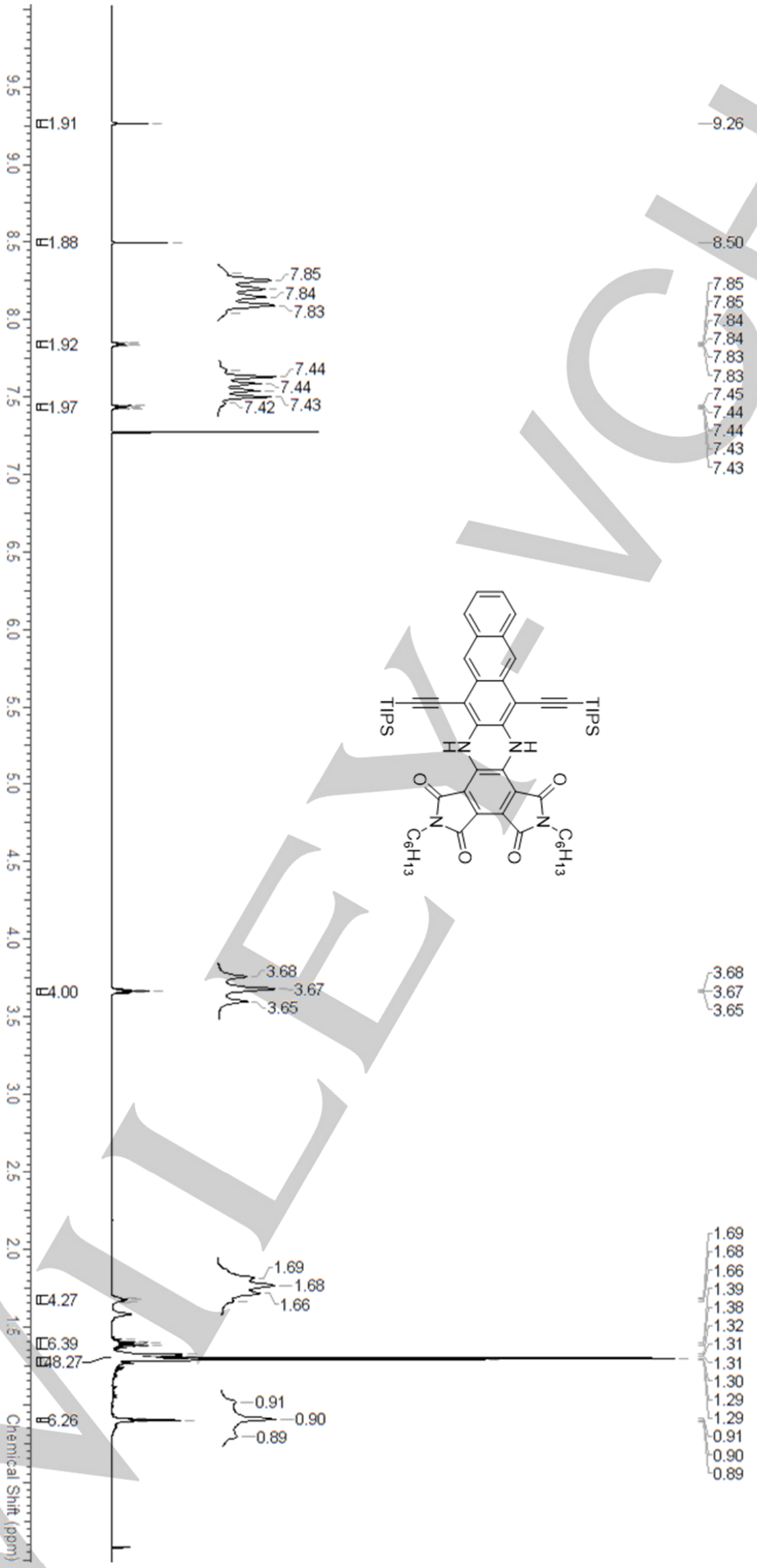

Figure S21. ^{1}H NMR spectrum (600.2 MHz, $CDCl_3$, 295 K) of **3-H$_2$**.

WILEY-VCH

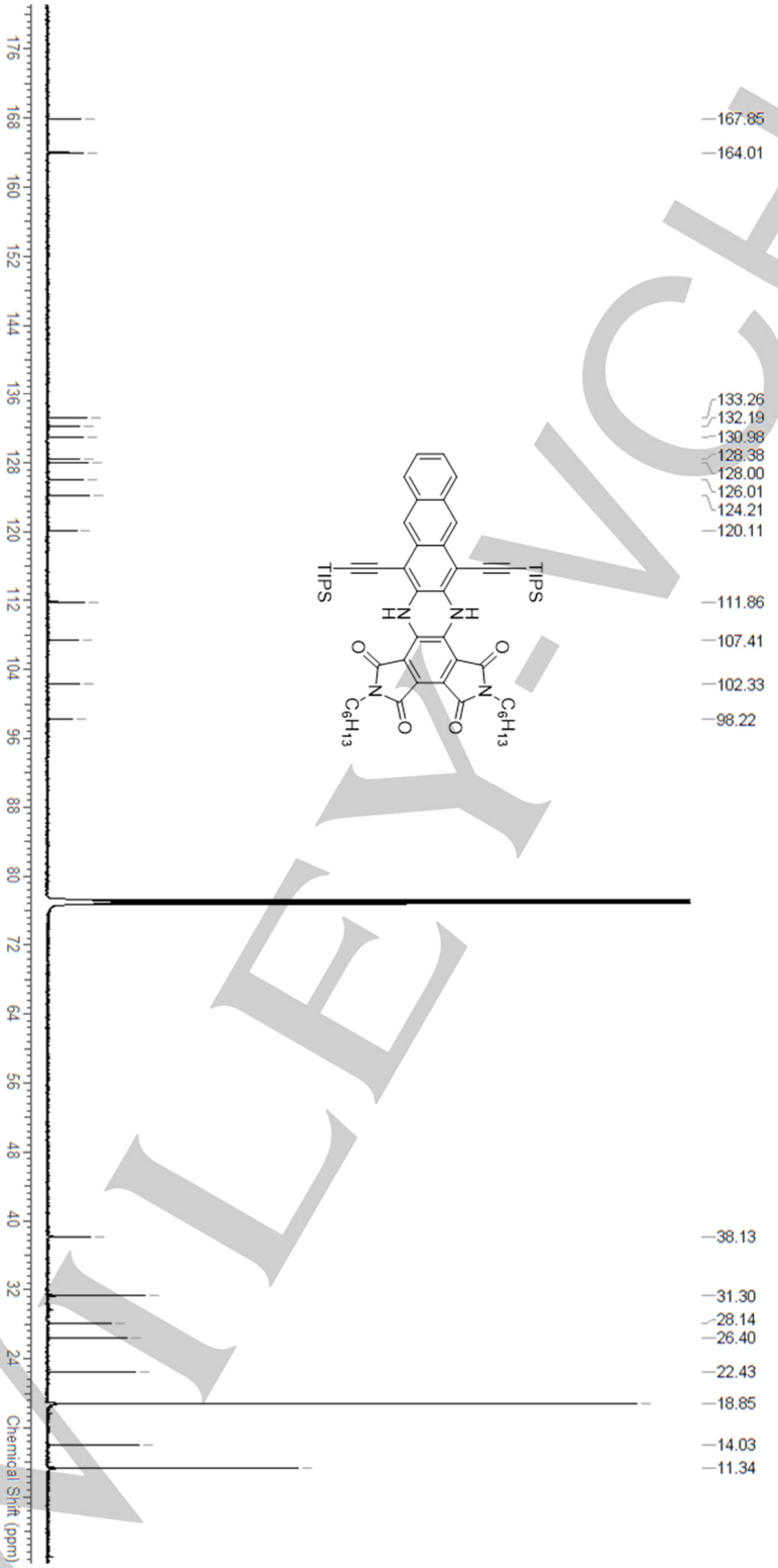

Figure S422. ^{13}C NMR spectrum (150.9 MHz, $CDCl_3$, 295 K) of **3-H₂**.

WILEY-VCH

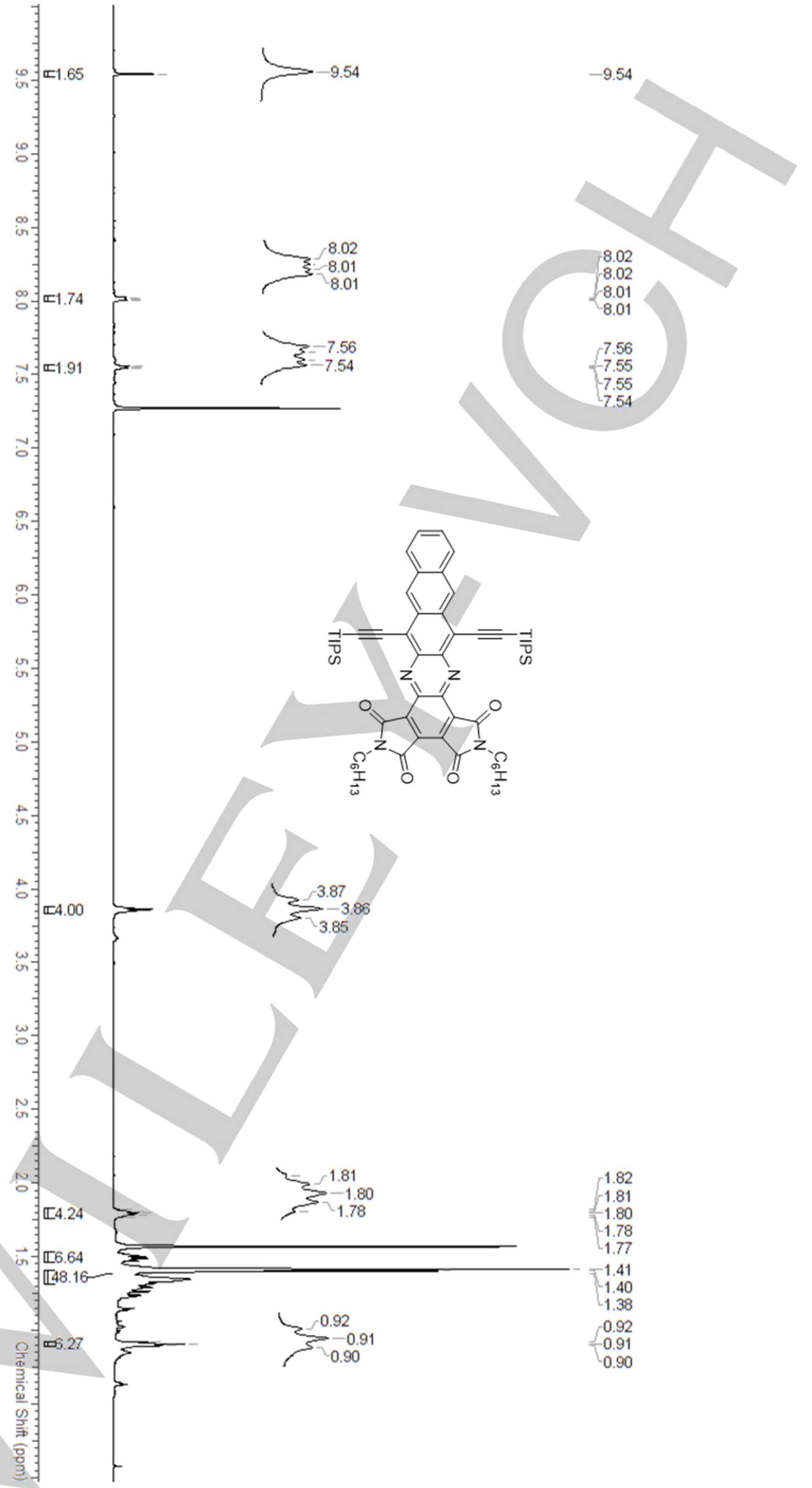

Figure S23. ^{1}H NMR spectrum (600.2 MHz, $CDCl_3$, 295 K) of **3a**.

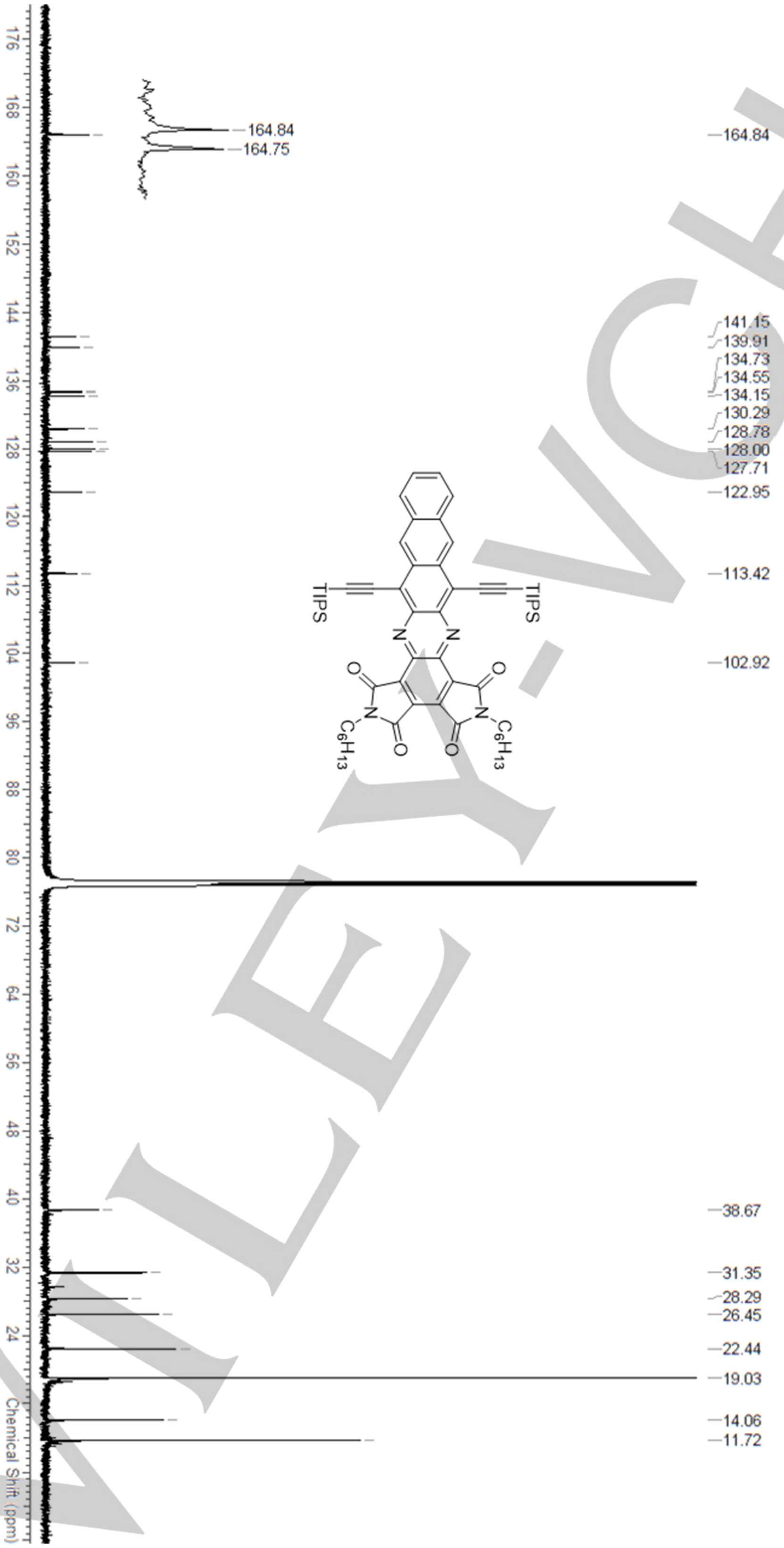

Figure S24. ^{13}C NMR spectrum (150.9 MHz, $CDCl_3$, 295 K) of **3a**.

WILEY-VCH

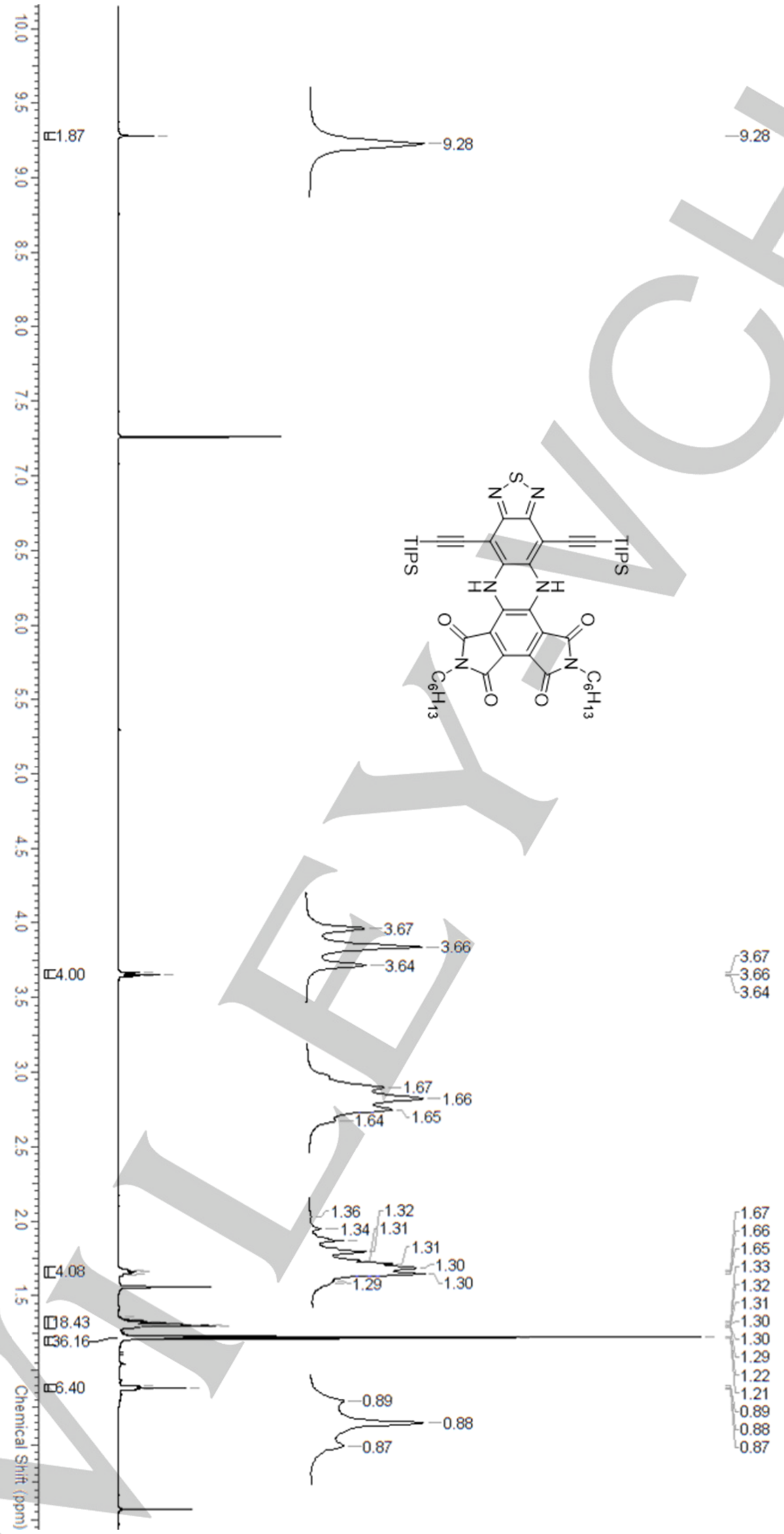

Figure S25. ^{1}H NMR spectrum (600.2 MHz, $CDCl_3$, 295 K) of **4-H$_2$**.

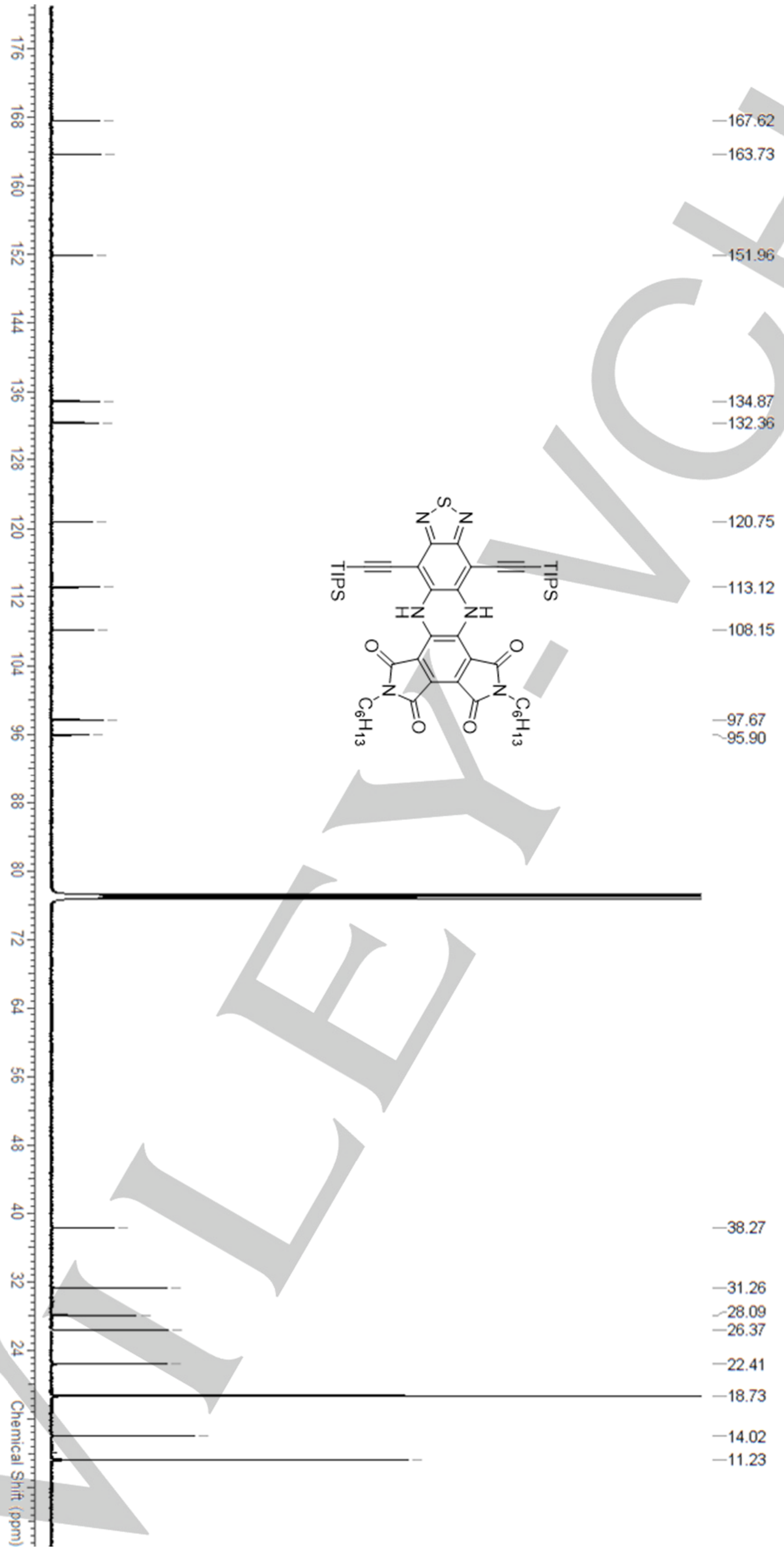

Figure S26. ^{13}C NMR spectrum (150.9 MHz, $CDCl_3$, 295 K) of **4-H₂**.

WILEY-VCH

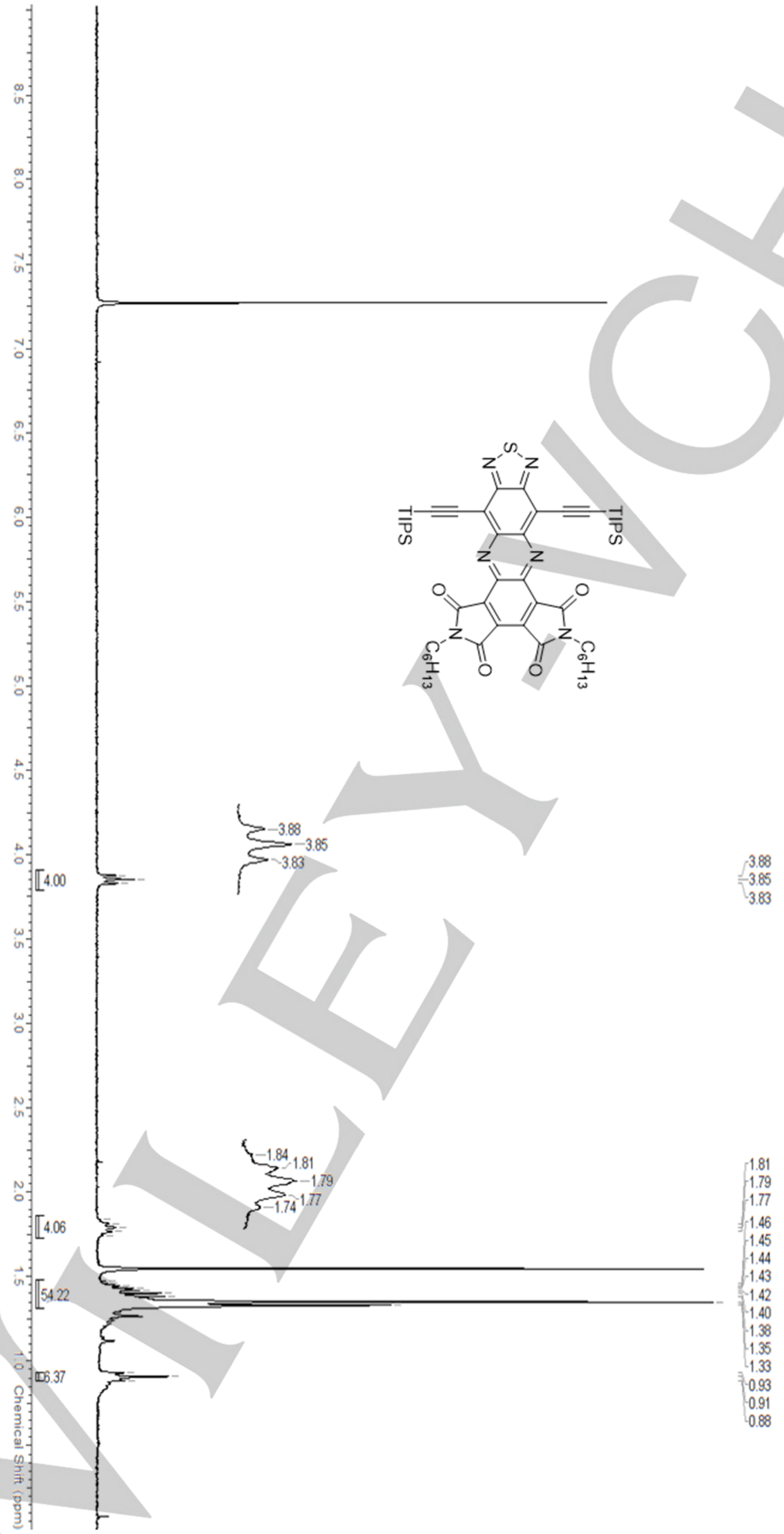

Figure S27. ^{1}H NMR spectrum (300.5 MHz, $CDCl_3$, 295 K) of **4a**.

WILEY-VCH

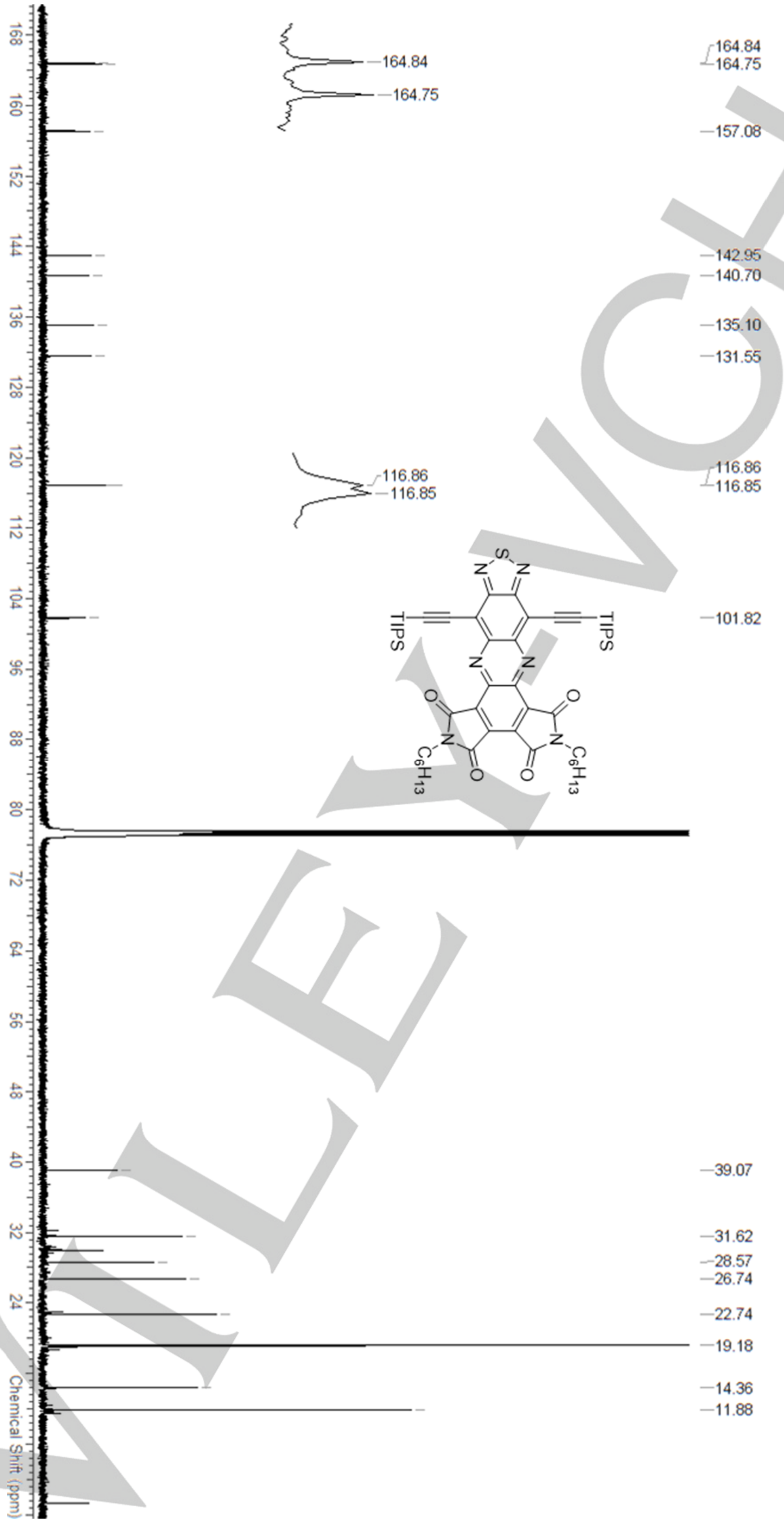

Figure S28. ^{13}C NMR spectrum (150.9 MHz, $CDCl_3$, 295 K) of **4a**.

WILEY-VCH

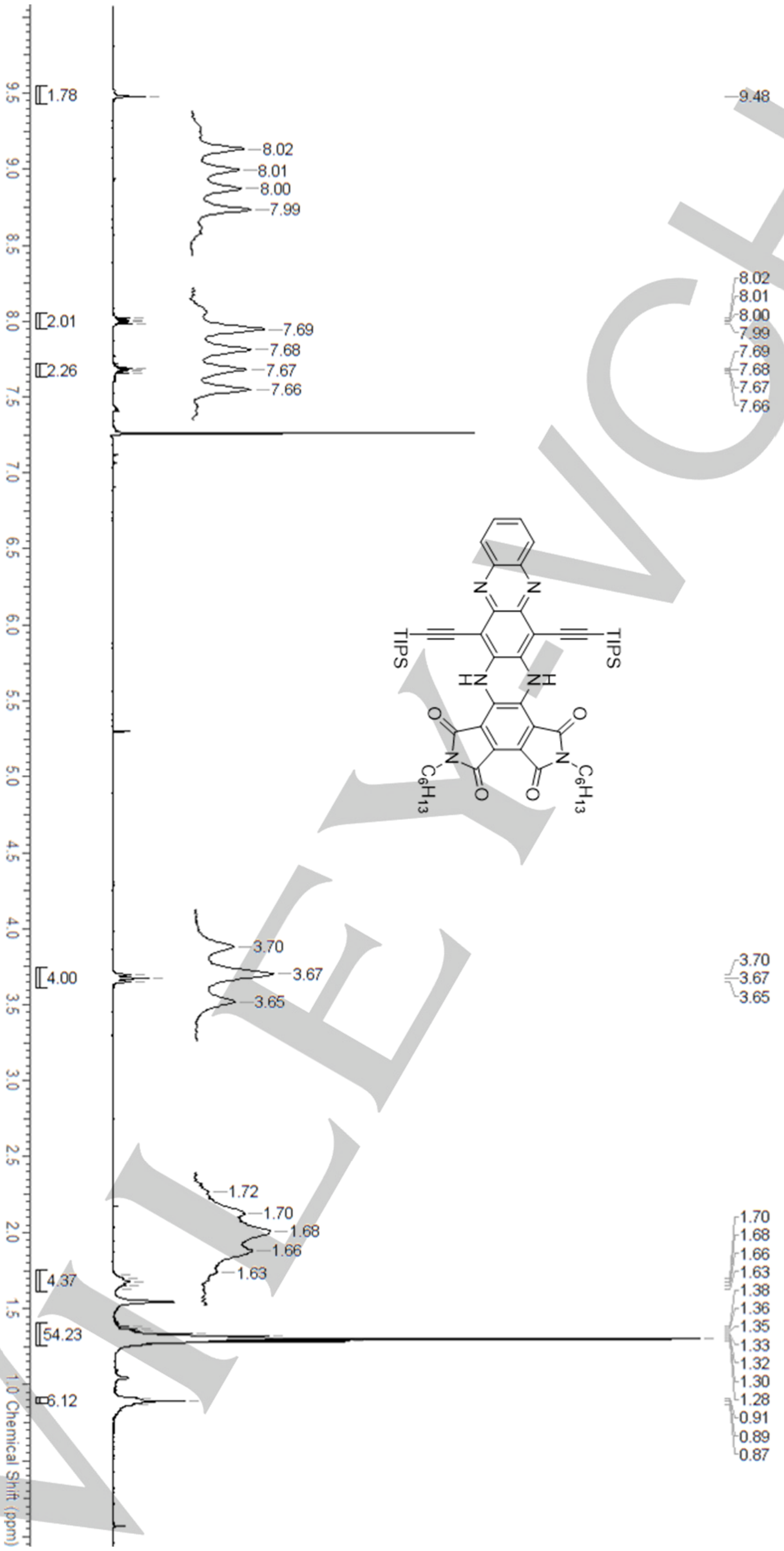

Figure S29. ^{1}H NMR spectrum (300.5 MHz, $CDCl_3$, 300 K) of **5-H_2**.

WILEY-VCH

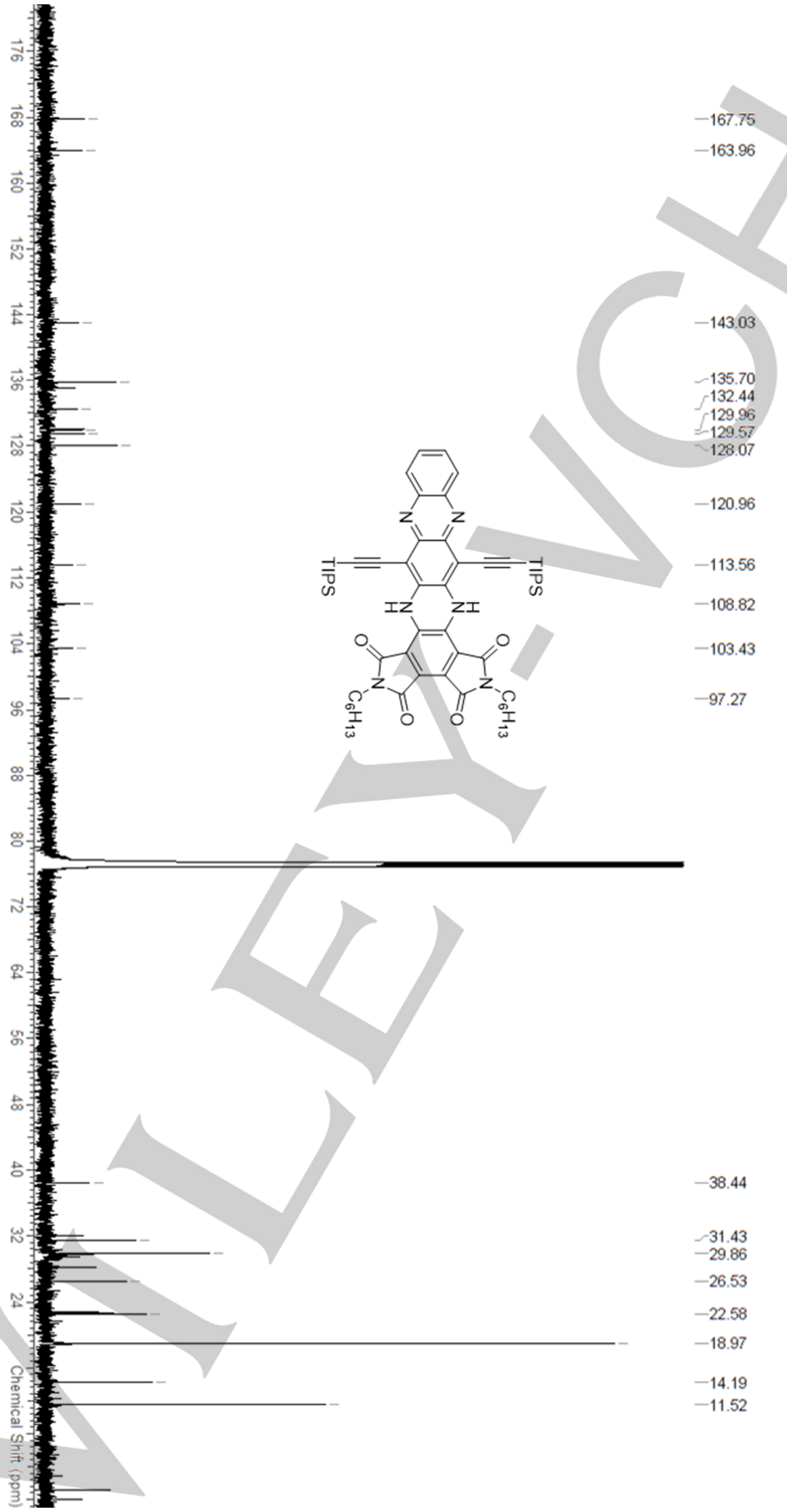

Figure S30. ^{13}C NMR spectrum (150.9 MHz, $CDCl_3$, 295 K) of **5-H$_2$**.

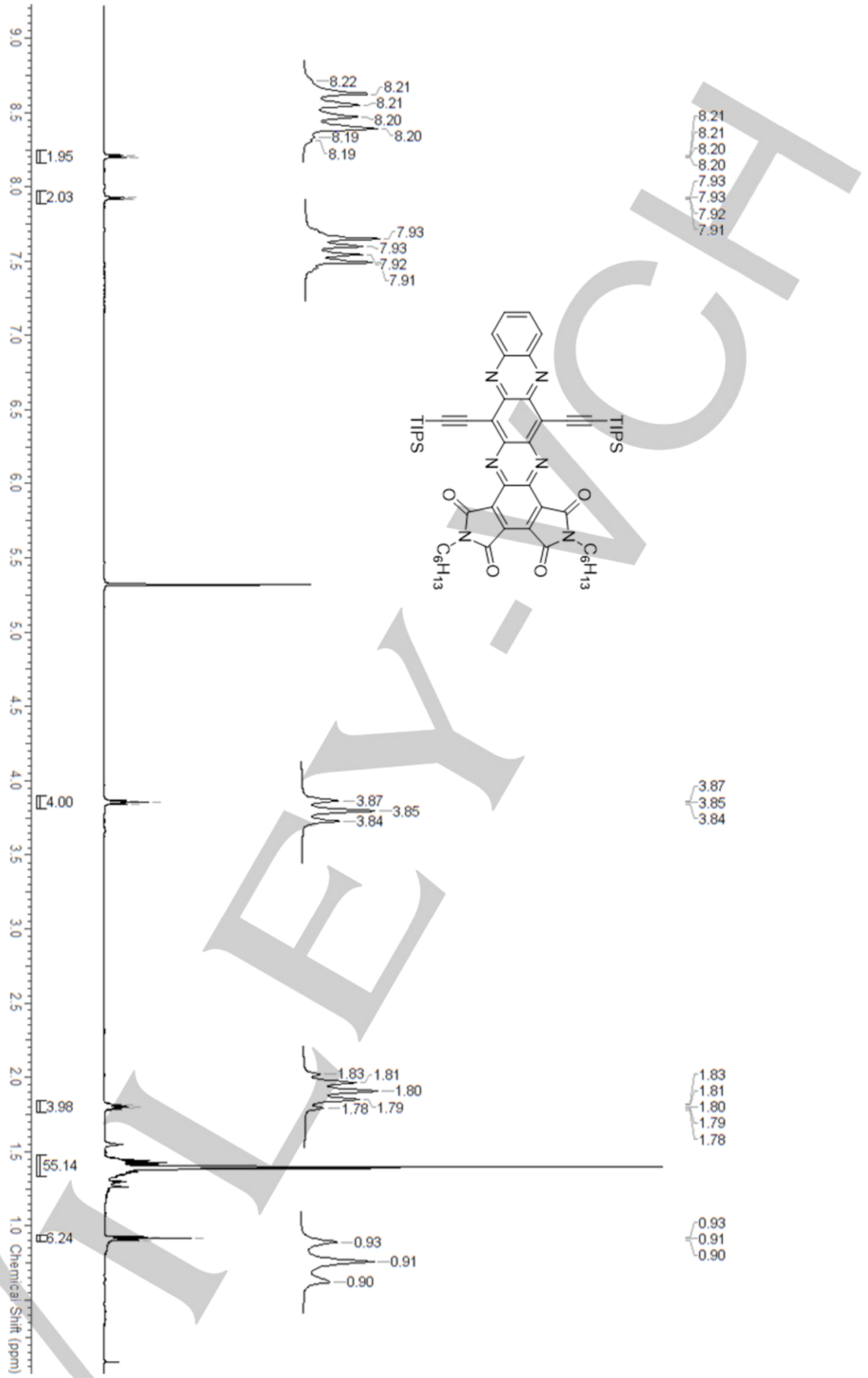

Figure S531. ^{1}H NMR spectrum (600.2 MHz, CD_2Cl_2, 295 K) of **5a**.

WILEY-VCH

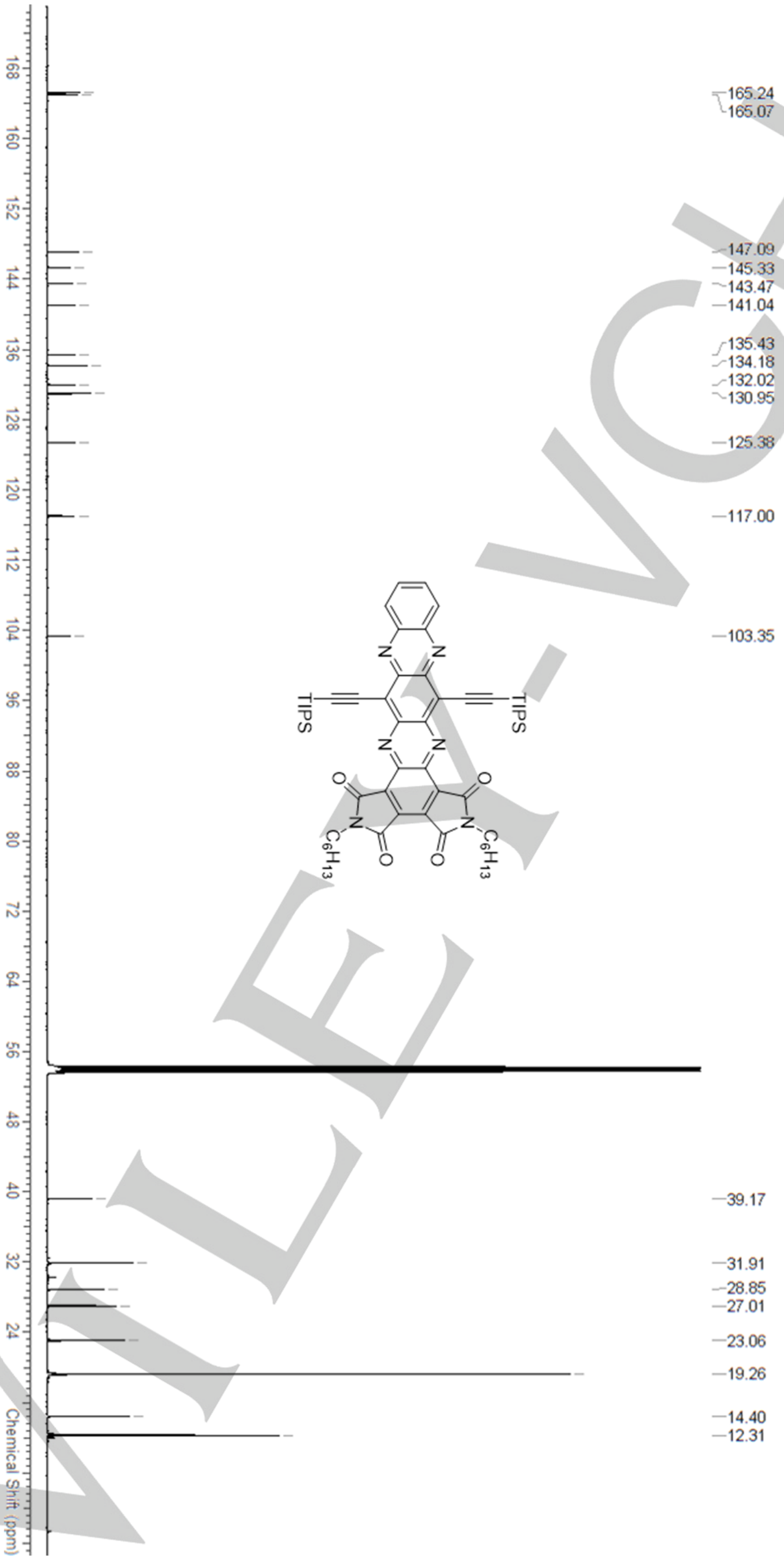

Figure S32. ^{13}C NMR spectrum (150.9 MHz, CD_2Cl_2, 295 K) of **5a**.

WILEY-VCH

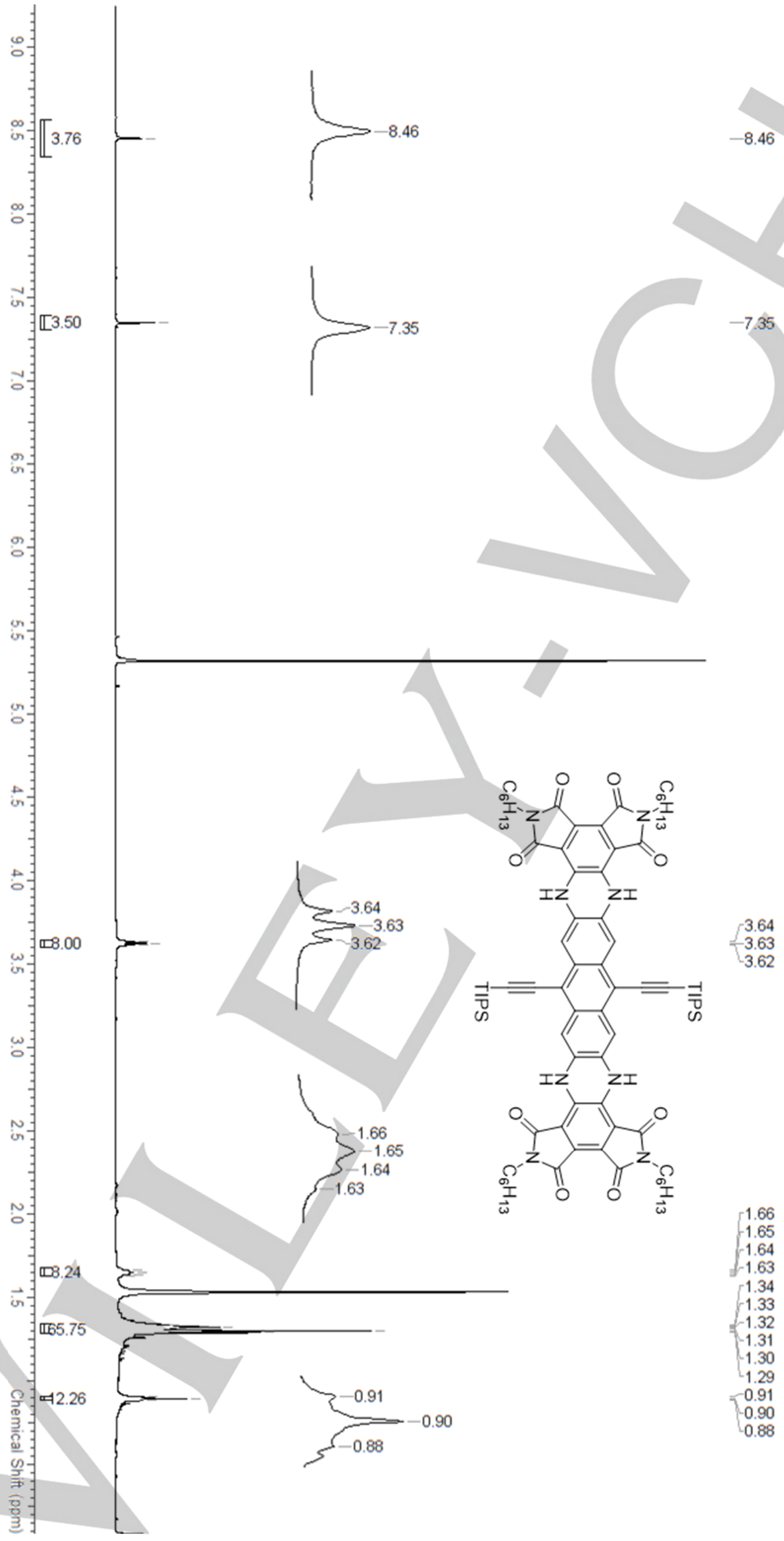

Figure S633. ^{1}H NMR spectrum (600.2 MHz, CD_2Cl_2, 295 K) of **12-H$_4$**.

WILEY-VCH

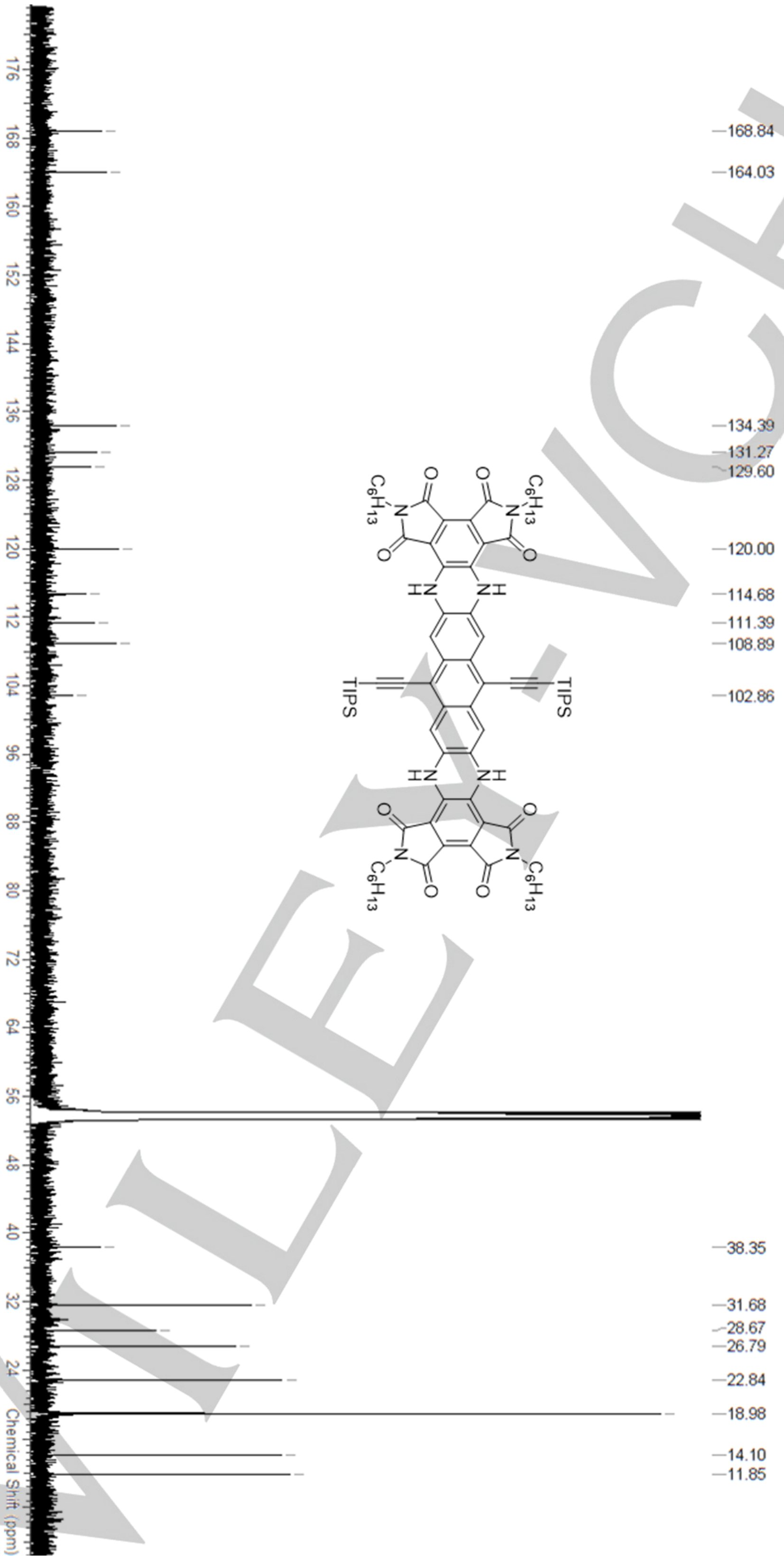

Figure S34. ^{13}C NMR spectrum (150.9 MHz, CD_2Cl_2, 295 K) of **12-H$_4$**.

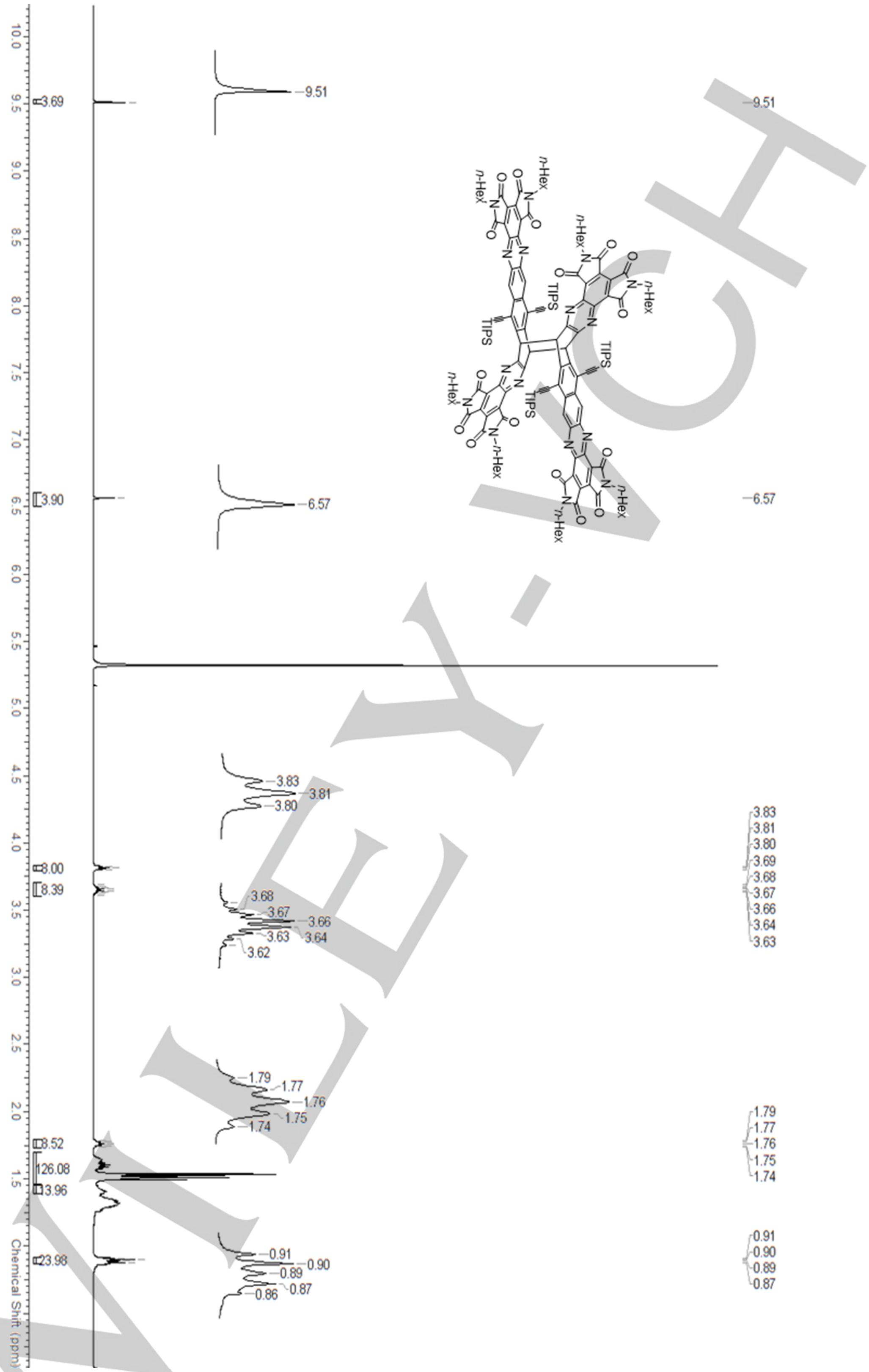

Figure S35. ^{1}H NMR spectrum (600.2 MHz, CD_2Cl_2, 295 K) of **13**.

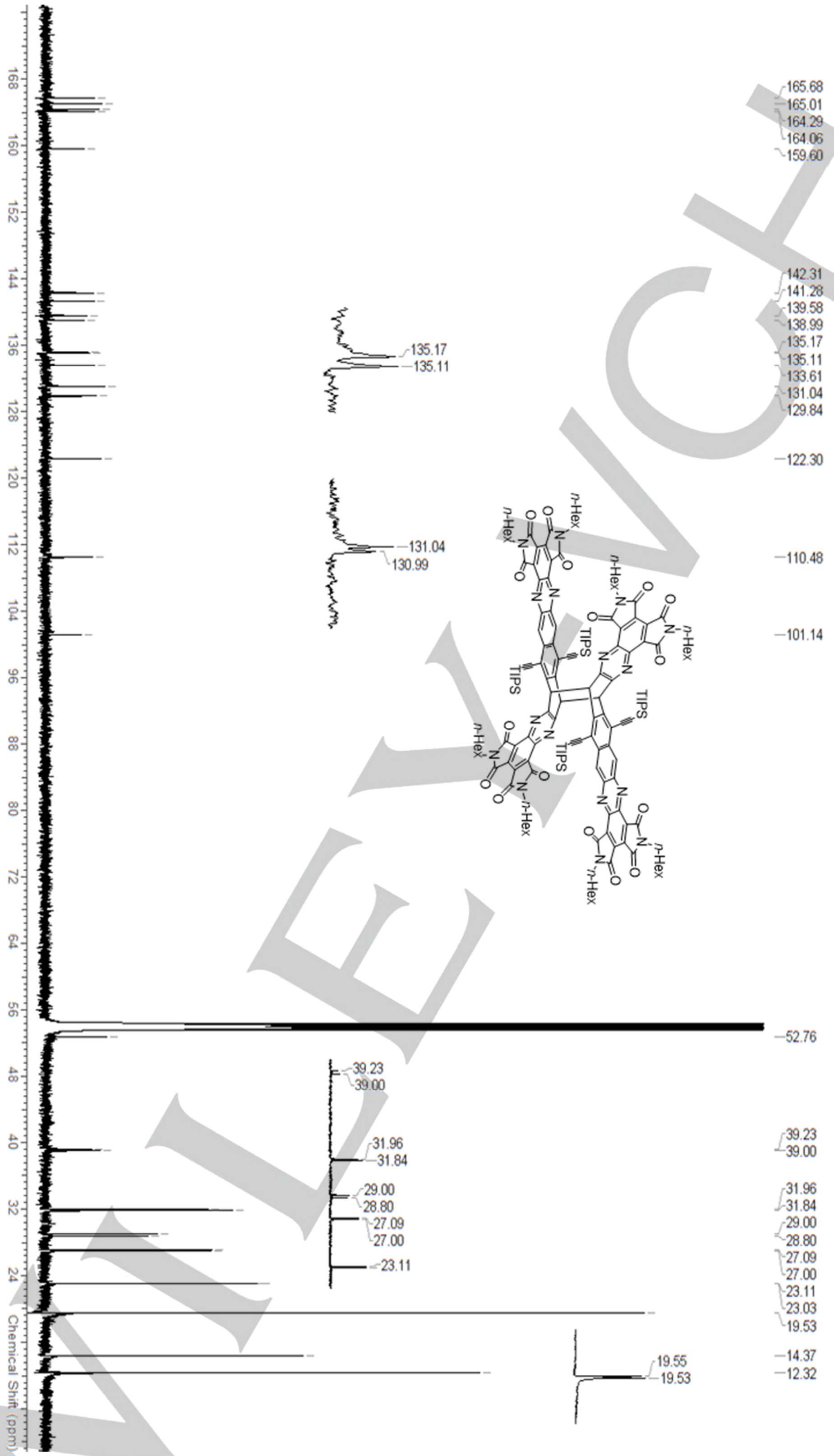

Figure S36. ^{13}C NMR spectrum (150.9 MHz, CD_2Cl_2, 295 K) of **13**.

WILEY-VCH

10) Infrared Spectroscopy

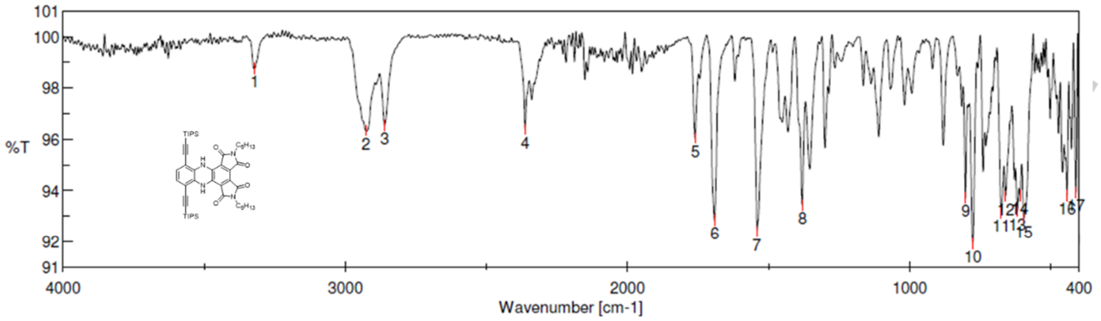

[Result of Peak Picking]

No.	Position	Intensity	No.	Position	Intensity	No.	Position	Intensity
1	3320.82	98.7553	2	2927.41	96.3397	3	2859.92	96.5383
4	2363.34	96.3919	5	1759.73	96.0567	6	1691.27	92.8266
7	1540.85	92.3953	8	1380.78	93.4277	9	802.242	93.706
10	776.208	91.9121	11	675.928	93.1158	12	660.5	93.7859
13	619.038	93.1844	14	609.396	93.7991	15	595.896	92.8882
16	442.583	93.7978	17	410.763	93.9281			

Figure S37. IR spectrum of **1-H$_2$**.

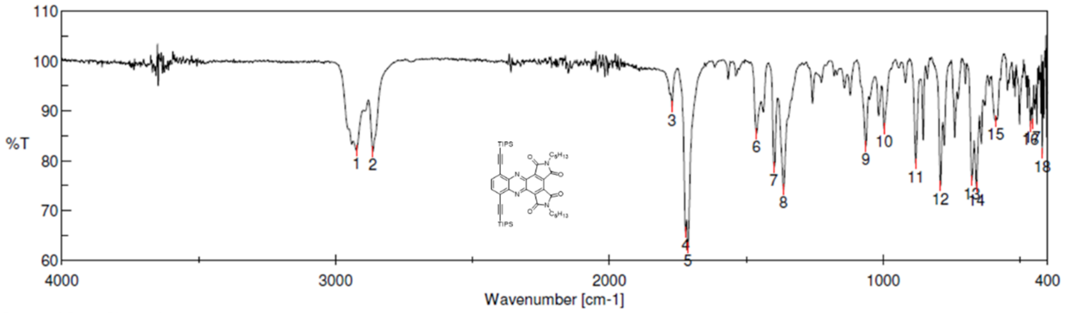

[Result of Peak Picking]

No.	Position	Intensity	No.	Position	Intensity	No.	Position	Intensity
1	2923.56	81.9642	2	2864.26	81.7604	3	1771.3	90.7616
4	1722.12	65.5947	5	1713.44	62.422	6	1463.22	85.3463
7	1398.14	78.6446	8	1364.39	74.2084	9	1064.03	82.8235
10	996.535	86.3487	11	881.309	79.335	12	791.154	74.9261
13	676.41	76.0239	14	659.536	74.7187	15	589.629	87.9094
16	462.832	86.7917	17	456.082	87.3883	18	418.959	81.4151

Figure S38. IR spectrum of **1a**.

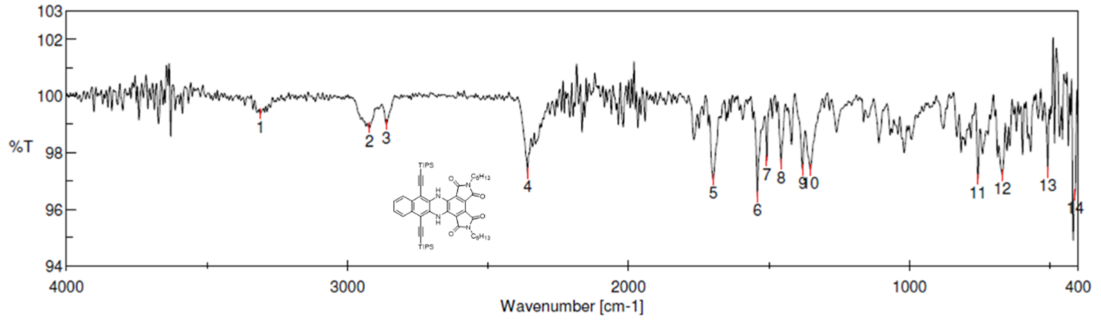

[Result of Peak Picking]

No.	Position	Intensity	No.	Position	Intensity	No.	Position	Intensity
1	3310.21	99.3708	2	2921.63	98.878	3	2861.84	99.0008
4	2359.48	97.2745	5	1698.98	97.0513	6	1540.85	96.4419
7	1508.06	97.7103	8	1456.96	97.5932	9	1380.78	97.3951
10	1352.82	97.3897	11	756.923	97.0663	12	670.142	97.2012
13	508.151	97.3101	14	410.763	96.5204			

Figure S39. IR spectrum of **2-H$_2$**.

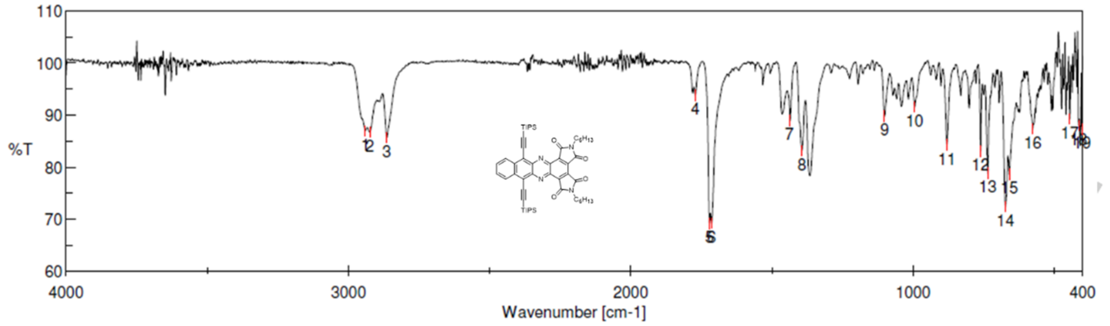

[Result of Peak Picking]

No.	Position	Intensity	No.	Position	Intensity	No.	Position	Intensity
1	2940.43	86.9497	2	2924.04	86.7366	3	2863.77	85.6955
4	1771.78	93.7463	5	1721.16	69.1743	6	1713.44	69.2362
7	1435.74	88.8654	8	1394.28	83.1623	9	1101.15	89.7607
10	995.089	91.4952	11	880.827	84.2593	12	761.262	83.044
13	735.71	78.8021	14	673.035	72.3606	15	659.536	78.5822
16	576.612	87.2962	17	444.994	89.185	18	414.138	87.9988
19	402.085	87.2334						

Figure S40. IR spectrum of **2a**.

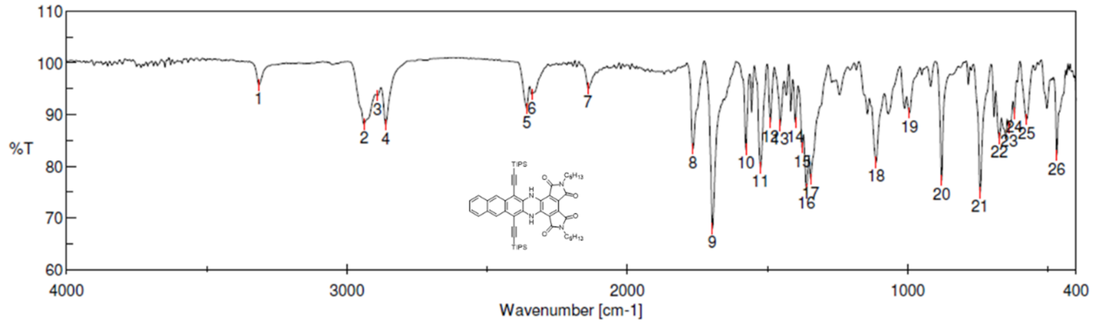

[Result of Peak Picking]

No.	Position	Intensity	No.	Position	Intensity	No.	Position	Intensity
1	3314.07	95.6373	2	2938.98	88.0774	3	2890.77	93.6185
4	2861.84	87.9901	5	2359.48	91.1174	6	2341.16	93.9018
7	2138.67	94.9573	8	1766.48	83.3297	9	1697.05	67.9341
10	1577.49	83.214	11	1525.42	79.6784	12	1489.74	88.4341
13	1455.03	87.8336	14	1400.07	88.4267	15	1375.96	83.5317
16	1361.5	75.5644	17	1346.07	77.4817	18	1112.73	80.69
19	994.125	90.2478	20	879.381	77.2266	21	742.46	74.8928
22	673.999	85.3269	23	638.323	87.4131	24	620.002	90.1609
25	576.612	88.9724	26	468.617	82.1453			

Figure S41. IR spectrum of **3-H$_2$**.

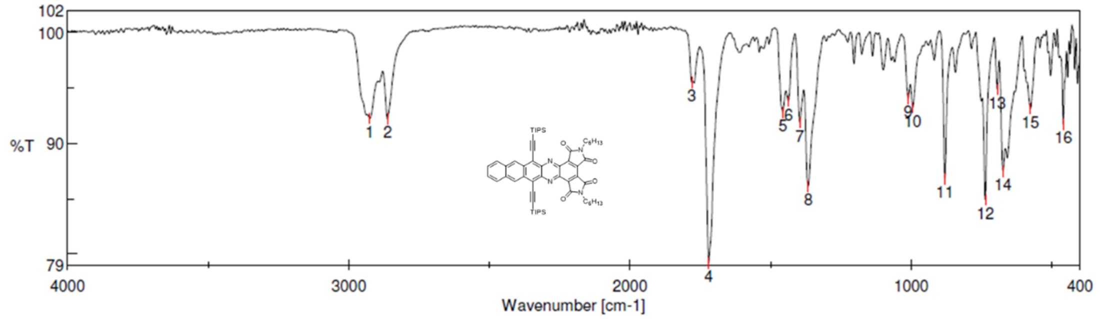

[Result of Peak Picking]

No.	Position	Intensity	No.	Position	Intensity	No.	Position	Intensity
1	2925.48	92.2517	2	2861.84	92.1915	3	1779.97	95.478
4	1720.19	79.1393	5	1456.96	92.8046	6	1436.71	93.7842
7	1396.21	91.8697	8	1366.32	86.136	9	1011.48	94.0489
10	995.089	93.2311	11	880.345	86.7738	12	736.674	84.9186
13	694.248	94.8604	14	673.999	87.4789	15	576.612	93.1231
16	458.975	91.725						

Figure S42. IR spectrum of **3a**.

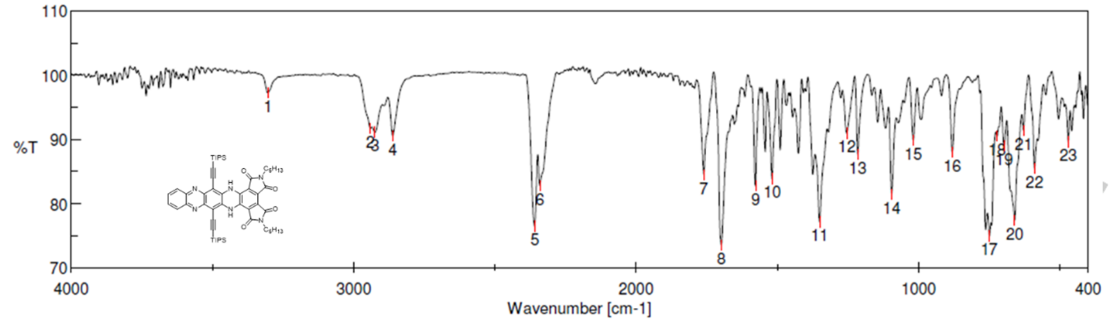

[Result of Peak Picking]

No.	Position	Intensity	No.	Position	Intensity	No.	Position	Intensity
1	3303.46	97.2234	2	2940.91	91.6739	3	2924.52	91.0895
4	2861.84	90.5185	5	2359.48	76.4724	6	2341.16	82.8256
7	1760.69	84.5204	8	1698.02	73.5422	9	1577.49	82.7484
10	1518.67	83.8857	11	1350.89	77.1786	12	1254.47	90.9326
13	1215.9	87.6476	14	1095.37	81.4682	15	1019.19	89.8423
16	881.309	88.0004	17	749.209	74.9364	18	722.211	90.4819
19	697.141	89.0074	20	660.5	77.4248	21	628.68	91.3582
22	590.111	85.4574	23	470.546	89.574			

Figure S43. IR spectrum of **4-H$_2$**.

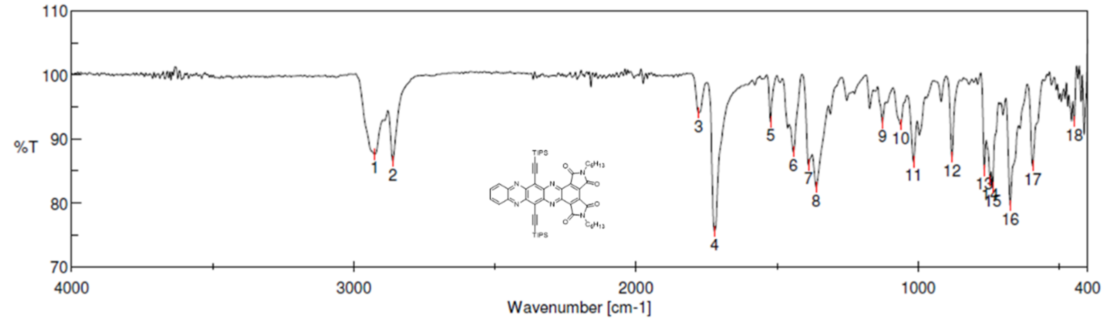

[Result of Peak Picking]

No.	Position	Intensity	No.	Position	Intensity	No.	Position	Intensity
1	2926.45	87.6087	2	2861.84	86.5203	3	1778.05	94.0546
4	1722.12	75.6055	5	1523.49	92.637	6	1442.49	87.9644
7	1388.5	85.9172	8	1361.5	82.4328	9	1127.19	92.4839
10	1062.59	92.2033	11	1016.3	86.3978	12	880.345	87.1677
13	765.601	85.0949	14	745.352	83.4405	15	736.674	82.3756
16	673.999	79.5803	17	594.932	85.7918	18	447.404	92.7508

Figure S44. IR spectrum of **4a**.

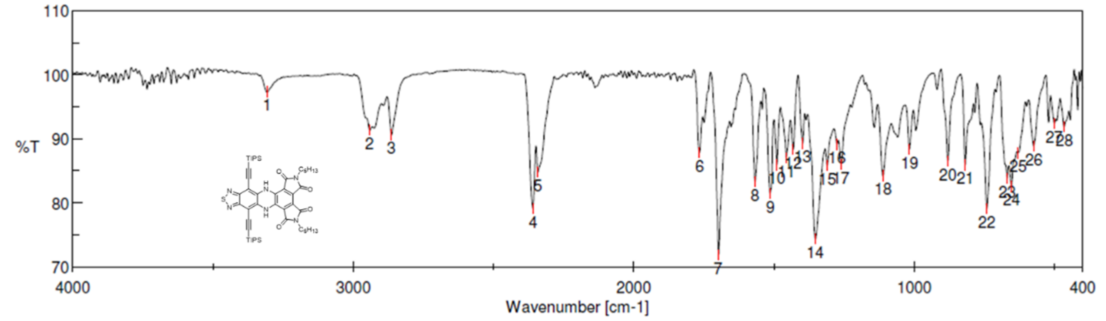

[Result of Peak Picking]

No.	Position	Intensity	No.	Position	Intensity	No.	Position	Intensity
1	3306.36	97.3065	2	2941.88	91.3367	3	2863.77	90.6013
4	2359.48	79.1247	5	2342.12	84.7792	6	1765.51	87.8111
7	1698.02	71.8954	8	1567.84	83.2873	9	1513.85	81.4623
10	1489.74	85.9409	11	1455.99	86.9807	12	1431.89	88.3428
13	1398.14	89.2698	14	1352.82	74.343	15	1310.39	85.7996
16	1275.68	89.1407	17	1260.25	85.9783	18	1111.76	84.1736
19	1017.27	88.3463	20	880.345	86.4841	21	818.634	85.8896
22	741.496	79.1037	23	669.178	83.7854	24	653.75	82.5108
25	630.609	87.7747	26	574.683	88.8269	27	500.437	92.4255
28	465.725	91.8434						

Figure S45. IR spectrum of **5-H$_2$**.

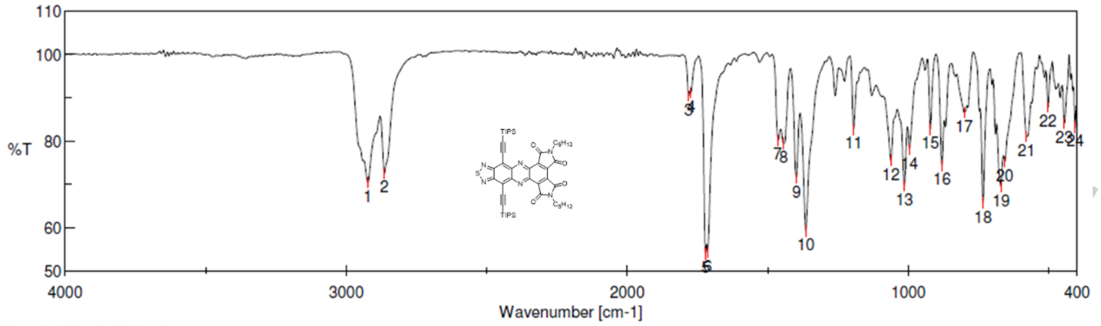

[Result of Peak Picking]

No.	Position	Intensity	No.	Position	Intensity	No.	Position	Intensity
1	2922.59	70.402	2	2863.77	72.5761	3	1781.9	90.3114
4	1773.23	91.1398	5	1721.16	53.897	6	1714.41	54.3319
7	1462.74	80.1608	8	1444.42	79.4319	9	1398.14	71.5143
10	1364.39	59.1763	11	1194.69	82.7121	12	1062.59	75.4899
13	1014.37	69.7066	14	996.053	77.9869	15	921.807	82.6295
16	880.345	74.3189	17	801.278	86.5824	18	734.746	65.7151
19	670.142	69.4951	20	656.643	75.2519	21	579.504	81.0331
22	503.33	87.802	23	445.476	84.0483	24	406.907	83.1132

Figure S46. IR spectrum of 5**a**.

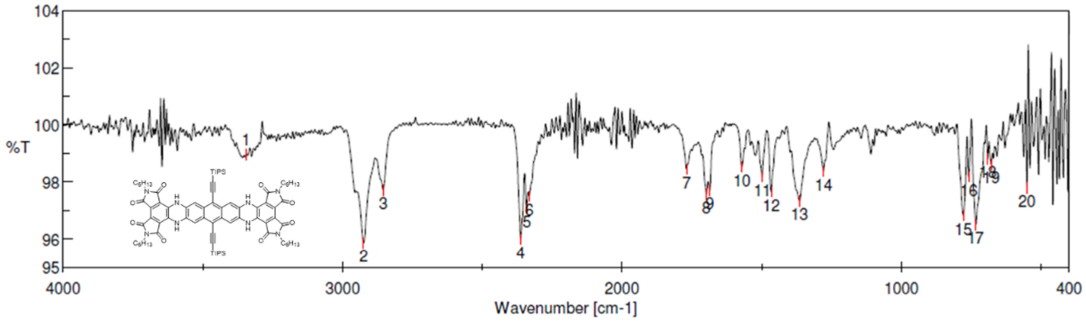

[Result of Peak Picking]

No.	Position	Intensity	No.	Position	Intensity	No.	Position	Intensity
1	3345.89	98.961	2	2924.52	95.8543	3	2854.13	97.7122
4	2362.37	95.9761	5	2341.16	97.0837	6	2331.52	97.4958
7	1769.37	98.4463	8	1698.02	97.5602	9	1685.48	97.7073
10	1570.74	98.5249	11	1498.42	98.1688	12	1465.63	97.6518
13	1364.39	97.3233	14	1278.57	98.3734	15	778.136	96.825
16	758.852	98.1678	17	733.782	96.5035	18	691.355	98.7947
19	678.82	98.6132	20	550.577	97.793			

Figure S47. IR spectrum of **12-H$_4$**.

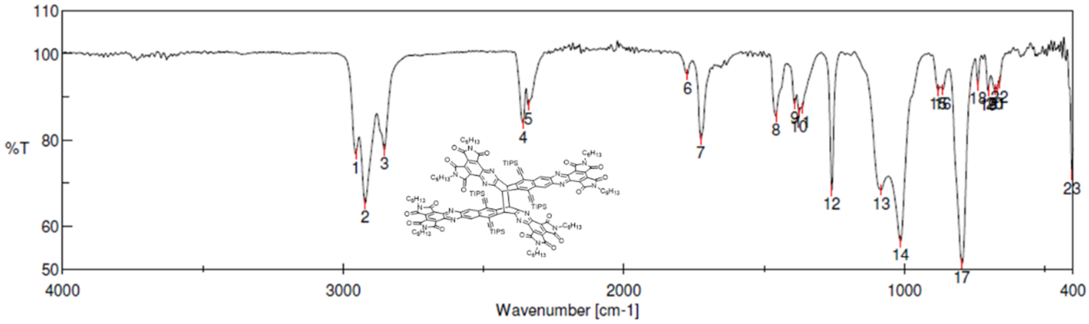

[Result of Peak Picking]

No.	Position	Intensity	No.	Position	Intensity	No.	Position	Intensity
1	2955.38	76.7972	2	2921.63	65.2209	3	2853.17	77.6025
4	2359.48	83.8094	5	2341.16	88.0455	6	1773.23	95.218
7	1725.01	80.0911	8	1457.92	85.472	9	1391.39	88.3124
10	1375.96	86.3874	11	1365.35	87.3911	12	1259.29	68.3174
13	1082.83	68.4421	14	1014.37	56.4204	15	880.345	91.4986
16	863.953	91.6685	17	795.493	51.3034	18	738.603	92.7915
19	701.962	91.4071	20	678.82	91.6348	21	670.142	91.597
22	661.464	93.1921	23	403.05	71.9212			

Figure S48. IR spectrum of **13**.

11) HRMS Spectra

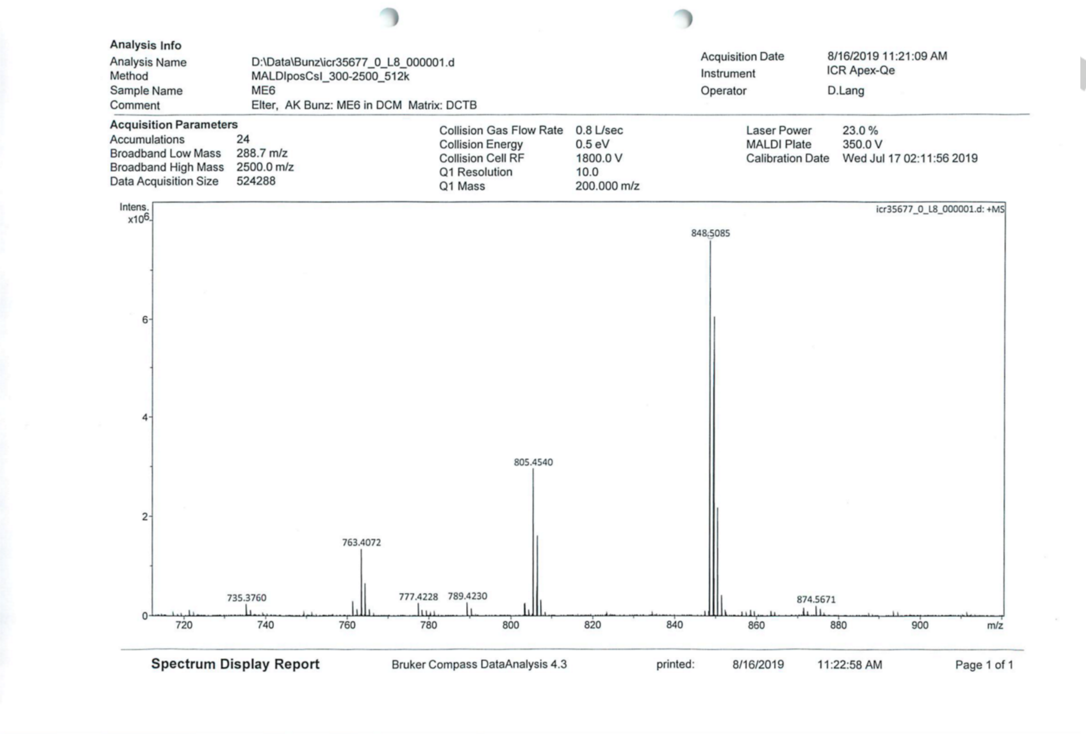

Figure S49: HRMS Spectrum of **1-H$_2$**.

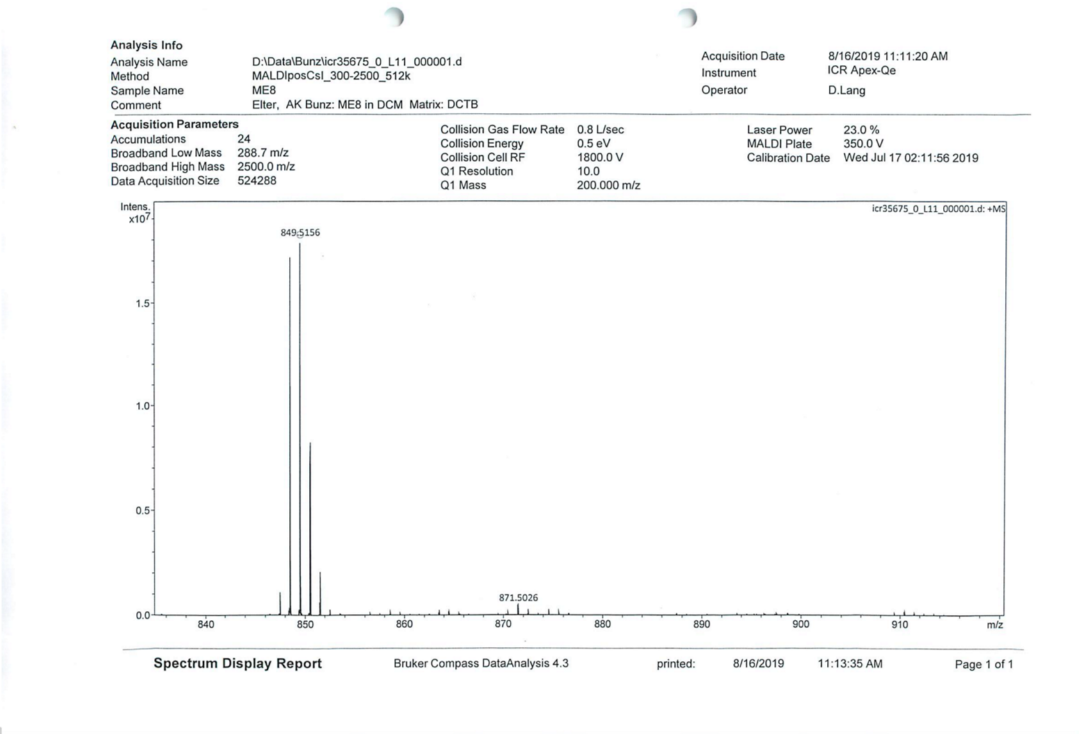

Figure S50: HRMS Spectrum of **1a**.

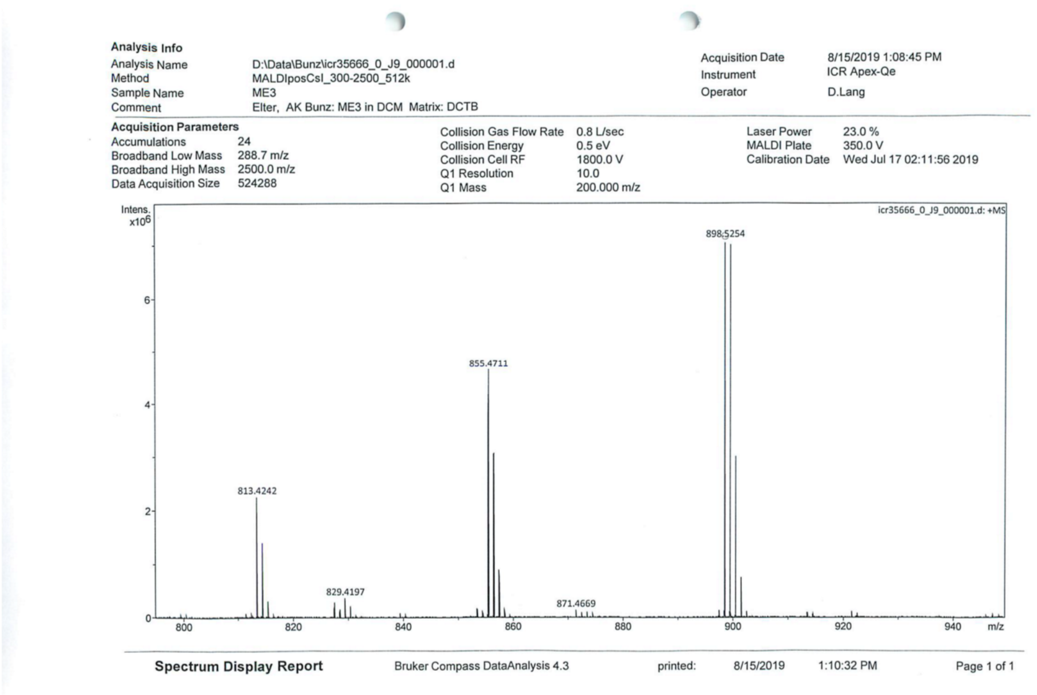

Figure S51: HRMS Spectrum of 2-H_2.

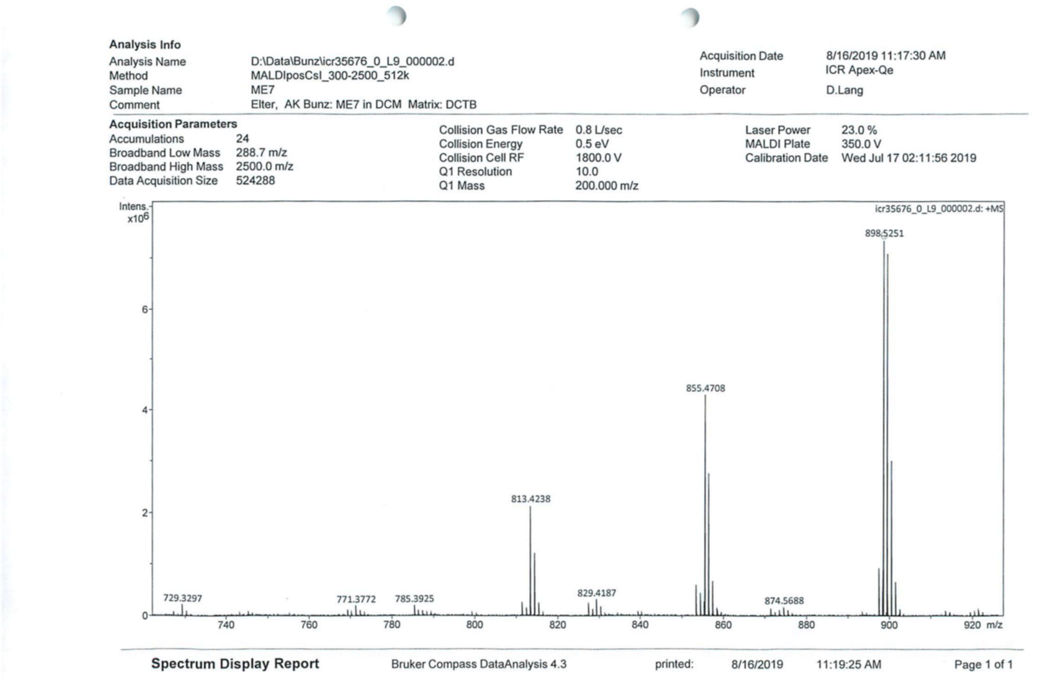

Figure S527: HRMS Spectrum of **2a**.

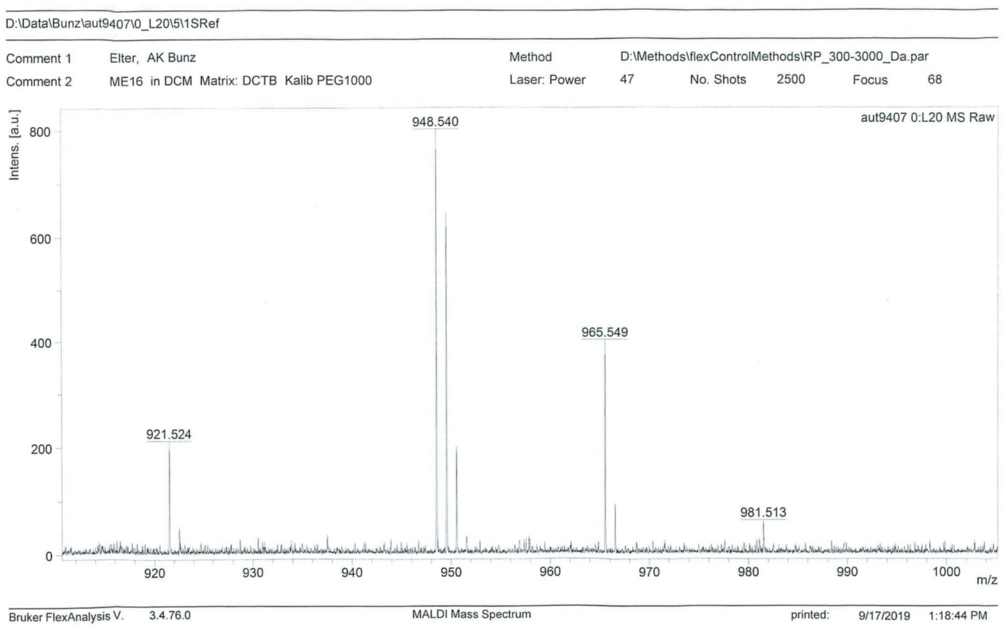

SmartFormula

Formula	Mass	Error	mSigma	DblEq	N rule	Electron Configuration
C 58 H 76 N 4 O 4 Si 2	948.5400	0.4355	69.2529	25.00	ok	odd

aut9407 / L20/5 Kalib PEG1000

Figure S53: HRMS Spectrum of **3-H$_2$** and attached HRMS Value.

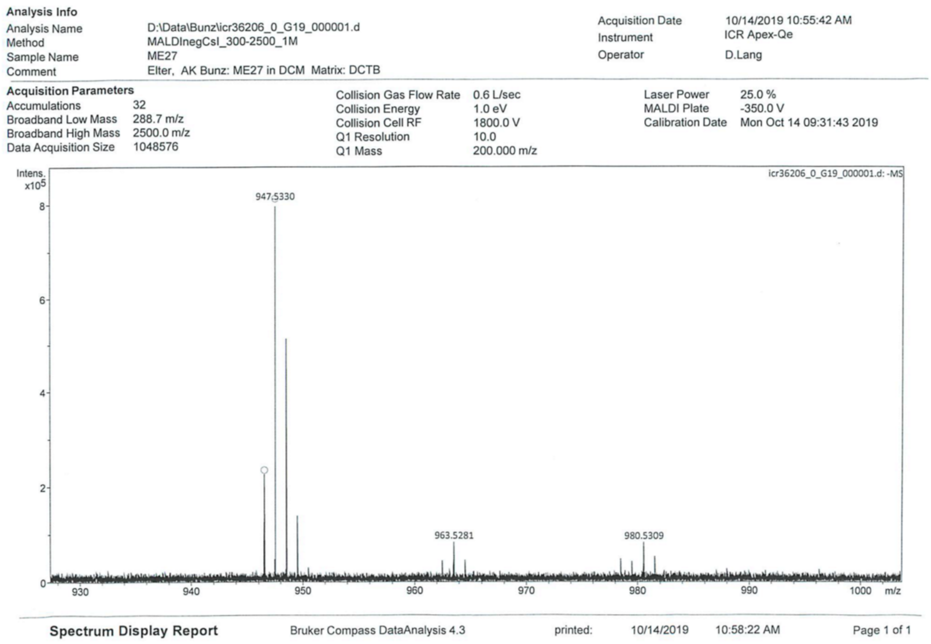

Figure S54: HRMS Spectrum of **3a**.

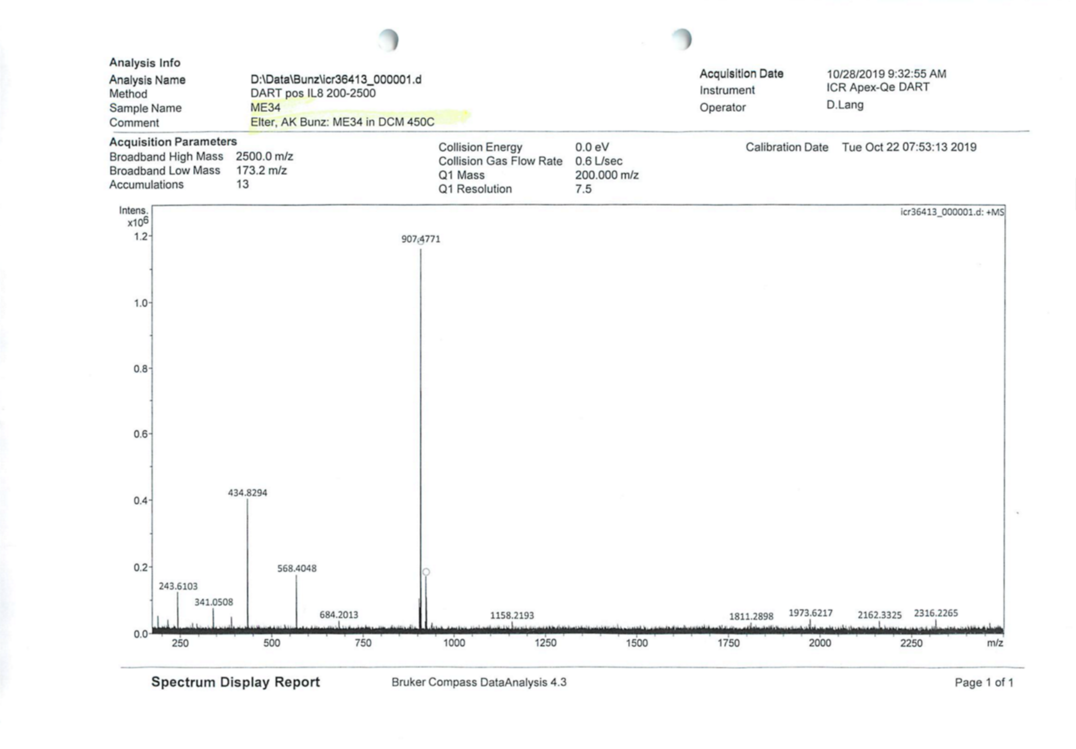

Figure S55: HRMS Spectrum of **4-H$_2$**.

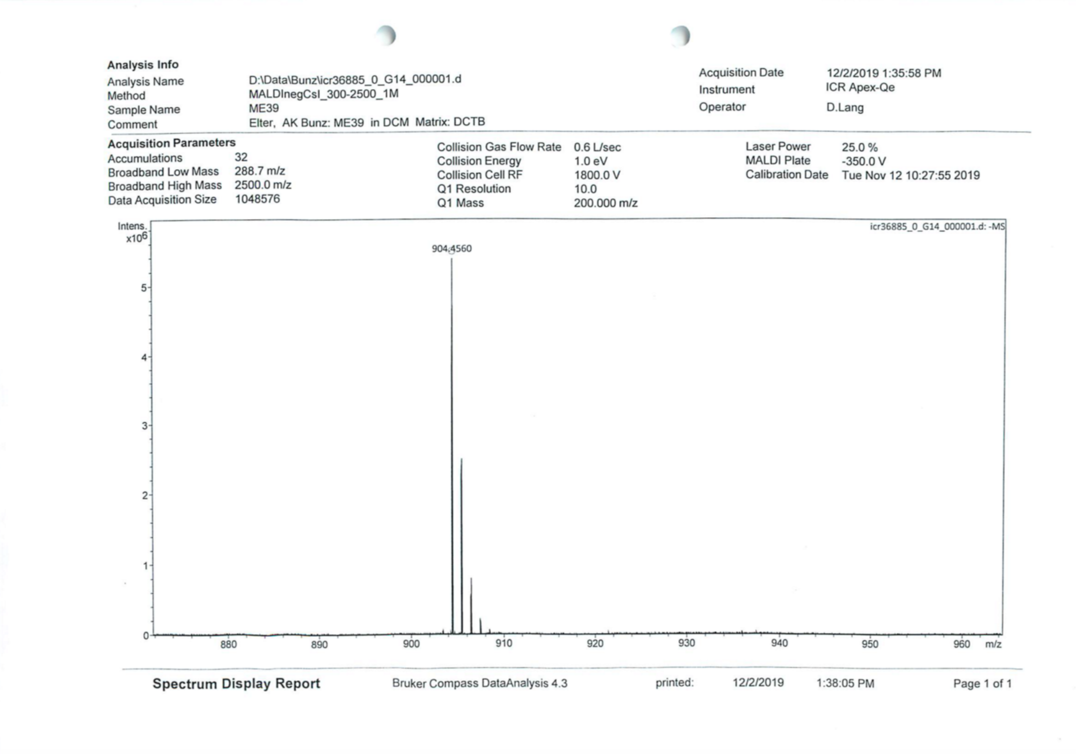

Figure S56: HRMS Spectrum of **4a**.

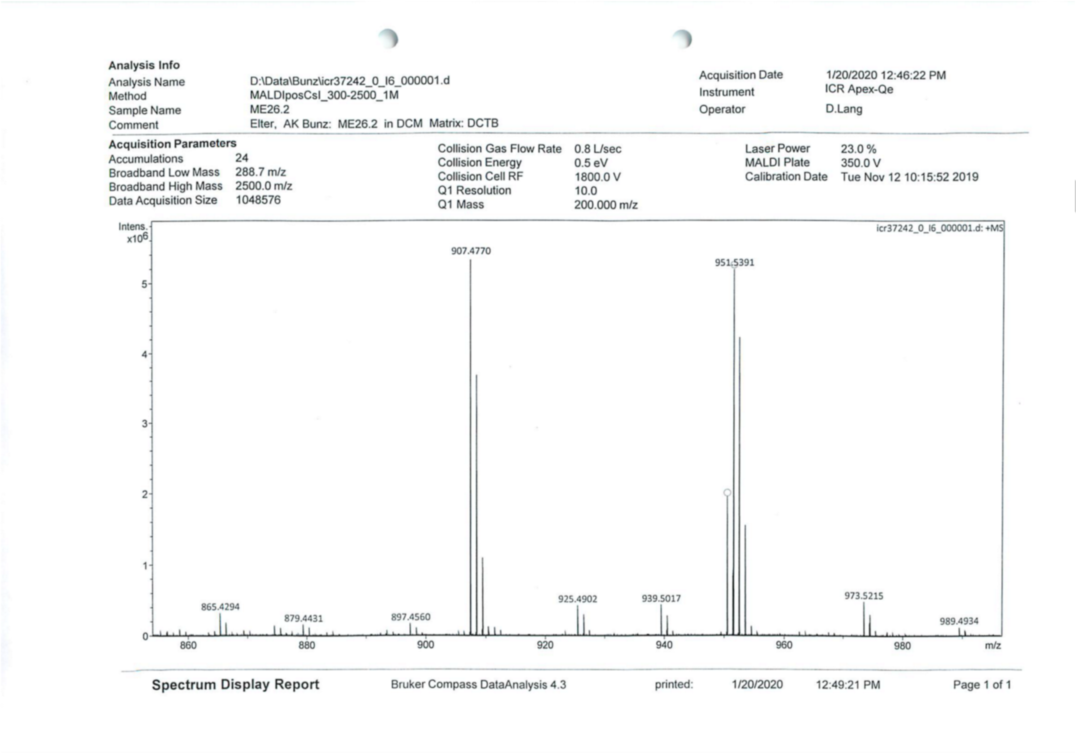

Figure S57: HRMS Spectrum of **5-H$_2$**.

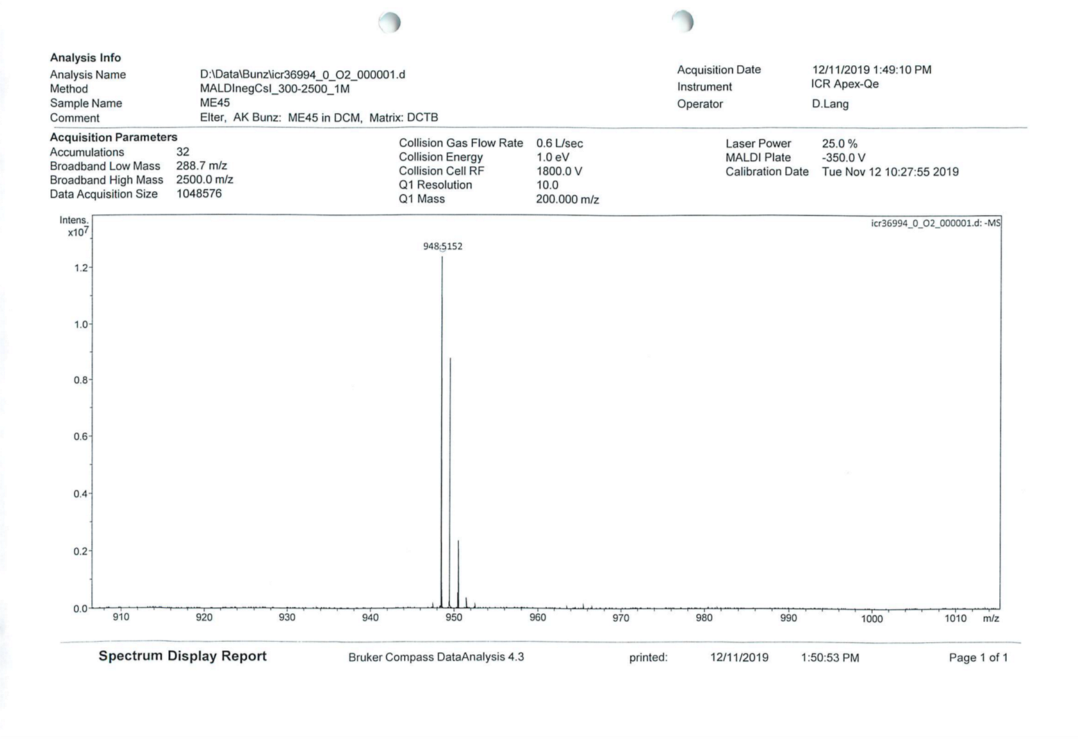

Figure S58: HRMS Spectrum of **5a**.

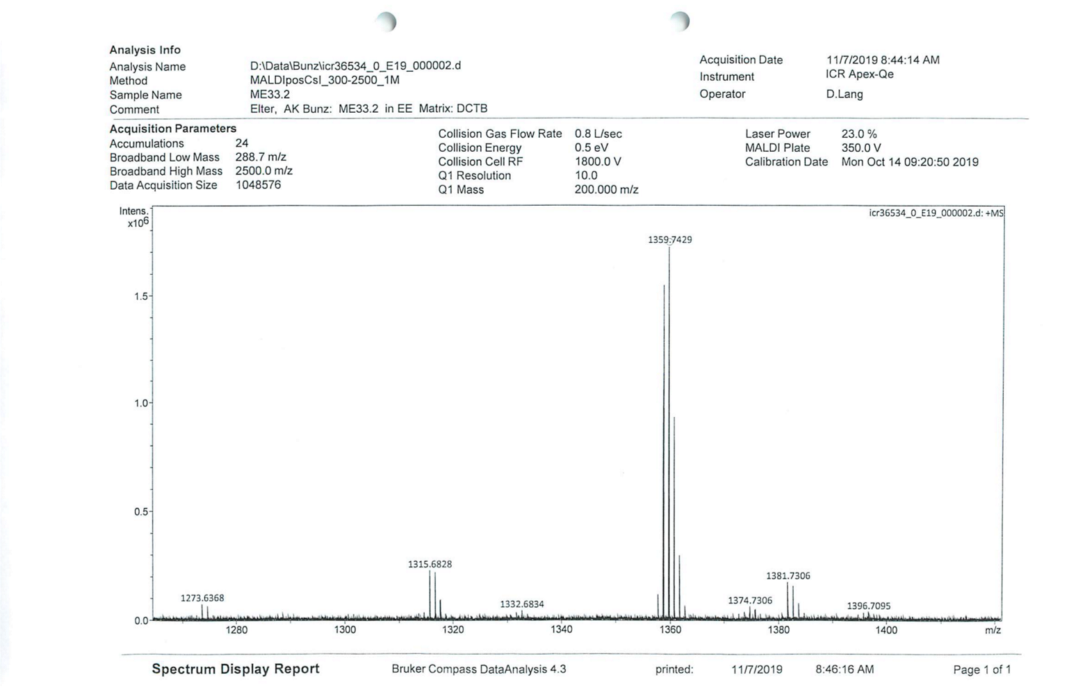

Figure S59: HRMS Spectrum of **12-H$_4$**.

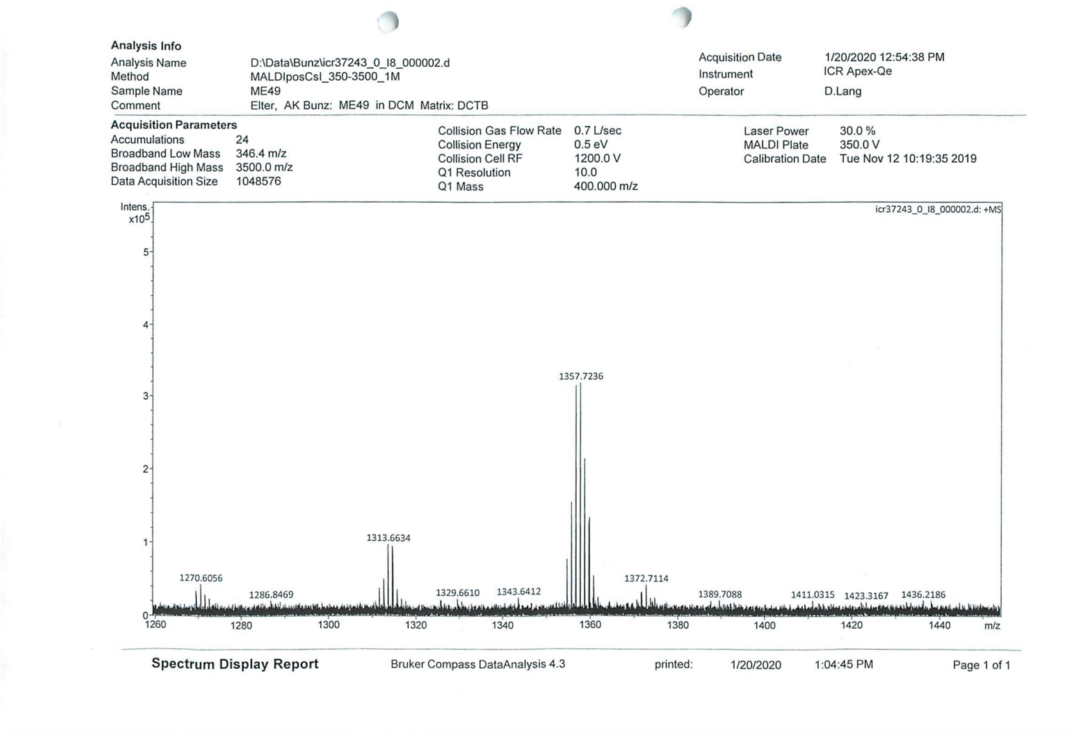

Figure S60: HRMS Spectrum of **13.**

12) Crystallographic Data

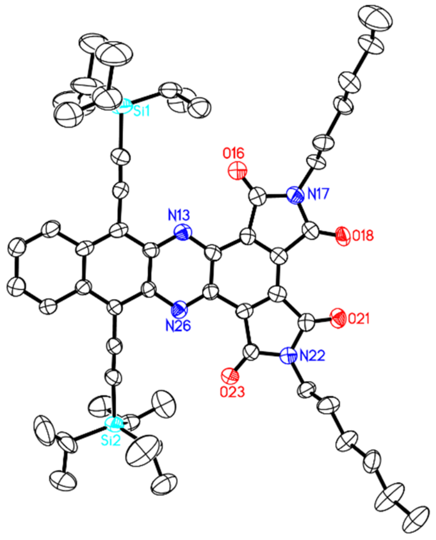

Table S7. Crystal data and structure of **2a**.

CCDC:	2080832	
Identification code	mel2	
Empirical formula	$C_{54}H_{72}N_4O_4Si_2$	
Formula weight	897.33	
Temperature	200(2) K	
Wavelength	1.54178 Å	
Crystal system	trigonal	
Space group	$P3_2$	
Z	3	
Unit cell dimensions	a = 22.6826(14) Å	α = 90 deg.
	b = 22.6826(14) Å	β = 90 deg.
	c = 8.7800(7) Å	γ = 120 deg.
Volume	3912.1(6) $Å^3$	
Density (calculated)	1.14 g/cm^3	
Absorption coefficient	0.98 mm^{-1}	
Crystal shape	needle	
Crystal size	0.141 x 0.021 x 0.021 mm^3	
Crystal colour	green	
Theta range for data collection	3.9 to 53.4 deg.	
Index ranges	$-23 \le h \le 23$, $-23 \le k \le 23$, $-5 \le l \le 9$	
Reflections collected	18747	
Independent reflections	4432 (R(int) = 0.1362)	
Observed reflections	3094 ($I > 2\sigma(I)$)	
Absorption correction	Semi-empirical from equivalents	
Max. and min. transmission	1.39 and 0.76	
Refinement method	Full-matrix least-squares on F^2	
Data/restraints/parameters	4432 / 498 / 592	
Goodness-of-fit on F^2	1.17	
Final R indices (I>2sigma(I))	R1 = 0.069, wR2 = 0.129	
Absolute structure parameter	0.22(11)	
Largest diff. peak and hole	0.37 and -0.31 $eÅ^{-3}$	

WILEY-VCH

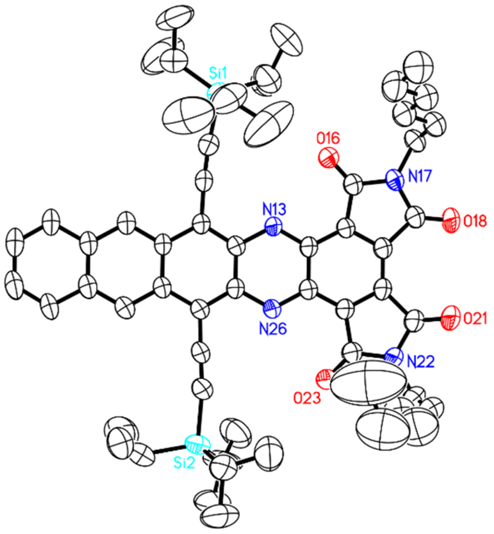

Table S8. Crystal data and structure of **3-H$_2$.**

CCDC:	2080833	
Identification code	mel4sq	
Empirical formula	$C_{58}H_{76}N_4O_4Si_2$	
Formula weight	949.40	
Temperature	200(2) K	
Wavelength	1.54178 Å	
Crystal system	triclinic	
Space group	P $\bar{1}$	
Z	2	
Unit cell dimensions	a = 12.4181(10) Å	α = 106.780(5) deg.
	b = 16.2442(12) Å	β = 100.328(6) deg.
	c = 17.1559(11) Å	γ = 105.110(6) deg.
Volume	3075.1(4) $Å^3$	
Density (calculated)	1.02 g/cm^3	
Absorption coefficient	0.85 mm^{-1}	
Crystal shape	brick	
Crystal size	0.170 x 0.035 x 0.031 mm^3	
Crystal colour	orange	
Theta range for data collection	3.0 to 60.0 deg.	
Index ranges	$-13 \le h \le 12$, $-18 \le k \le 15$, $-18 \le l \le 19$	
Reflections collected	23780	
Independent reflections	9042 (R(int) = 0.0625)	
Observed reflections	4643 (I > 2σ(I))	
Absorption correction	Semi-empirical from equivalents	
Max. and min. transmission	1.59 and 0.62	
Refinement method	Full-matrix least-squares on F^2	
Data/restraints/parameters	9042 / 2003 / 727	
Goodness-of-fit on F^2	1.02	
Final R indices (I>2sigma(I))	R1 = 0.078, wR2 = 0.199	
Largest diff. peak and hole	0.39 and -0.29 $eÅ^{-3}$	

WILEY-VCH

13) References

[S1] H. E. Gottlieb, V. Kotlyar, A. Nudelman, *J. Org. Chem.* **1997**, *62*, 7512-7515.

[S2] M. J. Frisch, G. W. Trucks, H. B. Schlegel, G. E. Scuseria, M. A. Robb, J. R. Cheeseman, G. Scalmani, V. Barone, G. A. Petersson, H. Nakatsuji, X. Li, M. Caricato, A. V. Marenich, J. Bloino, B. G. Janesko, R. Gomperts, B. Mennucci, H. P. Hratchian, J. V. Ortiz, A. F. Izmaylov, J. L. Sonnenberg, Williams, F. Ding, F. Lipparini, F. Egidi, J. Goings, B. Peng, A. Petrone, T. Henderson, D. Ranasinghe, V. G. Zakrzewski, J. Gao, N. Rega, G. Zheng, W. Liang, M. Hada, M. Ehara, K. Toyota, R. Fukuda, J. Hasegawa, M. Ishida, T. Nakajima, Y. Honda, O. Kitao, H. Nakai, T. Vreven, K. Throssell, J. A. Montgomery Jr., J. E. Peralta, F. Ogliaro, M. J. Bearpark, J. J. Heyd, E. N. Brothers, K. N. Kudin, V. N. Staroverov, T. A. Keith, R. Kobayashi, J. Normand, K. Raghavachari, A. P. Rendell, J. C. Burant, S. S. Iyengar, J. Tomasi, M. Cossi, J. M. Millam, M. Klene, C. Adamo, R. Cammi, J. W. Ochterski, R. L. Martin, K. Morokuma, O. Farkas, J. B. Foresman, D. J. Fox, Wallingford, *CT*, **2016**.

[S3] S. M. Luo, K. A. Stellmach, S. M. Ikuzwe, D. D. Cao, *J. Org. Chem.* **2019**, *84*, 10362-10370.

[S4] B. D. Lindner, J. U. Engelhart, M. Märken, O. Tverskoy, A. L. Appleton, F. Rominger, K. I. Hardcastle, M. Enders, U. H. F. Bunz, *Chem. Eur. J.* **2012**, *18*, 4627-4633.

[S5] S. Miao, S. M. Brombosz, P. v. R. Schleyer, J. I. Wu, S. Barlow, S. R. Marder, K. I. Hardcastle, U. H. F. Bunz, *J. Am. Chem. Soc.* **2008**, *130*, 7339-7344.

[S6] A. L. Appleton, S. M. Brombosz, S. Barlow, J. S. Sears, J.-L. Bredas, S. R. Marder, U. H. F. Bunz, *Nat. Commun.* **2010**, *1*, 91.

[S7] B. D. Lindner, J. U. Engelhart, O. Tverskoy, A. L. Appleton, F. Rominger, A. Peters, H.-J. Himmel, U. H. F. Bunz, *Angew. Chem. Int. Ed.* **2011**, *50*, 8588-8591; *Angew. Chem.* **2011**, *123*, 8747-8750.

[S8] C. An, X. Guo, M. Baumgarten, *Cryst. Growth Des.* **2015**, *15*, 5240-5245.

[S9] D. Xia, X. Guo, L. Chen, M. Baumgarten, A. Keerthi, K. Müllen, *Angew. Chem. Int. Ed.* **2016**, *55*, 941-944; *Angew. Chem.* **2016**, *128*, 953-956.

[S10] C. M. Cardona, W. Li, A. E. Kaifer, D. Stockdale, G. C. Bazan, *Adv. Mater.* **2011**, *23*, 2367-2371.

[S11] P. Biegger, S. Stolz, S. N. Intorp, Y. Zhang, J. U. Engelhart, F. Rominger, K. I. Hardcastle, U. Lemmer, X. Qian, M. Hamburger, U. H. F. Bunz, *J. Org. Chem.* **2015**, *80*, 582-589.

[S12] B. D. Lindner, F. Paulus, A. L. Appleton, M. Schaffroth, J. U. Engelhart, K. M. Schelkle, O. Tverskoy, F. Rominger, M. Hamburger, U. H. F. Bunz, *J. Mater. Chem. C* **2014**, *2*, 9609-9612.

[S13] S. Miao, A. L. Appleton, N. Berger, S. Barlow, S. R. Marder, K. I. Hardcastle, U. H. F. Bunz, *Chem. Eur. J.* **2009**, *15*, 4990-4993.

WILEY-VCH
